# Static Electron Correlation in Anharmonic Molecular Vibrations: A Hybrid TAO-DFT Study

**DOI:** 10.1021/acs.jpca.2c05881

**Published:** 2022-09-27

**Authors:** Magnus W. D. Hanson-Heine

**Affiliations:** School of Chemistry, University of Nottingham, University Park, NottinghamNG7 2RD, U.K.

## Abstract

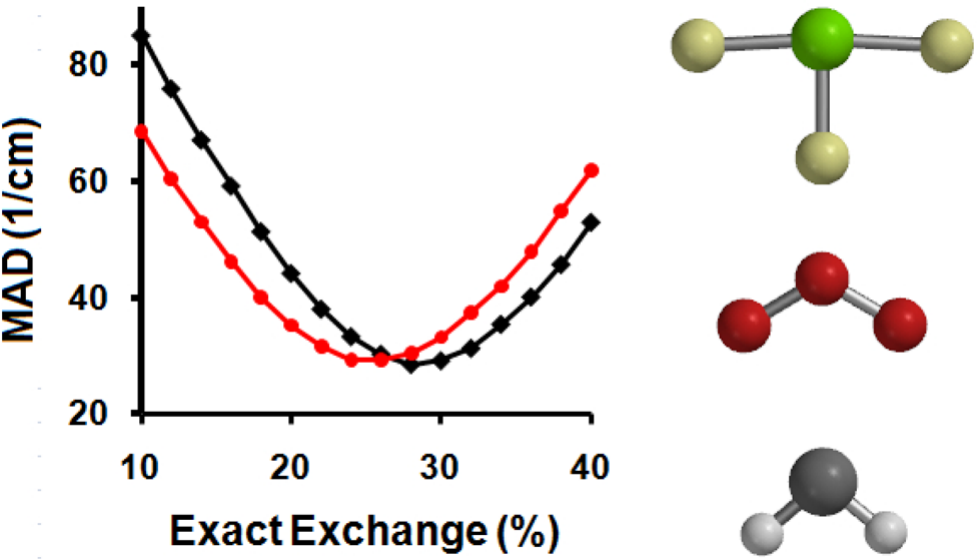

Hybrid thermally-assisted-occupation density functional theory is used to examine the effects of static electron correlation on the prediction of a benchmark set of experimentally observed molecular vibrational frequencies. The B3LYP and B97-1 thermally-assisted-occupation measure of static electron correlation is important for describing the vibrations of many of the molecules that make up several popular test sets of experimental data. Shifts are seen for known multireference systems and for many molecules containing atoms from the second row of the periodic table of elements. Several molecules only show significant shifts in select vibrational modes, and significant improvements are seen for the prediction of hydrogen stretching frequencies throughout the test set.

## Introduction

Calculations of molecular vibrational modes, zero-point energies, spectroscopic profiles, and the solutions to the nuclear Schrödinger equation are of significant importance in modern quantum chemistry. Experimental observations and theoretical models can be linked by accurately matching theoretically predicted and observed properties. The development of theoretical methods to efficiently and accurately predict these properties and the assessment of these methods is therefore an area of ongoing research,^1−6^ and molecular vibrational simulations have even recently been performed using blockchain computers.^7,8^

Representing the ground state wave function in the electronic Schrödinger equation as a single Slater determinant causes some of the electronic energy to be neglected. This missing energy is termed static correlation, also sometimes nondynamical, near-degeneracy, first-order, or strong correlation. Kohn–Sham density functional theory (KS-DFT) makes use of noninteracting auxiliary orbitals that are described by a single Slater determinant when constructing the one-electron probability density and therefore suffers from this kind of error. Most forms of KS-DFT have been found to perform poorly for systems that have are known to have “multireference” character, and attempts have been made to overcome this limitation.^9−16^ Thermally-assisted-occupation density functional theory (TAO-DFT) enables the calculation of static correlation within DFT through the use of fractional orbital occupations maintained with a fictitious temperature, θ.^9,17−19^ The complexity of this method scales similarly to KS-DFT when increasing the number of electrons in the system, and yet it has been shown to give a similar accuracy to computationally more expensive wave function based methods^17−29^ which scale very rapidly with increasing numbers of electrons.^30−32^

The dimension of the nuclear Schrödinger equation also increases rapidly as the number of nuclei in the molecular system increases, with vibrational frequency calculations often sampling electronic energies at multiple nuclear geometries to generate the potential energy surface (PES) within the Born–Oppenheimer approximation.^33^ PES sampling can be restricted to just nuclear second derivatives of the energy around an equilibrium geometry (the harmonic approximation),^34^ and the remaining anharmonic PES terms can be evaluated in the basis of normal coordinates along which the harmonic vibrations become independent. When anharmonic terms are included only up to the fourth-order nuclear derivatives, this is termed a quartic force field, or QFF, and further reductions are often made by only including derivatives that involve a specified number of different coordinates, termed an *n*-mode representation (*n*MR),^35^ or by changing the coordinates to find more disconnected terms that enable further reductions to be safely made.^36−57^

In spite of these layered approximations, the computational scaling of both the nuclear and electronic Schrödinger equations can make accurate anharmonic vibrational calculations prohibitively complex for larger molecules. The effects of static correlation on harmonic and anharmonic vibrations of large molecules are rarely considered. Despite this, recent studies have shown that vibrational frequency calculations can be significantly affectected by relatively small energy contributions, such as electron dispersion,^58^ and core-function contraction in electronic basis sets.^59^ The computational efficiency of TAO-DFT allows static correlation effects to be examined across a wide range of molecules and vibrations, to examine these effects in larger molecules, and to study vibrational anharmonicity in vibrational frequency data sets.

TAO-DFT has been described in detail elsewhere.^9,17,18^ However, TAO-DFT converges with KS-DFT in the limiting case when the fictitious temperature is zero.^9,17^ This means that comparisons can be made directly between the statically correlated and uncorrelated results within TAO-DFT. Newtonian nuclear motions have been calculated using *ab initio* molecular dynamics.^60^ Comparisons of vibrational frequencies have previously been made for the generalized gradient approximation (GGA) of DFT for the EDF1^61^ functional.^62^ However, GGA functionals perform poorly for describing experimental vibrational data due to partial neglect of dynamic electron correlation, i.e., the energy missing from Hartree–Fock (HF) theory that is not due to the use of a single determinant wave function.^63^ This reliance on the cancellation of different sources of error makes meaningful comparisons with experimental data difficult within the GGA. TAO-DFT for hybrid exchange-correlation functionals which include HF exchange have recently been implemented in the Q-Chem quantum chemistry software package.^64^ Hybrid KS-DFT can describe dynamic correlation well enough to enable closer and more meaningful comparisons with experimental data, and the B3LYP^65,66^ and B97-1^67^ exchange-correlation functionals are well suited to this kind of comparison.^63,68−74^ Here, the aim is to test whether differences between TAO-DFT and KS-DFT vibrational data are present for hybrid DFT methods and whether hybrid TAO-DFT can capture effects present in the experimental data.

## Computational Details

Geometry optimization and vibrational frequency calculations were performed using the Q-Chem quantum-chemical software package with the TAO-B3LYP and TAO-B97-1 methods at a range of fictitious temperatures outlined in the text.^9^ The corresponding KS-B3LYP and KS-B97-1 methods were also used, and all DFT methods were combined with the 6-311++G(d,p) and aug-cc-pVTZ electronic basis sets and Euler-Maclaurin-Lebedev EML-(100,302) numerical integration grid. A more detailed discussion of integration grids can be found elsewhere.^75−77^

Initial molecular structures were optimized to minimum energy geometries at each level of theory, and the resulting DFT force fields were used for the harmonic and anharmonic calculations. While analytical geometric second derivatives are not currently available for the TAO-DFT energy expression, second- and higher-order energy derivatives can be constructed from finite difference calculations of lower-order analytical derivatives in a straightforward manner. Harmonic vibrational frequencies and normal modes were determined using finite differences of the analytical nuclear first derivatives of the energy with a step size of 0.001 Å for all of the methods studied. The anharmonic PES was constructed within a Taylor series *n*MR representation of the quartic force field including up to two mode-coupling terms.^35^ The third- and fourth-order derivatives of the PES were calculated by numerical differentiation of analytical first derivatives with a step size of 0.5291 Å along each harmonic normal coordinate. The anharmonic frequencies were then calculated using the transition-optimized shifted Hermite (TOSH) variation of second-order vibrational perturbation theory (VPT2) in order to avoid issues with degenerate modes,^78^ and the effects of both two-mode and three-mode coupling terms were also investigated using unmodified VPT2.

These calculations have been evaluated using common molecular benchmark sets of experimental frequencies. Initial assessment of the fictitious temperature was carried out using the F38 molecular test set of experimentally determined harmonic frequencies.^69^ Fundamental anharmonic vibrational transitions were then determined for a reduced version of the F1 frequency set used in the harmonic frequency scaling factor work by the Radom group.^79,80^ This modified anharmonic F1 set is shown in Table 1(58,59) and was restricted to reduce the number of molecules and modes to a more computationally manageable number while focusing on reducing the number of “floppy” (nonsemirigid) molecules and molecules containing molecular rotors and high symmetry axes, which are known to cause problems matching with experiment under certain conditions.^78^ The HCCCCH, CSCl_2_, and CH_2_CCH_2_ molecules were also removed due various convergence issues or anomalous results during the TAO-DFT calculations. The vibrational modes for each molecule are numbered in order of their ascending harmonic transition energies. Mode with energies below 300 cm^–1^ have been treated as having either floppy or rotational character and excluded from the QFF. The following low-frequency modes have been removed from the anharmonic experimental test set in order to increase consistency across the anharmonic methods tested: ClF_3_, modes 1–2; ClSN, mode 1; NCl_2_F, mode 1; Cl_2_O, mode 1; SOCl_2_, modes 1–2; SCl_2_, mode 1; SOCl_2_, modes 1–2; SCl_2_, mode 1; S_2_F_2_, modes 1–3; COCl_2_, mode 1; C_2_Cl_2_, modes 1–2; C_2_N_2_, modes 1–2; *trans*-CHClCHCl, modes 1–2; *cis*-CHClCHCl, mode 1; CH_2_CCl_2_, mode 1; *cis*-CHFCHF, mode 1; *trans*-OCHCHO, mode 1; CH_2_CCHCl, mode 1; CH_2_CHCHO, mode 1; CH_2_CHCHCH_2_, modes 1–2.

**Table 1:** Modified Anharmonic F1 Set Used Here

3-atom molecules	4-atom molecules	larger molecules
^1^CH_2_	C_2_Cl_2_	*cyclo*-C_2_H_4_NH
^3^CH_2_	C_2_N_2_	*cyclo*-C_2_H_4_O
Cl_2_O	ClF_3_	*cyclo*-C_3_H_6_
ClCN	ClNO_2_	CH_2_CCHCl
ClNO	COCl_2_	CH_2_CCl_2_
ClSN	COClF	CH_2_CHCHCH_2_
CO_2_	COF_2_	CH_2_CH_2_
COS	CSF_2_	CH_2_CHCHO
CS_2_	F_2_NH	*cis*-CHClCHCl
F_2_O	F_2_SO	*cis*-CHFCHF
FCN	H_2_CO	HCOOH
H_2_O	H_2_O_2_	HNO_3_
H_2_S	H_2_S_2_	*trans-*OCHCHO
HCN	HCCCl	*trans*-CHClCHCl
HCO	HCCF	*trans*-CHFCHF
HOCl	HCCH	
HOF	HN_3_	
N_2_O	HNCO	
NO_2_	N_2_F_2_	
NSF	NCl_2_F	
O_3_	NClF_2_	
ONF	S_2_F_2_	
SCl_2_	SOCl_2_	
SO_2_		

The frequency ranges used in this work are quoted with respect to the experimentally observed fundamental transitions so as to be consistent across the different methods used. Mean absolute deviations (MADs) between the different kinds of calculated vibration and also between the calculated and experimental transitions have been reported for convenience, and frequencies have been grouped into three ranges based on their experimental values of between 300 and 1000 cm^–1^, greater than 1000 cm^–1^ but less than 2600 cm^–1^, and greater than 2600 cm^–1^. The value of 2600 cm^–1^ was chosen here to include the S–H stretching modes of H_2_S_2_ in the higher energy bracket that is commonly associated with hydrogen stretching motions. A complete set of the calculated transition frequencies can be found in the Supporting Information.

## Results and Discussion

### A Fictitious Temperature

The fractional orbital occupation numbers in a TAO-DFT calculation are given by the Fermi–Dirac distribution function with a fictitious temperature parameter, θ, corresponding to the temperature of the noninteracting reference system used to model the real system at a temperature of absolute zero. This reference system reduces to the Kohn–Sham reference system for the corresponding exchange-correlation functional when the fictitious temperature parameter is zero. Chai and co-workers have determined that the optimal system-independent fictitious temperature for each functional is closely linked to the fraction of exact orbital exchange used in the parametrization of the Kohn–Sham exchange energy functional and that the optimal fictitious temperature is only negligibly effected by the other aspects of the underlying energy functional.^18,19^ They used numerical fitting techniques to determine three potential schemes for calculating the optimal fictitious temperature for a given exchange-correlation functional. These are given here in units of mE_h_ and with the fraction of exact exchange denoted as *a*_*x*_
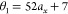
1
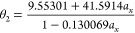
2and
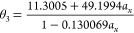
3The fictitious temperature in TAO-DFT is chosen so that the distribution of orbital occupation numbers closely matches the distribution of the corresponding natural orbital occupation numbers (NOONs) for the system in question.^18^ For single-reference systems, the exact NOONs are close to either 0 or 1, and the optimal fictitious temperature should therefore be relatively small. However, for multireference systems, the distribution of NOONs can span a wide range of values due to the varying strength of static correlation, and the corresponding optimal fictitious temperature therefore also spans a wide range of values. This implies that it is not possible to define a single fictitious temperature in global hybrid TAO-DFT which is optimal for both single-reference and multireference systems.^18^ It can be useful to define an optimal system-independent fictitious temperature for a global hybrid functional in order to provide an explicit description of orbital occupations, and the numerical schemes given above have been proposed to do that. However, molecular systems that are predominantly single-reference near their equilibrium nuclear geometries can become significantly multireference at nonequilibrium geometries.^81−83^ A singular fictitious temperature in TAO-DFT may therefore be unable to accurately model properties that depend on accurately representing multiple regions of the nuclear PES at a consistent level of theory.

While the search for more accurate theoretical and numerical parameters for the fictitious temperature in TAO-DFT is ongoing, the schemes given have shown promising results for a wide range of calculations and have been tested here for the B3LYP and B97-1 functionals with the aug-cc-pVTZ basis set in order to determine which fictitious temperature equation proves to be the most accurate for describing harmonic molecular vibrational frequencies with these models. The F38 molecular test set of experimentally determined harmonic frequencies has been used as a benchmark for these tests,^69^ and the results are given here in Table 2. The standard KS-DFT implementation of these functionals corresponding to a value of *θ* = 0 in the TAO-DFT framework is denoted *θ*_*0*_.

**Table 2:** Molecular and Total MADs (in cm^–1^) between the Calculated and Experimental Harmonic Frequencies for the Molecules and Reference Data in the F38 Benchmark Set^a^

	B3LYP				B97-1			
	*θ*_*0*_	*θ*_*1*_	*θ*_*2*_	*θ*_*3*_	*θ*_*0*_	*θ*_*1*_	*θ*_*2*_	*θ*_*3*_
H_2_	16.7	45.8	48.9	60.7	32.7	62.6	65.5	77.6
CH_4_	20.7	17.5	17.2	16.0	25.2	17.8	17.5	16.2
NH_3_	24.1	19.4	19.9	22.0	17.5	18.6	19.1	21.2
H_2_O	33.9	23.6	22.6	19.0	8.9	8.5	9.2	12.0
HF	66.6	53.2	51.8	46.7	20.1	6.3	5.0	0.2
CO	37.9	42.2	42.6	43.9	35.9	40.4	40.8	42.1
N_2_	88.4	93.2	93.7	95.2	73.2	78.2	78.7	80.1
F_2_	135.8	134.9	133.4	123.5	154.5	154.1	152.8	143.9
C_2_H_2_	29.9	36.1	36.7	38.7	23.7	26.8	27.4	29.3
HCN	35.4	42.5	43.2	45.5	32.3	34.5	35.2	37.4
H_2_CO	31.7	25.8	25.0	21.5	36.2	30.1	29.3	25.8
CO_2_	8.4	12.2	12.6	13.7	12.7	16.5	16.9	17.9
N_2_O	34.9	38.6	38.8	38.8	29.1	32.9	33.1	32.9
Cl_2_	20.8	31.8	35.5	53.4	1.6	8.4	11.4	27.9
OH	44.7	26.8	25.3	19.7	9.4	7.3	8.8	14.3
MAD	33.4	33.2	33.3	33.4	28.5	28.8	29.1	30.0
MAD_low_	35.9	37.3	37.4	37.5	33.0	34.2	34.3	34.3
MAD_high_	29.0	26.2	26.2	26.3	20.8	19.5	20.1	22.5
MAD′_high_	30.0	24.6	24.4	23.7	19.9	16.2	16.6	18.3

a The “high” and “low” MAD values correspond to frequencies above and below 2600 cm^–1^. The prime symbol (′) indicates the exclusion of H_2_.

The B3LYP functional shows a slight improvement in the overall MAD when using the TAO-DFT implementation and gets progressively worse as the larger fictitious temperatures are used. This is due to deterioration in the predictive quality of the vibrational modes that do not contain significant hydrogen stretching character and improvements in the 14 modes that do contain predominantly hydrogen stretching character. The H_2_ molecule in particular has an above average error for the TAO-DFT calculations, and the MADs have therefore also been provided for the H-X stretching transitions with H_2_ excluded, denoted by the prime symbol. Despite giving a slightly worse overall prediction compared to the *θ*_*1*_ fictitious temperature, the *θ*_*2*_ formulation is considered to be preferable here, due to having a more rigorous parametrization. The use of *θ*_*1*_ is included primarily for legacy purposes and making comparisons with pre-existing data. The *θ*_*3*_ fictitious temperatures give harmonic frequencies with lower accuracy compared to the other combinations tested for both the B3LYP and B97-1 functionals and have been noted as particularly effective for more significantly multireference systems and properties. The *θ*_*2*_ fictitious temperature values have therefore been used for the TAO-DFT calculations throughout the rest of this study.

### B Anharmonic Fundamental Transitions

**Figure 1:**
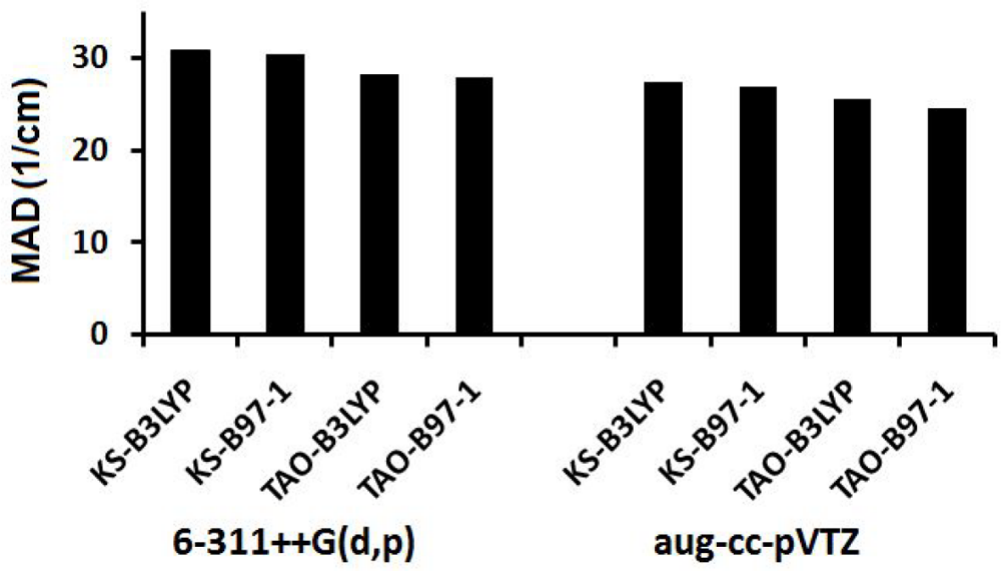
MAD values between the calculated anharmonic frequencies and experimental frequencies of all modes in the anharmonic test set.

**Figure 2:**
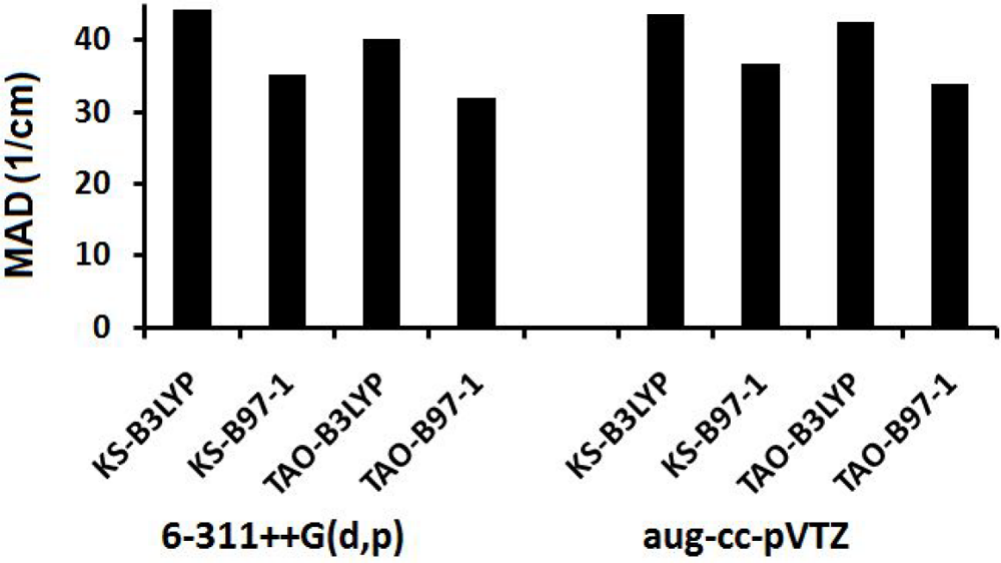
MAD values between the calculated anharmonic frequencies and experimental frequencies of modes above 2600 cm^–1^ in the anharmonic test set.

Anharmonic experimental MADs for the KS-DFT and TAO-DFT fundamental transition frequencies calculated with the B3LYP and B97-1 exchange-correlation functionals and both the 6-311++G(d,p) and aug-cc-pVTZ basis sets are shown in Table 3, Figure 1, and Figure 2. The average agreement with experiment for vibrational transitions with frequencies below 2600 cm^–1^ is consistently found to be ca. 1–2 cm^–1^ worse for the TAO-DFT calculations compared with KS-DFT. The transition energies themselves are shifted by an average of ca. 3–5 cm^–1^ in these spectral regions (see Table 4), indicating that there are significant improvements in some modes and deterioration in others. The experimental match for the higher energy vibrational transitions with frequencies above 2600 cm^–1^ is improved by ca. 7–9 cm^–1^ on average when using TAO-DFT. Vibrational modes in the frequency ranges 300–1000, 1000–2600, and >2600 cm^–1^ have MADs from experiment of ca. 23, 22, and 43 cm^–1^, respectively, for the KS-DFT calculations, and ca. 22, 23, and 34 cm^–1^, respectively, for the TAO-DFT calculations at the B97-1/aug-cc-pVTZ level.

**Table 3:** MAD Values in cm^–1^ from Experiment for the Calculated Anharmonic KS-DFT and TAO-DFT Frequencies for Different Frequency Ranges in the Anharmonic Set

functional	basis set	all modes	300–1000 cm^–1^	1000–2600 cm^–1^	2600+ cm^–1^
KS-B3LYP	6-311++G(d,p)	30.9	29.6	26.3	44.2
TAO-B3LYP	6-311++G(d,p)	30.4	30.9	27.7	35.2
KS-B97-1	6-311++G(d,p)	28.3	27.2	24.3	40.1
TAO-B97-1	6-311++G(d,p)	27.9	28.0	25.9	32.0
KS-B3LYP	aug-cc-pVTZ	27.4	24.1	24.5	43.6
TAO-B3LYP	aug-cc-pVTZ	26.9	24.5	25.7	36.7
KS-B97-1	aug-cc-pVTZ	25.5	22.5	21.9	42.6
TAO-B97-1	aug-cc-pVTZ	24.6	22.4	23.3	33.9

**Table 4:** MADs in cm^–1^ between the KS-DFT and TAO-DFT Values for the Calculated Anharmonic Frequencies across Different Frequency Ranges in the Anharmonic Set

functional	basis set	all modes	300–1000 cm^–1^	1000–2600 cm^–1^	2600+ cm^–1^
B3LYP	6-311++G(d,p)	5.9	3.5	4.6	15.4
B97-1	6-311++G(d,p)	5.8	3.3	4.5	15.6
B3LYP	aug-cc-pVTZ	5.6	3.0	4.4	15.8
B97-1	aug-cc-pVTZ	5.5	2.8	4.4	15.6

Overall the net effect is a slight improvement in the experimental match for both functional and basis set combinations when using TAO-DFT. The B97-1 functional consistently outperforms the B3LYP functional in both the KS-DFT and TAO-DFT frameworks, and the overall MADs from experiment across all of the modes drop from 27.4 to 26.9 cm^–1^ for the B3LYP functional and from 25.5 to 24.6 cm^–1^ for the B97-1 functional, when using the aug-cc-pVTZ basis set.

### C Two-Mode Couplings

Vibrational calculations that use an *n*MR representation of the quartic force field can be considered as being constructed from two distinct types of energy derivative term. These can be taken to be “diagonal” terms that involve displacement along a single normal coordinate and “mode-coupling” terms that involve displacement along more than one normal coordinate. A large part of the anharmonicity in the vibrational transitions studied here is due to anharmonic mode-coupling between modes in the nuclear PES. However, the average differences between the TAO-DFT and KS-DFT 2MR anharmonic frequencies (ΔTAO_2MR_) are smaller than the differences between the harmonic frequencies (ΔTAO_0MR_) shown in Table 5. To quantify the changes that TAO-DFT calculations make to the diagonal and mode-coupling aspects of the anaharmonicity, these differences, termed Δ_diag_ and Δ_cpl_, are defined by^59^
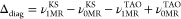
4and
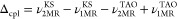
5where ν is a fundamental transition frequency in wavenumbers, the superscripts “KS” and “TAO” denote the use of the functional form in either the KS-DFT or TAO-DFT frameworks, respectively, while the *n*MR subscripts denote the truncation level of the anharmonic force field. The difference between these two types of anharmonic shift shows that the effect thermally-assisted-occupation has on the mode-coupling terms is more significant than the ΔTAO_2MR_ values alone would suggest. This is because the Δ_diag_ and Δ_cpl_ values can have the opposite sign and can partially cancel out in the combined 2MR anharmonic calculations. However, the average absolute Δ_diag_ and Δ_cpl_ values are still significantly smaller than the harmonic ΔTAO_0MR_ values, indicating that the most significant changes occur in the harmonic rather than the anharmonic force field.

**Table 5:** MADs and Mean Absolute Δ_diag_ and Δ_cpl_ Values (cm^–1^) for the KS-DFT and TAO-DFT Frequencies Shown for the Modified Anharmonic F1 Test Set Calculated with the 6-311++G(d,p) Basis Set^a^

frequency range	functional	ΔTAO_0MR_	ΔTAO_1MR_	ΔTAO_2MR_	Δ_diag_	Δ_cpl_
full	B3LYP	5.8	5.8	5.9	0.5	0.5
low	B3LYP	3.3	3.4	3.5	0.7	0.7
medium	B3LYP	4.6	4.6	4.6	0.2	0.3
high	B3LYP	15.4	15.3	15.4	0.2	0.3
full	B97-1	5.7	5.8	5.8	0.4	0.5
low	B97-1	3.1	3.2	3.3	0.7	0.8
medium	B97-1	4.5	4.6	4.5	0.2	0.3
high	B97-1	15.6	15.6	15.6	0.2	0.3

a The low, medium, and high frequency ranges correspond to 300–1000, 1000–2600, and >2600 cm^–1^, respectively.

### D Exact Exchange Fractions

Increasing the fraction of exact (HF) orbital exchange used to parametrize conventional global hybrid exchange-correlation functionals in KS-DFT can have a dramatic effect on the calculated nuclear vibrational frequencies. This has previously been shown for both scaled harmonic and anharmonic 2MR nuclear vibrations using a modified version of the BLYP functional.^63^ Anharmonic errors were found to be minimized by the inclusion of ca. 20% exact exchange when looking in increments of 10% and to linearly increase when more than 30–40% exact exchange was included. The errors associated with the calculation of mainly hydrogen stretching modes above 3000 cm^–1^ on the other hand were minimized by a higher fraction of ca. 30% exact exchange. The errors in the overall scaled harmonic frequencies were similarly minimized by the inclusion of 30–40% exact exchange, and a similar result was found using a modification of the B3LYP functional.^80^ The effects of using different fractions of exact exchange in the TAO-B3LYP framework have been investigated here for 2% increments ranging from 10 to 40%.

The general form of the KS-B3LYP exchange-correlation functional can be given by
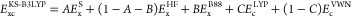
6where *A*, *B*, and *C* are empirically derived coefficients, and the superscripts refer to variations of the exchange and correlation energy functionals developed by Slater,^84^ Hartree–Fock,^85^ Becke,^86^ Vosko-Wilk-Nusair,^87^ and Lee–Yang–Parr.^88^ The amount of the exact HF exchange in the KS-B3LYP functional can therefore be modified by changing the *A* coefficient in eq 6 to introduce more or less HF exchange, while reducing or increasing the corresponding component of Slater exchange.^80^

The corresponding general form of the TAO-B3LYP exchange-correlation functional is equivalently given by^18^
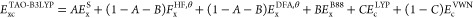
7where *E*_x_^DFA,θ^ is density functional approximation for the exchange energy at the fictitious temperature developed by Chai,^9,17,18^ and *F*_x_^HF,θ^ is the HF exchange free energy of the TAO orbitals and their occupation numbers at the given fictitious temperature (i.e., the exact exchange defined in TAO-DFT).^18^ An equivalent modification can then be made to the TAO-B3LYP functional by making the same modifications to the *A* coefficient as in eq 6, combined with a corresponding modification of the fictitious temperature in eq 2 given that *a*_*x*_ = 1 – *A* – *B*.

**Figure 3:**
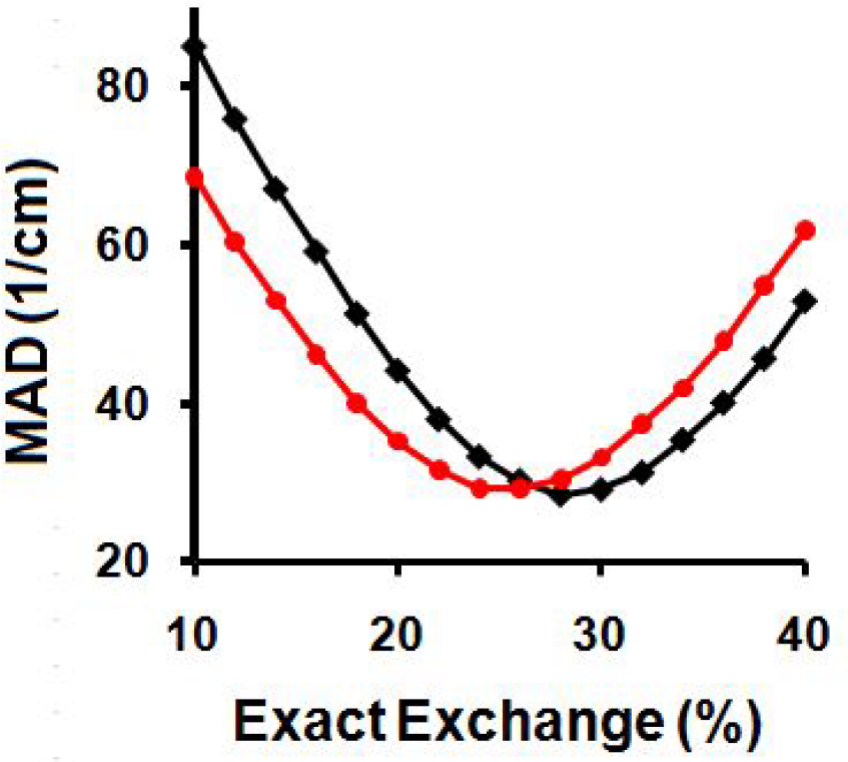
MADs from experiment for the anharmonic frequencies above 2600 cm^–1^ when calculated using modified KS-B3LYP (black line) and modified TAO-B3LYP (red line).

The results, shown in Table 6 and Figure 3 for the 6-311++G(d,p) basis set, indicate that ca. 28% HF exchange is optimal for the KS-DFT calculations of the high frequency modes, while ca. 26% HF exchange is optimal for the TAO-DFT calculations of these modes, with both methods giving MADs from experiment of ca. 29 cm^–1^ to the nearest wavenumber. The N–H stretching modes in HN_3_, HNCO, and *cyclo*-C_2_H_4_NH and stretching modes in HCOOH and H_2_O_2_ show slight increases in the TAO shifts when increasing the fraction of exact exchange before dropping for larger degrees of hybridization. The C–H stretching modes show approximately linear decreases in their TAO shifts with increasing hybridization. Overall TAO shifts are found to decrease with increasing levels of exact exchange for all of the frequency ranges studied. Decreases in TAO shifts seen for the modes below 2600 cm^–1^ are well modeled by cubic polynomial fitting functions, while the modes above 2600 cm^–1^ show an approximately linear decrease, attributed to the predominance of C–H stretching modes in this subset. TAO shifts of ca. 15.4 and 13.3 cm^–1^ are found for the high frequency modes with 20% and 28% exact exchange hybridization, respectively. This indicates that up to ca. 2 cm^–1^ of the ca. 16 cm^–1^ improvement in the experimental matching for these modes when increasing the amount of exact exchange, may be due to a corresponding decrease in the static correlation effects that are otherwise missing from the calculation when using the conventional B3LYP method with 20% HF exchange.

**Table 6:** MADs (cm^–1^) from Experiment for the Anharmonic KS-B3LYP and TAO-B3LYP Frequencies and the MADs between the Two Methods (ΔTAO), Calculated with Different Fractions of Exact Orbital Exchange^a^

	300–1000 cm^–1^			1000–2600 cm^–1^			2600+ cm^–1^		
exact exchange (%)	KS-DFT	TAO-DFT	ΔTAO	KS-DFT	TAO-DFT	ΔTAO	KS-DFT	TAO-DFT	ΔTAO
10	34.3	41.3	9.9	25.6	27.0	8.9	85.1	68.5	18.9
12	33.1	38.2	7.8	23.6	24.3	7.7	75.9	60.5	18.1
14	32.0	35.9	6.6	22.8	23.5	6.5	67.1	53.0	17.3
16	31.0	33.8	5.4	23.2	24.1	5.7	59.2	46.1	16.6
18	30.2	32.0	4.2	24.5	25.6	5.0	51.3	40.0	16.0
20	29.6	30.9	3.5	26.3	27.7	4.6	44.2	35.2	15.4
22	29.5	30.3	3.0	28.8	30.3	4.2	38.0	31.6	14.8
24	29.7	30.4	2.7	31.7	33.6	4.0	33.3	29.3	14.3
26	30.1	30.8	2.4	34.9	37.1	3.8	30.2	29.2	13.8
28	30.8	31.5	2.1	38.3	40.7	3.7	28.5	30.5	13.3
30	31.8	32.5	2.0	42.0	44.6	3.7	29.1	33.2	12.8
32	33.1	33.8	1.9	45.9	48.5	3.6	31.3	37.3	12.3
34	35.0	35.8	1.9	49.8	52.6	3.5	35.4	41.9	11.8
36	36.4	37.4	2.0	53.9	56.9	3.4	40.1	47.8	11.4
38	40.6	39.0	1.7	61.9	61.2	3.3	45.7	54.9	10.9
40	39.9	40.8	1.6	62.6	65.7	3.3	52.9	61.8	10.5

a The low, medium, and high frequency ranges correspond to 300–1000, 1000–2600, and 2600+ cm^–1^, respectively.

### E Symmetrized von Neumann Entropy

The relatively large TAO shifts seen for the high frequency modes in this study may be due to the presence of significantly more static electron correlation in these modes; however, it may alternatively be because the larger force constants and total energies that characterize these modes cause more pronounced vibrational energy changes or due to other factors.

Molecules with strong static electron correlation have more orbitals with fractional occupation numbers further from 0 or 1. Static correlation can therefore be roughly approximated using the symmetrized von Neumann entropy given by eq 8(21)
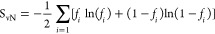
8where *f*_*i*_ is the fractional occupation numbers, and the summation runs over the total number of orbitals. Taking the second-order derivatives of this entropy term with respect to nuclear displacements along the normal mode coordinates therefore provides an approximate wave function based analysis of how the static correlation changes along these coordinates. Second-order nuclear derivatives of the entropy have been calculated numerically using a finite central difference method with a step size of 0.01 Å for the normal modes of the HCCH and ClF_3_ molecules (shown in Table 7). These molecules were chosen as they contain examples of relatively unperturbed low frequency modes, high frequency C–H stretching modes, and a selectively perturbed mode in the case of the asymmetric stretching mode of ClF_3_.

**Table 7:** Selected Second-Order Derivatives of the Symmetrized von Neumann Entropy with Respect to TAO-B97-1/aug-cc-pVTZ Normal Coordinate Nuclear Displacements, with the Corresponding Harmonic TAO-DFT Frequencies and TAO Shifts Given in cm^–1^

HCCH				ClF_3_			
mode	ω_TAO-DFT_	*S*_vN_″	ΔTAO	mode	ω_TAO-DFT_	*S*_vN_″	ΔTAO
1	653	0.037284	3	1	310	0.976417	1
2	653	0.037284	3	2	322	0.652057	0
3	766	0.027362	2	3	414	1.487041	1
4	766	0.027362	2	4	535	0.260563	2
5	2065	0.292737	6	5	706	5.715652	2
6	3418	0.008808	14	6	744	4.913759	11
7	3524	0.036502	15				

There is a weak correlation found here between the second derivatives of the symmetrized von Neumann entropy of the electronic wave function and the TAO energy shifts of the vibrational modes within the same molecule. This remains true across the low and medium frequency modes. However, the C–H stretching modes do not follow the same trend, and their entropy derivatives are not much larger than the other vibrational modes. Individual modes with larger TAO shifts for ClF_3_ show significantly larger entropy derivatives for the highest energy fluorine stretches. This correlates well with the large shifts for both these modes seen in the TAO-B3LYP/6-311++G(d,p) calculation, although the TAO-B97-1/aug-cc-pVTZ calculation used as a reference here only shows a large shift for the asymmetric stretching mode. The entropy values for ClF_3_ suggest that individual stretching modes are likely to be more strongly affected by static correlation even though the other vibrational modes of the same molecule are not affected to the same degree. The anharmonic force constants that make up the nuclear PES for the TAO-DFT and KS-DFT calculations of these two molecules have also been included to the Supporting Information.

### F Three-Mode Couplings

So far, the anharmonic transitions have been calculated with the TOSH modification of VPT2 when solving the nuclear Schrödinger equation. VPT2 is known to perform poorly in cases where vibrational modes become degenerate, or near degenerate, while the TOSH modification solves this issue.^78^ However, the TOSH method makes additional approximations over VPT2 and has only been formulated within the 2MR representation. 2MR and 3MR calculations have been carried out here using unmodified VPT2 with the B97-1/aug-cc-pVTZ KS-DFT and TAO-DFT electronic structure methods in order to examine the effects of anharmonic three-mode coupling terms. Large errors of up to ca. 302 cm^–1^ were found in excess of the TOSH values for some of the modes, namely CH_2_CHCHCH_2_ mode 20, CH_2_CHCHO mode 16, and CH_2_CH_2_ mode 9, and these modes have been removed from the test set.

The overall frequencies improve with thermally-assisted-occupation by an average of ca. 1 cm^–1^. The accuracy in the 1000–2600 cm^–1^ middle frequency ranges deteriorates for both the 2MR and 3MR calculations. However, the increase in averaged error is lower for the 3MR calculation, indicating that some of the previously observed errors are due to the 2MR VTP2 nuclear structure theory rather than the TAO-DFT electronic structure theory. The average error in the experimental matching for the transition frequencies above 2600 cm^–1^ falls from ca. 38 to 28 cm^–1^ between the 2MR and 3MR calculations using KS-DFT, indicating that the larger errors seen for the C–H stretching modes are in large part due to an increased sensitivity to three-mode coupling terms for the higher energy vibrational modes. However, the match with experiment still improves by ca. 8 cm^–1^ for the high energy modes at the 3MR level following thermally-assisted-occupation (Table 8).

**Table 8:** MAD Values in cm^–1^ from the Experiment for the VPT2 2MR and 3MR Frequencies Calculated Using KS-DFT and TAO-DFT for Different Frequency Ranges in the Anharmonic Set

method	*n*MR	all modes	300–1000 cm^–1^	1000–2600 cm^–1^	2600+ cm^–1^
KS-B97-1/aug-cc-pVTZ	2MR	25.1	22.3	23.0	38.3
KS-B97-1/aug-cc-pVTZ	3MR	20.8	17.1	22.9	27.5
TAO-B97-1/aug-cc-pVTZ	2MR	24.1	22.2	24.8	28.1
TAO-B97-1/aug-cc-pVTZ	3MR	19.5	16.7	23.3	19.9

### G Removing Multireference Molecules

Several of the molecules in the test set are known to have significant multireference character. Ozone, O_3_, is known to have a significant amount of static correlation and diradical character,^89,90^ including when displaced from equilibrium,^91^ and is often excluded from DFT vibrational benchmarking studies of harmonic scaling factors.^79,80^ Electronically excited methylene, CH_2_, is known to have multireference character,^92^ and a large TAO-DFT shift has been observed for the lowest-singlet excited state.^62^ Cl_2_O and several related species are known to have significant static correlation at their equilibrium geometries.^93^ These multireference systems are particularly affected by the use of TAO-DFT in these calculations. In addition to O_3_ and ^1^CH_2_, the molecules ClF_3_, ClNO, ClNO_2_, ClSN, Cl_2_O, SCl_2_, and H_2_S_2_ are all found to have modes that both do not contain significant amounts of hydrogen bond stretching and yet show shifts in their fundamental transitions of greater than ca. 10 cm^–1^ at the TOSH 2MR B97-1/aug-cc-pVTZ level of theory.

Many of these modes show improved matching with experiment when calculated using TAO-DFT, and these molecules predominantly contain chemical bonds involving atoms located on the second row of the periodic table, i.e., S and Cl atoms. Chlorine trifluoride, ClF_3_, for example, is a “T-shaped” molecule with two fluorine atoms around a chlorine atom in an approximately linear arrangement. This molecule is an example where not all of the vibrational coordinates are equally or significantly perturbed by thermally-assisted-occupation. ClF_3_ has six fundamental modes, and four of the transition frequencies are relatively accurate within the KS-DFT calculation, with differences from the TAO-DFT calculation of less than 2 cm^–1^. However, the highest energy transition, involving stretching of the two near linear Cl–F bonds, undergoes a large shift of ca. 13 cm^–1^, which suggests the presence of significant static correlation along this coordinate that is not present in the other coordinates. This was previously noted for the two highest energy stretching modes using the derivatives of the symmetrized von Neumann entropy along these normal coordinates. Nitrosyl chloride, ClNO, is another example of a molecule exhibiting unequal static correlation effects in different vibrational modes. In this case, the lowest two vibrational modes both contain a significant degree of Cl–N bond motion and show relatively large shifts in the TAO-DFT calculations of 19 and 23 cm^–1^. However, the highest frequency mode is localized onto the N=O bond and is only shifted by ca. 1 cm^–1^.

Care should be taken when including this set of strongly multireference molecules in regular vibrational frequency benchmarks, as the static correlation effects may cause otherwise spurious errors. Here, an analysis has been performed with these molecules removed from the test set (see Table 9), showing that the average improvements in the experimental match actually increase slightly when these molecules are removed, indicating that the large errors and improvements in the selected modes for this subset are offset by the other vibrational modes that these molecules possess. The overall experimental match is still improved by ca. 1 cm^–1^ on average, and the predictions of the high frequency modes perform slightly worse due to the removal of ^1^CH_2_ as these C–H stretching modes are particularly well treated in the TAO-DFT framework.

**Table 9:** MAD Values in cm^–1^ from the Experiment for the VPT2 2MR and 3MR Frequencies Calculated Using KS-DFT and TAO-DFT for Different Frequency Ranges in the Anharmonic Set with the Strongly Multireference Molecules Removed

method	*n*MR	all modes	300–1000 cm^–1^	1000–2600 cm^–1^	2600+ cm^–1^
KS-B97-1/aug-cc-pVTZ	2MR	24.6	23.2	20.0	38.3
KS-B97-1/aug-cc-pVTZ	3MR	20.0	17.4	20.0	27.2
TAO-B97-1/aug-cc-pVTZ	2MR	23.5	23.1	21.6	28.7
TAO-B97-1/aug-cc-pVTZ	3MR	18.6	17.0	20.1	20.3

## Conclusions

Molecular vibrational transition energies calculated using hybrid TAO-DFT differ from their KS-DFT counterparts by multiple wavenumbers for the majority of molecules in this test set, and improved matching with experimental data is often seen. The results suggest static correlation is important for determining the vibrational frequencies of several species that are regularly included in experimental benchmarking studies. In particular, the O_3_, ^1^CH_2_, ClF_3_, ClNO, ClNO_2_, ClSN, Cl_2_O, SCl_2_, and H_2_S_2_ molecules all have modes that are significantly perturbed by more than 10 cm^–1^ when using the TAO-DFT method, and for many of the species in this study, not all of the vibrational modes are significantly or equally perturbed. Larger perturbations in excess of 10 cm^–1^ are found in both the harmonic and anharmonic transitions involving hydrogen atom motion across all of these molecules, with TAO-DFT significantly improving these calculated frequencies by comparison with experimental observations. Given the improvements that can be generated using this method, hybrid TAO-DFT has potential for addressing static correlation in dynamic molecular systems, and the development of analytical second derivatives of the energy with respect to nuclear displacements is recommended.

## Supporting Information Available

Listing of calculated vibrational transition frequencies associated with molecules examined in this study (PDF)
