# Supplementary material for: Static Electron Correlation in Anharmonic Molecular Vibrations: A Hybrid TAO-DFT Study
Source: J Phys Chem A. 2022 Sep 27;126(40):7273–82. doi: 10.1021/acs.jpca.2c05881 (PMC9574917; doi:10.1021/acs.jpca.2c05881)
Supplement: Supplementary file 1 — jp2c05881_si_001.pdf [file jp2c05881_si_001.pdf]

Static Electron Correlation in Anharmonic Molecular Vibrations: A Hybrid TAO-DFT Study

Supporting Information

Magnus W. D. Hanson-Heine*

School of Chemistry, University of Nottingham, University Park, Nottingham NG7 2RD, UK.

**magnus.hansonheine@nottingham.ac.uk*

Table S1. Harmonic F38 vibrational frequencies in cm^{-1} calculated using TAO-DFT with the B3LYP and B97-1 functionals and aug-cc-pVTZ basis set at different fictitious temperatures (θ).

	B3LYP				B97-1			
Molecule	θ_0	θ_1	θ_2	θ_3	θ_0	θ_1	θ_2	θ_3
H_2	4417.70	4446.77	4449.85	4461.68	4433.72	4463.63	4466.49	4478.60
CH_4	1339.40	1343.92	1344.38	1346.07	1333.22	1337.86	1338.29	1340.03
	1557.63	1563.84	1564.46	1566.73	1554.92	1561.26	1561.84	1564.18
	3028.33	3041.75	3043.19	3048.77	3017.75	3031.57	3032.91	3038.61
	3129.44	3144.93	3146.59	3152.98	3126.47	3142.36	3143.89	3150.43
NH_3	1025.05	1029.77	1029.97	1030.08	1041.00	1045.83	1046.04	1046.29
	1663.90	1668.73	1669.16	1670.60	1663.11	1668.10	1668.51	1670.03
	3469.23	3482.04	3483.36	3488.21	3474.46	3487.64	3488.87	3493.88
	3588.25	3600.99	3602.36	3607.55	3594.60	3607.72	3608.99	3614.29
H_2O	1627.30	1631.46	1631.86	1633.22	1634.46	1638.70	1639.06	1640.46
	3796.11	3809.59	3810.93	3815.66	3829.83	3843.69	3844.94	3849.83
	3898.76	3912.06	3913.40	3918.17	3932.91	3946.57	3947.82	3952.73
HF	4072.39	4085.79	4087.18	4092.27	4118.91	4132.70	4133.99	4139.23
CO	2207.87	2212.21	2212.62	2213.87	2205.91	2210.43	2210.80	2212.08
N_2	2447.39	2452.21	2452.67	2454.16	2432.19	2437.21	2437.65	2439.14
F_2	1052.80	1051.92	1050.35	1040.50	1071.48	1071.10	1069.83	1060.85
C_2H_2	666.02	668.85	668.93	668.47	649.89	652.66	652.70	652.05
	769.47	771.83	771.96	771.84	763.22	765.59	765.67	765.39
	2068.40	2074.32	2074.67	2075.00	2058.58	2064.73	2065.03	2065.27
	3412.19	3424.70	3426.05	3431.22	3404.01	3416.99	3418.26	3423.58
	3516.92	3529.72	3531.06	3536.01	3509.74	3523.05	3524.30	3529.39
HCN	759.19	761.74	761.93	762.23	751.99	754.61	754.79	755.01
	2199.78	2205.53	2206.01	2207.24	2191.25	2197.18	2197.62	2198.79
	3444.27	3457.31	3458.69	3463.91	3435.35	3448.84	3450.13	3455.49
H_2CO	1198.03	1198.71	1197.90	1192.84	1194.13	1194.75	1193.99	1188.80
	1262.92	1265.49	1265.33	1263.76	1259.24	1261.86	1261.70	1260.11
	1530.20	1533.64	1533.70	1533.30	1526.44	1530.00	1530.06	1529.68
	1813.17	1815.48	1815.01	1811.77	1822.46	1824.82	1824.40	1821.18
	2884.64	2900.15	2901.88	2908.71	2876.66	2892.61	2894.21	2901.14
	2939.32	2956.34	2958.25	2965.80	2933.21	2950.70	2952.46	2960.12
CO_2	673.82	674.96	675.01	674.74	672.86	674.02	674.05	673.73
	1369.14	1372.34	1372.65	1373.68	1372.41	1375.68	1375.97	1376.99
	2400.35	2407.40	2408.11	2410.65	2410.66	2417.87	2418.53	2421.08
N_2O	616.79	617.63	617.50	616.03	612.85	613.67	613.54	611.94
	1323.76	1327.44	1327.68	1327.97	1323.02	1326.71	1326.91	1327.13
	2340.07	2346.72	2347.22	2348.30	2327.46	2334.29	2334.74	2335.75
Cl_2	539.19	528.19	524.51	506.56	561.61	551.64	548.57	532.08
OH	3693.29	3711.24	3712.66	3718.26	3728.58	3745.28	3746.75	3752.31

Table S2. TOSH nMR (n = 0-2) vibrational frequencies in cm^{-1} for the modified F1 anharmonic test set calculated at the KS-DFT and TAO-DFT B3LYP/6-311++G(d,p) levels of electronic structure.

	KS-DFT			TAO-DFT		
Molecule	0MR	1MR	2MR	0MR	1MR	2MR
$NClF_2$	354.72	355.36	350.61	354.76	355.38	350.32
	359.98	359.33	356.39	358.91	357.98	354.59
	561.16	559.93	552.84	561.81	560.49	553.20
	680.20	678.19	672.57	677.53	674.74	668.27
	831.34	842.45	810.22	835.71	846.69	813.99
	939.95	934.18	928.91	942.61	936.66	931.24
ClF_3	373.59	375.13	372.15	369.39	370.86	367.35
	479.91	478.55	476.67	480.71	479.31	476.86
	667.02	670.41	660.01	657.45	649.86	646.21
	679.40	672.71	670.25	663.32	666.26	654.22
HOCl	703.27	696.20	693.51	696.16	687.99	684.91
	1226.99	1310.53	1200.31	1224.88	1309.47	1197.41
	3775.27	3588.19	3549.97	3787.17	3600.02	3561.53
ClNO	325.35	322.52	321.74	300.69	296.26	295.04
	602.69	606.15	594.90	573.26	577.05	562.36
	1936.22	1901.83	1896.89	1941.08	1906.55	1902.17
$ClNO_2$	363.38	361.43	359.72	352.49	349.04	346.23
	410.78	412.15	406.39	407.92	409.18	402.39
	668.38	671.99	659.35	657.41	660.67	645.90
	808.75	809.26	801.58	804.32	804.41	795.76
	1349.54	1341.32	1333.23	1349.90	1341.40	1332.96
	1773.99	1794.14	1723.74	1776.99	1797.07	1725.91
ClSN	386.81	385.42	383.51	364.36	362.03	359.62
	1364.20	1346.27	1345.52	1356.51	1337.96	1337.09
NCl_2F	334.10	334.63	330.06	331.50	332.10	326.98
	419.30	418.34	416.40	415.57	414.36	411.96
	606.42	604.01	604.24	601.43	598.72	598.04
	646.40	649.14	638.84	639.10	641.79	630.33
	836.13	818.71	810.93	839.09	820.63	812.97
Cl_2O	618.08	621.06	608.19	573.04	577.84	560.90
	628.46	625.73	627.56	620.08	617.01	618.53
$SOCl_2$	315.51	315.26	313.93	310.78	310.19	308.58
	412.93	415.04	409.41	403.81	405.82	398.87
	455.52	454.93	453.17	448.24	447.21	444.76
	1229.80	1211.73	1209.31	1230.53	1212.13	1209.55
SCl_2	467.31	470.22	463.55	437.03	441.62	432.85
	484.51	482.84	483.08	474.70	472.71	472.73
HOF	946.19	931.51	925.74	944.75	929.02	922.93
	1396.86	1440.09	1357.23	1398.80	1442.36	1358.59
	3730.08	3544.72	3506.86	3744.70	3559.68	3521.68
ONF	497.75	493.71	494.14	497.10	492.32	492.49
	766.64	762.52	756.98	765.01	760.28	754.05
	1964.08	1930.67	1926.57	1963.91	1930.25	1926.29
NSF	341.96	342.13	338.23	342.24	342.30	338.32
	575.31	570.83	569.30	576.56	571.36	569.76
	1403.03	1384.93	1383.99	1398.65	1379.83	1378.89
F_2NH	499.33	498.18	490.57	500.77	499.58	491.89
	870.00	880.20	845.35	873.43	883.73	848.40

	985.30	980.69	974.21	988.98	984.35	977.76
	1327.44	1348.61	1291.25	1330.17	1351.46	1293.68
	1459.52	1479.73	1414.62	1463.11	1483.53	1418.04
	3358.76	3183.92	3117.83	3373.88	3199.50	3133.39
N_2F_2	365.76	365.93	359.05	365.64	365.74	358.67
	424.81	424.31	417.69	425.69	425.20	418.50
	606.53	604.09	596.60	608.27	605.68	597.83
	990.48	1000.54	964.22	995.18	1005.21	968.63
	1040.52	1032.78	1020.35	1044.02	1036.13	1023.47
	1615.52	1588.61	1582.33	1616.97	1589.65	1583.12
F_2O	474.62	473.86	468.31	473.10	472.08	466.19
	850.82	861.13	833.59	841.60	852.96	822.68
	988.04	981.96	979.27	988.03	981.68	978.47
F_2SO	324.11	322.05	318.45	325.53	323.43	319.79
	354.29	356.44	351.21	355.06	357.19	351.91
	475.93	475.17	471.03	477.76	476.97	472.75
	653.04	658.80	643.06	656.46	662.25	646.27
	722.06	719.27	715.90	725.90	723.02	719.56
	1307.43	1290.05	1286.84	1311.31	1293.87	1290.53
S_2F_2	570.85	562.71	565.90	574.04	565.66	568.62
	607.25	613.34	600.97	604.35	610.72	597.73
	640.44	636.67	637.83	640.25	636.23	637.34
HNO_3	461.85	1278.05	359.36	463.00	1282.94	360.95
	586.51	589.99	579.85	587.86	591.45	581.15
	648.83	648.42	636.09	652.14	651.85	639.47
	772.88	774.30	763.13	771.63	772.69	760.95
	896.75	897.87	881.56	900.27	901.21	884.45
	1319.95	1337.10	1286.76	1322.66	1338.92	1289.59
	1348.53	1349.91	1329.97	1350.12	1352.03	1330.73
	1755.78	1770.36	1718.44	1758.92	1773.58	1720.92
	3727.30	3544.22	3437.50	3739.35	3556.59	3449.61
HN_3	535.99	542.98	526.91	534.33	541.31	525.03
	591.43	605.63	583.20	592.51	606.19	583.68
	1174.08	1205.96	1150.05	1176.90	1209.12	1152.91
	1294.46	1291.76	1278.95	1297.34	1294.58	1281.66
	2267.21	2264.12	2214.18	2272.12	2269.18	2218.45
	3490.65	3324.05	3278.45	3503.21	3335.97	3290.17
H_2O	1602.98	1590.77	1543.52	1607.07	1594.93	1547.52
	3816.87	3728.33	3695.98	3831.88	3743.45	3711.09
	3921.99	4020.68	3715.33	3936.56	4035.20	3730.15
H_2O_2	364.15	1007.25	208.70	362.07	1018.44	202.46
	934.26	920.40	909.75	936.58	922.12	910.79
	1295.84	1331.09	1258.83	1298.26	1333.70	1260.36
	1453.02	1466.50	1424.37	1456.41	1469.89	1427.64
	3777.07	3876.78	3511.68	3791.34	3890.93	3525.38
	3777.99	3686.56	3587.33	3792.20	3700.77	3600.95
H_2S	1209.82	1206.28	1177.08	1213.66	1210.07	1180.62
	2673.20	2626.91	2608.88	2685.89	2639.19	2620.55
	2688.66	2740.90	2580.19	2699.98	2752.36	2590.15
H_2S_2	410.05	551.68	397.39	400.85	551.26	387.02
	475.30	471.74	468.26	470.26	465.92	461.64
	873.00	896.04	871.82	869.84	893.74	869.20
	877.57	897.61	877.53	875.10	895.84	875.73
	2626.44	2575.90	2531.67	2640.25	2589.04	2543.49

	2627.96	2680.69	2492.15	2641.77	2693.98	2503.99
NO_2	764.59	764.04	755.78	756.68	755.17	746.47
	1391.12	1382.92	1377.19	1388.71	1380.05	1374.12
	1701.68	1720.78	1645.55	1702.63	1721.69	1645.19
N_2O	580.72	596.70	583.46	582.74	597.99	584.46
	580.72	596.70	583.46	582.74	597.99	584.46
	1326.63	1315.86	1304.88	1330.34	1319.61	1308.56
	2343.18	2347.56	2287.64	2350.18	2354.72	2294.43
SO_2	504.41	504.30	500.28	503.25	503.02	498.87
	1131.94	1125.77	1122.44	1129.07	1122.46	1118.88
	1311.23	1319.98	1291.46	1310.93	1319.68	1290.34
O_3	746.67	744.68	736.16	729.16	726.59	717.39
	1183.37	1206.78	1155.49	1166.09	1189.83	1134.96
	1245.37	1238.68	1236.75	1220.36	1212.67	1210.14
COClF	408.76	409.30	405.48	409.82	410.36	406.49
	492.55	492.46	488.48	494.81	494.73	490.63
	667.53	671.83	659.62	667.50	671.70	659.29
	756.44	755.90	750.74	759.21	758.60	753.35
	1064.96	1060.22	1040.80	1069.46	1064.82	1045.27
	1922.54	1896.97	1890.19	1926.02	1900.34	1893.40
ClCN	391.50	422.91	386.39	392.00	423.16	386.68
	391.50	422.91	386.39	392.00	423.16	386.68
	739.62	734.26	730.68	742.79	737.31	733.66
	2309.65	2292.44	2270.98	2315.56	2298.33	2276.66
$COCl_2$	440.05	441.08	436.73	441.61	442.61	438.09
	556.75	554.08	552.46	559.55	556.68	554.68
	585.50	592.42	578.45	583.59	590.43	576.02
	819.85	825.85	806.35	824.34	830.12	810.00
	1879.11	1849.18	1846.12	1880.23	1850.04	1846.81
FCN	486.31	501.59	479.42	486.99	502.20	479.97
	486.31	501.59	479.42	486.99	502.20	479.97
	1085.73	1077.89	1070.75	1088.90	1081.04	1073.84
	2412.83	2408.66	2369.22	2420.03	2415.91	2376.26
COF_2	575.70	575.65	570.02	576.90	576.86	571.22
	615.06	616.37	610.19	616.34	617.65	611.46
	772.20	774.65	763.56	773.30	775.69	764.53
	955.61	951.33	947.52	958.96	954.70	950.88
	1201.31	1213.20	1175.01	1206.38	1218.25	1180.09
	1974.47	1954.31	1942.84	1979.83	1959.69	1948.04
CSF_2	419.33	419.82	416.23	418.80	419.26	415.55
	525.65	525.97	520.97	525.63	525.94	520.83
	619.00	620.72	612.73	611.55	612.85	604.28
	790.05	787.86	784.38	789.99	787.77	784.15
	1168.88	1181.49	1137.33	1173.72	1186.28	1142.29
	1350.46	1356.81	1334.99	1351.93	1358.36	1335.82
COS	511.34	526.00	508.55	510.86	525.25	507.49
	511.34	526.00	508.55	510.86	525.25	507.49
	876.39	871.23	866.64	879.56	874.38	869.62
	2116.71	2108.38	2078.14	2121.96	2113.79	2083.16
CO_2	668.46	677.94	664.11	669.77	679.20	665.31
	668.46	677.94	664.11	669.77	679.20	665.31
	1373.02	1367.23	1360.89	1376.48	1370.69	1364.32
	2420.15	2440.98	2370.46	2427.90	2448.68	2378.20
CS_2	397.66	403.02	395.89	393.95	398.99	391.54

	397.66	403.02	395.89	393.95	398.99	391.54
	674.19	672.32	669.78	674.69	672.78	670.09
	1554.83	1566.94	1530.32	1555.76	1567.93	1530.54
HCN	766.03	906.89	732.27	769.15	910.40	734.69
	766.03	906.89	732.27	769.15	910.40	734.69
	2195.84	2177.21	2166.28	2201.89	2183.22	2172.24
	3453.05	3364.42	3263.61	3467.07	3378.47	3277.67
HNCO	568.91	666.88	595.49	569.41	668.57	596.10
	633.18	641.36	624.35	634.71	642.78	625.70
	764.47	794.88	772.23	765.14	795.87	772.97
	1337.24	1331.64	1325.01	1340.64	1335.03	1328.36
	2339.03	2356.70	2292.05	2346.79	2364.43	2299.69
	3688.80	3538.40	3475.16	3701.80	3551.14	3487.78
H_2CO	1202.10	1216.07	1167.75	1200.04	1214.68	1165.41
	1259.55	1271.47	1239.52	1261.59	1273.66	1241.54
	1530.97	1532.83	1502.45	1534.38	1536.25	1505.82
	1814.56	1795.87	1789.35	1815.96	1797.07	1790.16
	2883.70	2818.57	2767.54	2900.79	2835.94	2784.86
	2941.45	3018.54	2746.59	2960.21	3036.96	2765.83
HCOOH	521.83	1104.22	457.26	522.78	1108.02	457.59
	659.95	665.22	661.70	661.18	666.49	662.99
	1034.47	1066.65	1008.88	1036.42	1069.14	1010.70
	1097.94	1091.51	1077.46	1102.04	1095.86	1081.84
	1268.76	1305.87	1234.80	1271.82	1308.80	1237.92
	1418.93	1431.91	1390.17	1423.47	1436.69	1394.75
	1861.26	1846.02	1829.32	1865.01	1849.70	1832.66
	2959.84	2818.63	2770.57	2976.61	2836.09	2787.86
	3798.61	3608.95	3511.29	3811.83	3621.78	3523.84
C_2Cl_2	251.61	251.61	251.61	253.32	253.32	253.32
	251.61	251.61	251.61	253.32	253.32	253.32
	473.54	472.68	472.01	474.84	473.92	473.12
	986.11	992.26	976.61	989.08	995.15	979.00
	2323.89	2315.53	2304.02	2328.92	2320.38	2308.70
C_2N_2	570.33	570.42	554.33	569.99	570.47	554.43
	570.33	570.42	554.33	569.99	570.47	554.43
	886.19	882.64	877.43	889.36	885.83	880.67
	2263.22	2274.48	2233.39	2267.29	2278.54	2237.18
	2436.44	2438.45	2408.95	2439.00	2440.60	2410.85
HCCCl	319.01	368.16	372.88	319.55	367.50	370.78
	319.02	368.16	372.88	319.55	367.50	370.78
	628.10	939.97	663.66	630.36	941.08	664.22
	628.10	939.97	663.66	630.36	941.08	664.22
	747.92	743.26	739.97	750.69	745.82	742.42
	2196.00	2187.35	2152.77	2202.06	2193.31	2158.40
	3482.30	3385.20	3270.96	3495.68	3398.61	3284.16
HCCF	426.51	430.82	400.46	427.76	431.78	400.89
	426.51	430.82	400.46	427.76	431.78	400.89
	632.52	904.36	577.45	636.03	905.51	577.64
	632.52	904.36	577.45	636.03	905.51	577.64
	1073.89	1067.37	1059.63	1076.71	1070.14	1062.30
	2319.96	2320.97	2273.38	2327.35	2328.34	2280.51
	3493.97	3399.17	3284.08	3507.39	3412.69	3297.53
HCCH	647.64	809.58	763.56	652.72	803.20	751.39
	647.64	809.58	763.56	652.72	803.20	751.39

	772.88	862.42	801.71	775.34	864.95	802.08
	772.88	862.43	801.72	775.34	864.95	802.08
	2062.01	2048.88	2027.44	2068.21	2054.99	2033.47
	3419.87	3485.45	3223.92	3433.39	3498.93	3237.44
	3522.66	3485.49	3373.68	3536.38	3499.25	3387.35
trans-CHClCHCl	348.73	348.56	346.61	349.66	349.47	347.46
	789.75	838.93	799.73	789.61	839.26	799.49
	805.28	809.97	789.40	810.32	814.92	794.16
	844.40	844.08	834.60	847.79	847.37	837.80
	928.14	974.43	926.40	929.04	975.67	927.09
	1225.29	1247.14	1211.10	1227.72	1249.75	1213.53
	1303.87	1312.74	1288.47	1306.10	1315.00	1290.70
	1643.45	1634.04	1613.75	1644.56	1634.95	1614.11
	3220.69	3288.12	3036.28	3233.74	3301.10	3049.47
	3223.78	3163.59	3090.12	3236.84	3176.79	3103.16
cis-CHClCHCl	417.89	424.90	417.12	416.13	423.14	415.31
	569.99	569.74	563.57	571.54	571.30	565.08
	703.73	700.63	695.40	708.03	704.84	699.52
	710.30	803.41	733.56	710.75	804.76	734.15
	846.77	850.13	831.68	850.42	853.76	835.20
	905.02	950.18	916.91	905.83	951.64	918.25
	1218.38	1236.95	1207.09	1219.64	1238.29	1208.28
	1318.53	1332.10	1298.59	1321.43	1335.14	1301.50
	1647.48	1637.61	1614.62	1649.19	1639.07	1615.57
	3200.29	3268.36	3009.52	3214.41	3282.38	3023.69
	3221.18	3162.01	3082.95	3234.95	3175.91	3096.60
CH_2CCl_2	301.54	301.53	299.68	301.98	301.95	300.01
	382.00	390.46	390.53	382.01	390.63	390.60
	475.43	478.60	478.03	473.75	476.96	476.24
	595.23	593.20	589.64	597.91	595.84	592.13
	688.45	783.29	714.67	688.68	784.29	714.66
	764.75	773.22	757.19	768.65	777.05	760.78
	886.73	964.62	932.35	887.27	966.76	934.16
	1092.57	1101.23	1086.81	1095.68	1104.26	1089.90
	1402.38	1405.57	1384.52	1405.55	1408.79	1387.68
	1659.32	1650.33	1628.85	1662.04	1652.85	1630.95
	3165.35	3111.56	3031.89	3178.53	3124.88	3044.93
	3260.86	3331.73	3059.77	3275.27	3346.05	3074.24
cis-CHFCHF	509.30	512.46	506.67	508.87	512.01	506.04
	770.51	770.59	763.12	771.98	772.07	764.54
	774.47	847.10	795.51	776.35	849.48	797.47
	868.73	925.06	886.02	871.98	927.84	888.59
	1009.37	1008.13	1003.25	1012.67	1011.45	1006.51
	1124.08	1131.26	1104.71	1127.69	1134.85	1108.34
	1280.16	1288.92	1267.18	1283.02	1291.81	1270.15
	1381.40	1393.37	1363.08	1384.85	1396.95	1366.68
	1755.84	1753.23	1720.92	1761.24	1758.58	1725.84
	3215.98	3283.13	3029.22	3230.26	3297.34	3043.92
	3238.78	3181.80	3103.96	3252.84	3196.04	3118.27
trans-CHFCHF	317.52	324.05	321.47	317.25	323.89	321.30
	334.97	337.52	329.77	335.22	337.76	329.85
	551.22	551.19	547.44	552.36	552.33	548.54
	824.64	863.79	826.96	826.51	865.92	828.65
	904.64	955.24	901.75	907.38	958.18	904.21

	1132.01	1129.39	1116.66	1135.51	1132.89	1120.20
	1148.78	1158.18	1125.91	1153.05	1162.40	1130.16
	1286.79	1303.40	1272.43	1289.74	1306.55	1275.53
	1303.07	1310.21	1289.73	1305.79	1313.04	1292.52
	1741.42	1738.67	1708.95	1746.35	1743.54	1713.35
	3221.89	3288.74	3038.93	3236.37	3303.11	3053.73
	3229.12	3170.49	3096.16	3243.70	3185.26	3110.86
OCHCHO	339.53	357.95	345.01	340.69	358.89	346.20
	552.03	549.95	543.97	553.78	551.79	545.86
	809.84	859.39	797.99	810.67	861.01	798.92
	1057.97	1052.50	1030.20	1058.55	1052.90	1030.02
	1071.74	1085.86	1063.89	1065.76	1079.91	1057.03
	1334.00	1342.53	1315.14	1333.37	1342.04	1314.23
	1376.12	1381.43	1356.14	1375.36	1380.83	1355.15
	1797.94	1809.70	1772.45	1794.95	1806.73	1768.76
	1800.59	1790.96	1780.53	1795.79	1785.58	1774.88
	2940.09	3011.07	2746.47	2957.23	3028.01	2763.27
	2943.33	2876.92	2805.18	2960.33	2893.85	2821.88
CH_2CH_2	834.91	875.18	844.88	836.01	876.63	846.13
	974.20	1002.04	970.09	976.74	1004.70	972.57
	976.43	999.35	970.51	979.00	1002.07	973.09
	1058.26	1071.14	1044.64	1061.89	1074.56	1047.79
	1238.15	1243.73	1226.47	1241.40	1247.05	1229.82
	1377.37	1379.87	1362.18	1381.37	1383.88	1366.13
	1471.72	1475.52	1449.00	1476.19	1480.04	1453.53
	1683.70	1679.81	1655.19	1689.51	1685.56	1660.72
	3122.03	3153.04	3000.32	3136.48	3167.46	3015.02
	3136.44	3109.49	3039.20	3150.81	3123.99	3053.72
	3193.81	3229.78	3056.22	3209.58	3245.49	3072.29
	3222.19	3257.16	3086.67	3237.41	3272.35	3102.20
cyclo-C_2H_4O	818.40	849.93	825.72	820.90	852.56	828.26
	838.98	845.09	816.55	843.77	849.77	820.97
	884.60	885.32	869.24	889.11	889.79	873.71
	1039.54	1054.97	1034.61	1043.57	1058.99	1038.48
	1140.76	1152.95	1139.97	1144.87	1156.96	1143.91
	1149.87	1166.67	1152.11	1154.63	1170.70	1155.82
	1165.14	1174.30	1156.38	1168.71	1177.91	1159.93
	1172.11	1180.26	1164.86	1175.32	1183.52	1168.14
	1298.97	1296.24	1282.60	1303.88	1301.20	1287.50
	1502.43	1505.49	1477.79	1507.08	1510.17	1482.54
	1536.98	1539.15	1511.87	1542.01	1544.19	1517.01
	3081.87	3113.79	2955.96	3096.92	3128.77	2971.15
	3088.61	3059.47	2986.46	3103.53	3074.50	3001.44
	3163.80	3200.38	3021.20	3180.18	3216.66	3037.75
	3178.82	3214.84	3038.90	3195.00	3230.93	3055.19
cyclo-C_2H_4NH	770.92	798.02	793.95	773.84	801.49	797.08
	855.37	861.32	831.33	860.36	866.26	836.44
	865.23	868.71	860.02	869.67	873.07	864.26
	915.19	946.52	930.47	917.52	948.99	932.87
	996.02	1023.74	1011.49	998.65	1026.35	1013.94
	1109.97	1125.61	1106.98	1113.62	1129.35	1110.62
	1112.86	1130.93	1112.36	1116.25	1134.31	1115.84
	1147.20	1158.06	1137.52	1151.00	1161.82	1141.43
	1234.63	1237.31	1224.05	1239.49	1242.43	1228.91

	1264.56	1280.01	1251.71	1267.68	1283.23	1254.91
	1284.49	1284.80	1274.21	1288.44	1288.62	1278.11
	1496.49	1499.37	1472.45	1501.13	1504.04	1477.07
	1524.69	1527.06	1500.34	1529.69	1532.08	1505.35
	3098.07	3131.63	2966.39	3112.72	3146.17	2981.20
	3103.32	3073.60	2997.06	3117.85	3088.24	3011.59
	3177.51	3215.47	3029.65	3193.41	3231.23	3045.80
	3190.83	3222.94	3047.99	3206.52	3238.59	3063.80
	3508.99	3340.28	3269.21	3522.40	3353.88	3282.62
CH_2CCHCl	319.47	371.78	356.53	320.33	373.02	357.48
	504.25	505.18	500.61	504.49	505.39	500.50
	569.37	624.47	606.14	567.57	624.14	604.12
	753.31	753.36	739.59	755.39	755.24	741.08
	848.90	917.75	863.46	848.71	919.06	862.15
	910.65	965.45	935.01	911.09	966.61	935.96
	1018.93	1035.01	1015.91	1020.25	1036.46	1017.17
	1132.76	1133.97	1121.01	1134.89	1136.14	1122.86
	1277.47	1298.97	1256.41	1280.35	1301.92	1259.03
	1463.20	1466.96	1445.65	1466.88	1470.70	1449.30
	2050.27	2064.59	2008.96	2054.04	2068.18	2011.54
	3113.07	3056.45	2977.78	3127.18	3070.68	2991.49
	3187.40	3259.77	2980.45	3202.84	3275.06	2995.64
	3201.83	3072.51	3006.82	3215.25	3086.19	3020.29
CH_2CHCHO	321.81	343.12	343.85	321.44	342.97	343.74
	572.38	572.71	572.35	573.23	573.61	573.28
	609.96	663.83	629.47	610.91	665.38	630.34
	921.09	938.49	926.01	924.02	941.67	929.18
	997.35	1036.75	1004.34	996.81	1036.74	1003.67
	1024.35	1042.29	1022.11	1024.75	1041.55	1021.68
	1033.01	1046.76	1030.77	1033.50	1047.99	1031.28
	1170.84	1170.89	1155.45	1173.60	1173.67	1158.04
	1297.36	1302.97	1285.08	1299.98	1305.48	1287.77
	1386.83	1393.79	1367.16	1388.62	1395.86	1368.58
	1453.43	1455.82	1438.01	1456.10	1458.50	1440.65
	1673.83	1670.14	1646.00	1675.31	1671.47	1646.79
	1770.03	1753.31	1741.05	1767.57	1750.03	1737.53
	2881.25	2736.07	2683.77	2897.03	2752.16	2699.57
	3130.23	3073.30	2992.15	3144.15	3087.09	3005.90
	3170.13	3063.55	2985.73	3185.11	3078.05	3000.57
	3222.13	3258.98	3032.88	3237.51	3274.12	3048.13
cyclo-C_3H_6	743.12	776.99	774.68	745.18	779.13	776.84
	744.20	775.93	769.73	746.20	778.16	772.21
	860.59	884.83	880.68	862.13	886.55	882.48
	876.40	877.83	857.10	881.68	882.80	862.65
	877.25	878.12	857.11	882.51	883.64	862.46
	1047.03	1069.19	1058.45	1050.51	1072.67	1061.84
	1048.94	1068.54	1052.97	1052.39	1072.05	1056.50
	1085.95	1096.97	1090.84	1088.95	1100.10	1094.13
	1147.60	1153.67	1139.17	1151.64	1157.77	1143.42
	1208.79	1212.56	1200.45	1212.25	1216.06	1204.08
	1208.97	1212.70	1200.26	1212.43	1216.19	1203.85
	1211.28	1207.79	1196.94	1216.52	1213.08	1202.26
	1471.18	1474.59	1452.67	1475.78	1479.24	1457.35
	1472.04	1474.95	1451.16	1476.63	1479.60	1455.90

	1518.54	1520.15	1498.10	1523.73	1525.34	1503.36
	3119.92	3151.73	2991.10	3134.65	3166.38	3005.94
	3120.16	3133.54	2999.69	3134.88	3148.33	3014.48
	3127.88	3108.79	3030.94	3142.33	3123.31	3045.39
	3195.39	3231.62	3050.63	3211.33	3247.48	3066.70
	3195.56	3231.61	3051.11	3211.49	3247.47	3067.17
	3217.11	3240.70	3093.49	3232.54	3256.09	3108.95
$CH_2CHCHCH_2$	518.07	520.99	525.00	518.40	521.43	525.64
	536.36	594.90	592.07	537.07	596.36	593.59
	777.85	791.53	799.25	777.43	791.44	799.01
	898.40	905.98	900.90	901.79	909.49	904.69
	936.10	964.56	961.69	936.81	965.58	962.73
	937.12	963.68	964.37	937.51	964.55	965.37
	996.74	1017.06	1003.44	998.64	1018.85	1004.31
	1003.48	1015.40	1010.11	1005.09	1017.13	1012.01
	1051.57	1068.37	1054.82	1054.00	1070.89	1056.61
	1226.26	1225.65	1210.74	1229.83	1229.23	1214.42
	1313.19	1315.39	1300.37	1315.36	1317.43	1302.52
	1319.68	1325.16	1304.69	1322.05	1327.59	1307.27
	1413.66	1415.43	1396.66	1417.00	1418.80	1400.01
	1472.69	1474.63	1452.23	1476.67	1478.65	1456.39
	1645.72	1651.12	1619.89	1648.83	1654.21	1622.41
	1699.34	1698.52	1671.50	1698.48	1697.36	1669.54
	3122.76	3111.39	2993.70	3137.17	3124.69	3008.09
	3131.66	3155.94	3012.91	3145.68	3169.45	3027.79
	3134.68	3113.83	3031.95	3148.55	3127.73	3045.78
	3135.96	3183.44	2980.95	3150.28	3196.34	2997.51
	3219.88	3244.34	3077.93	3235.33	3259.99	3093.60
	3220.27	3257.18	3074.14	3235.65	3272.62	3089.67
$^1CH_2$	1385.52	1374.54	1344.63	1309.25	1290.23	1262.45
	2894.93	2831.87	2807.76	2926.47	2862.40	2838.33
	2960.44	3030.41	2810.18	3012.87	3082.47	2861.46
$^3CH_2$	1040.16	992.20	940.62	1045.59	997.94	945.87
	3118.17	3057.28	3034.06	3133.57	3072.80	3049.46
	3362.27	3431.60	3219.35	3378.08	3447.33	3235.37
HCO	1099.82	1115.02	1067.10	1093.88	1109.59	1060.71
	1934.38	1907.92	1909.24	1938.16	1911.73	1912.87
	2652.36	2414.66	2387.11	2680.39	2445.31	2418.11

Table S3. TOSH nMR (n = 0-2) vibrational frequencies in cm^{-1} for the modified F1 anharmonic test set calculated at the KS-DFT and TAO-DFT B97-1/6-311++G(d,p) levels of electronic structure.

	KS-DFT			TAO-DFT		
Molecule	0MR	1MR	2MR	0MR	1MR	2MR
$NClF_2$	357.88	355.36	350.61	357.92	355.38	350.32
	369.36	359.33	356.39	368.55	357.98	354.59
	570.65	559.93	552.84	571.33	560.49	553.20
	697.39	678.19	672.57	694.91	674.74	668.27
	858.76	842.45	810.22	863.16	846.69	813.99
	961.62	934.18	928.91	964.33	936.66	931.24
ClF_3	383.54	375.13	372.15	379.81	370.86	367.35
	491.71	478.55	476.67	492.74	479.31	476.86
	679.72	670.41	660.01	673.53	649.86	646.21
	692.91	672.71	670.25	676.90	666.26	654.22
HOCl	721.80	696.20	693.51	715.56	687.99	684.91
	1232.61	1310.53	1200.31	1230.69	1309.47	1197.41
	3809.05	3588.19	3549.97	3821.28	3600.02	3561.53
ClNO	334.39	322.52	321.74	313.42	296.26	295.04
	614.75	606.15	594.90	589.08	577.05	562.36
	1928.76	1901.83	1896.89	1932.37	1906.55	1902.17
$ClNO_2$	380.09	361.43	359.72	371.44	349.04	346.23
	417.86	412.15	406.39	415.58	409.18	402.39
	673.86	671.99	659.35	663.78	660.67	645.90
	811.86	809.26	801.58	807.90	804.41	795.76
	1353.13	1341.32	1333.23	1353.27	1341.40	1332.96
	1776.30	1794.14	1723.74	1778.83	1797.07	1725.91
ClSN	401.23	385.42	383.51	381.46	362.03	359.62
	1364.02	1346.27	1345.52	1355.78	1337.96	1337.09
NCl_2F	341.97	334.63	330.06	339.89	332.10	326.98
	427.95	418.34	416.40	424.50	414.36	411.96
	622.83	604.01	604.24	618.21	598.72	598.04
	671.17	649.14	638.84	664.50	641.79	630.33
	863.92	818.71	810.93	866.96	820.63	812.97
Cl_2O	642.87	621.06	608.19	601.54	577.84	560.90
	643.79	625.73	627.56	636.26	617.01	618.53
$SOCl_2$	326.44	315.26	313.93	322.54	310.19	308.58
	430.91	415.04	409.41	422.93	405.82	398.87
	470.29	454.93	453.17	464.03	447.21	444.76
	1248.95	1211.73	1209.31	1249.90	1212.13	1209.55
SCl_2	488.21	470.22	463.55	460.65	441.62	432.85
	500.99	482.84	483.08	492.26	472.71	472.73
HOF	968.52	931.51	925.74	967.93	929.02	922.93
	1411.90	1440.09	1357.23	1414.08	1442.36	1358.59
	3765.41	3544.72	3506.86	3780.29	3559.68	3521.68
ONF	512.00	493.71	494.14	512.17	492.32	492.49
	782.43	762.52	756.98	781.51	760.28	754.05
	1950.33	1930.67	1926.57	1950.08	1930.25	1926.29
NSF	345.80	342.13	338.23	346.06	342.30	338.32
	582.84	570.83	569.30	584.08	571.36	569.76
	1403.24	1384.93	1383.99	1398.69	1379.83	1378.89
F_2NH	506.51	498.18	490.57	507.94	499.58	491.89
	893.98	880.20	845.35	897.49	883.73	848.40

	1004.03	980.69	974.21	1007.76	984.35	977.76
	1336.53	1348.61	1291.25	1339.32	1351.46	1293.68
	1466.34	1479.73	1414.62	1469.95	1483.53	1418.04
	3368.17	3183.92	3117.83	3383.56	3199.50	3133.39
N_2F_2	368.60	365.93	359.05	368.48	365.74	358.67
	427.05	424.31	417.69	427.89	425.20	418.50
	616.73	604.09	596.60	618.44	605.68	597.83
	1014.81	1000.54	964.22	1019.52	1005.21	968.63
	1061.08	1032.78	1020.35	1064.64	1036.13	1023.47
	1604.74	1588.61	1582.33	1606.21	1589.65	1583.12
F_2O	485.69	473.86	468.31	484.52	472.08	466.19
	875.74	861.13	833.59	868.05	852.96	822.68
	1009.49	981.96	979.27	1010.01	981.68	978.47
F_2SO	330.95	322.05	318.45	332.39	323.43	319.79
	359.21	356.44	351.21	359.95	357.19	351.91
	483.26	475.17	471.03	485.12	476.97	472.75
	657.85	658.80	643.06	661.29	662.25	646.27
	727.65	719.27	715.90	731.52	723.02	719.56
	1329.73	1290.05	1286.84	1333.63	1293.87	1290.53
S_2F_2	569.73	562.71	565.90	572.44	565.66	568.62
	616.37	613.34	600.97	613.35	610.72	597.73
	650.01	636.67	637.83	649.85	636.23	637.34
HNO_3	468.53	1278.05	359.36	469.79	1282.94	360.95
	591.50	589.99	579.85	592.69	591.45	581.15
	663.12	648.42	636.09	665.91	651.85	639.47
	775.34	774.30	763.13	773.98	772.69	760.95
	915.42	897.87	881.56	919.28	901.21	884.45
	1327.97	1337.10	1286.76	1330.78	1338.92	1289.59
	1358.04	1349.91	1329.97	1359.71	1352.03	1330.73
	1765.06	1770.36	1718.44	1768.32	1773.58	1720.92
	3759.86	3544.22	3437.50	3772.15	3556.59	3449.61
HN_3	531.30	542.98	526.91	529.44	541.31	525.03
	585.95	605.63	583.20	587.03	606.19	583.68
	1178.95	1205.96	1150.05	1181.76	1209.12	1152.91
	1291.51	1291.76	1278.95	1294.36	1294.58	1281.66
	2256.47	2264.12	2214.18	2261.42	2269.18	2218.45
	3499.55	3324.05	3278.45	3512.34	3335.97	3290.17
H_2O	1611.73	1590.77	1543.52	1615.89	1594.93	1547.52
	3850.66	3728.33	3695.98	3865.93	3743.45	3711.09
	3957.03	4020.68	3715.33	3971.86	4035.20	3730.15
H_2O_2	374.37	1007.25	208.70	372.72	1018.44	202.46
	961.71	920.40	909.75	964.59	922.12	910.79
	1311.13	1331.09	1258.83	1313.72	1333.70	1260.36
	1463.24	1466.50	1424.37	1466.64	1469.89	1427.64
	3809.30	3876.78	3511.68	3823.86	3890.93	3525.38
	3809.81	3686.56	3587.33	3824.32	3700.77	3600.95
H_2S	1203.88	1206.28	1177.08	1207.86	1210.07	1180.62
	2663.66	2626.91	2608.88	2676.12	2639.19	2620.55
	2680.28	2740.90	2580.19	2691.30	2752.36	2590.15
H_2S_2	416.81	551.68	397.39	407.70	551.26	387.02
	492.45	471.74	468.26	487.72	465.92	461.64
	871.87	896.04	871.82	868.82	893.74	869.20
	877.36	897.61	877.53	875.04	895.84	875.73
	2613.27	2575.90	2531.67	2627.15	2589.04	2543.49

	2614.67	2680.69	2492.15	2628.57	2693.98	2503.99
NO_2	764.73	764.04	755.78	756.59	755.17	746.47
	1396.83	1382.92	1377.19	1394.24	1380.05	1374.12
	1706.65	1720.78	1645.55	1707.34	1721.69	1645.19
N_2O	575.58	596.70	583.46	577.61	597.99	584.46
	575.58	596.70	583.46	577.61	597.99	584.46
	1328.75	1315.86	1304.88	1332.42	1319.61	1308.56
	2334.11	2347.56	2287.64	2341.22	2354.72	2294.43
SO_2	508.69	504.30	500.28	507.57	503.02	498.87
	1150.20	1125.77	1122.44	1147.62	1122.46	1118.88
	1334.51	1319.98	1291.46	1334.55	1319.68	1290.34
O_3	753.74	744.68	736.16	736.92	726.59	717.39
	1229.54	1206.78	1155.49	1213.50	1189.83	1134.96
	1272.50	1238.68	1236.75	1248.79	1212.67	1210.14
COClF	409.15	409.30	405.48	410.19	410.36	406.49
	495.93	492.46	488.48	498.03	494.73	490.63
	668.32	671.83	659.62	668.18	671.70	659.29
	763.32	755.90	750.74	765.98	758.60	753.35
	1085.25	1060.22	1040.80	1089.77	1064.82	1045.27
	1935.09	1896.97	1890.19	1938.62	1900.34	1893.40
ClCN	386.32	422.91	386.39	386.73	423.16	386.68
	386.32	422.91	386.39	386.73	423.16	386.68
	739.70	734.26	730.68	742.56	737.31	733.66
	2303.73	2292.44	2270.98	2309.64	2298.33	2276.66
$COCl_2$	443.28	441.08	436.73	444.62	442.61	438.09
	566.22	554.08	552.46	568.67	556.68	554.68
	583.71	592.42	578.45	581.56	590.43	576.02
	836.27	825.85	806.35	840.32	830.12	810.00
	1895.29	1849.18	1846.12	1896.45	1850.04	1846.81
FCN	482.72	501.59	479.42	483.35	502.20	479.97
	482.72	501.59	479.42	483.35	502.20	479.97
	1094.16	1077.89	1070.75	1097.42	1081.04	1073.84
	2407.43	2408.66	2369.22	2414.85	2415.91	2376.26
COF_2	580.49	575.65	570.02	581.69	576.86	571.22
	617.75	616.37	610.19	619.05	617.65	611.46
	776.27	774.65	763.56	777.38	775.69	764.53
	966.16	951.33	947.52	969.57	954.70	950.88
	1221.80	1213.20	1175.01	1226.99	1218.25	1180.09
	1984.47	1954.31	1942.84	1989.97	1959.69	1948.04
CSF_2	420.06	419.82	416.23	419.53	419.26	415.55
	528.78	525.97	520.97	528.76	525.94	520.83
	623.49	620.72	612.73	615.97	612.85	604.28
	795.26	787.86	784.38	795.09	787.77	784.15
	1194.62	1181.49	1137.33	1199.41	1186.28	1142.29
	1361.32	1356.81	1334.99	1362.93	1358.36	1335.82
COS	509.76	526.00	508.55	509.22	525.25	507.49
	509.76	526.00	508.55	509.22	525.25	507.49
	871.62	871.23	866.64	875.10	874.38	869.62
	2128.67	2108.38	2078.14	2134.03	2113.79	2083.16
CO_2	667.16	677.94	664.11	668.47	679.20	665.31
	667.16	677.94	664.11	668.47	679.20	665.31
	1377.03	1367.23	1360.89	1380.55	1370.69	1364.32
	2432.07	2440.98	2370.46	2439.94	2448.68	2378.20
CS_2	395.47	403.02	395.89	391.66	398.99	391.54

	395.47	403.02	395.89	391.66	398.99	391.54
	670.12	672.32	669.78	670.75	672.78	670.09
	1550.66	1566.94	1530.32	1551.91	1567.93	1530.54
HCN	758.19	906.89	732.27	761.36	910.40	734.69
	758.19	906.89	732.27	761.36	910.40	734.69
	2188.35	2177.21	2166.28	2194.51	2183.22	2172.24
	3448.99	3364.42	3263.61	3463.32	3378.47	3277.67
HNCO	566.48	666.88	595.49	566.98	668.57	596.10
	629.93	641.36	624.35	631.48	642.78	625.70
	774.03	794.88	772.23	774.64	795.87	772.97
	1334.28	1331.64	1325.01	1337.77	1335.03	1328.36
	2339.15	2356.70	2292.05	2347.03	2364.43	2299.69
	3696.90	3538.40	3475.16	3710.20	3551.14	3487.78
H_2CO	1199.68	1216.07	1167.75	1197.66	1214.68	1165.41
	1257.74	1271.47	1239.52	1259.87	1273.66	1241.54
	1529.49	1532.83	1502.45	1533.06	1536.25	1505.82
	1825.74	1795.87	1789.35	1827.24	1797.07	1790.16
	2881.45	2818.57	2767.54	2898.82	2835.94	2784.86
	2941.15	3018.54	2746.59	2960.22	3036.96	2765.83
HCOOH	524.86	1104.22	457.26	525.79	1108.02	457.59
	661.27	665.22	661.70	662.50	666.49	662.99
	1034.51	1066.65	1008.88	1036.49	1069.14	1010.70
	1109.14	1091.51	1077.46	1113.24	1095.86	1081.84
	1275.76	1305.87	1234.80	1278.88	1308.80	1237.92
	1418.99	1431.91	1390.17	1423.67	1436.69	1394.75
	1872.87	1846.02	1829.32	1876.73	1849.70	1832.66
	2957.11	2818.63	2770.57	2974.28	2836.09	2787.86
	3832.63	3608.95	3511.29	3846.22	3621.78	3523.84
C_2Cl_2	230.21	251.61	251.61	232.12	253.32	253.32
	230.21	251.61	251.61	232.12	253.32	253.32
	474.67	472.68	472.01	475.69	473.92	473.12
	991.01	992.26	976.61	993.31	995.15	979.00
	2317.61	2315.53	2304.02	2322.44	2320.38	2308.70
C_2N_2	564.47	570.42	554.33	564.02	570.47	554.43
	564.47	570.42	554.33	564.02	570.47	554.43
	882.53	882.64	877.43	885.85	885.83	880.67
	2255.84	2274.48	2233.39	2259.91	2278.54	2237.18
	2430.99	2438.45	2408.95	2433.40	2440.60	2410.85
HCCCl	311.50	368.16	372.88	311.96	367.50	370.78
	311.50	368.16	372.88	311.96	367.50	370.78
	618.43	939.97	663.66	620.72	941.08	664.22
	618.43	939.97	663.66	620.72	941.08	664.22
	750.12	743.26	739.97	752.57	745.82	742.42
	2188.21	2187.35	2152.77	2194.25	2193.31	2158.40
	3480.85	3385.20	3270.96	3494.55	3398.61	3284.16
HCCF	421.06	430.82	400.46	422.29	431.78	400.89
	421.06	430.82	400.46	422.29	431.78	400.89
	619.54	904.36	577.45	622.93	905.51	577.64
	619.54	904.36	577.45	622.93	905.51	577.64
	1083.38	1067.37	1059.63	1086.26	1070.14	1062.30
	2314.94	2320.97	2273.38	2322.50	2328.34	2280.51
	3493.30	3399.17	3284.08	3507.03	3412.69	3297.53
HCCH	638.72	809.58	763.56	643.40	803.20	751.39
	638.72	809.58	763.56	643.40	803.20	751.39

	769.53	862.42	801.71	771.87	864.95	802.08
	769.53	862.43	801.72	771.87	864.95	802.08
	2052.05	2048.88	2027.44	2058.39	2054.99	2033.47
	3417.74	3485.45	3223.92	3431.61	3498.93	3237.44
	3521.05	3485.49	3373.68	3535.13	3499.25	3387.35
trans-CHClCHCl	349.54	348.56	346.61	350.39	349.47	347.46
	779.15	838.93	799.73	778.84	839.26	799.49
	816.32	809.97	789.40	821.27	814.92	794.16
	848.41	844.08	834.60	851.72	847.37	837.80
	924.24	974.43	926.40	924.93	975.67	927.09
	1216.84	1247.14	1211.10	1219.29	1249.75	1213.53
	1297.23	1312.74	1288.47	1299.46	1315.00	1290.70
	1637.29	1634.04	1613.75	1638.24	1634.95	1614.11
	3217.22	3288.12	3036.28	3230.60	3301.10	3049.47
	3220.88	3163.59	3090.12	3234.27	3176.79	3103.16
cis-CHClCHCl	414.81	424.90	417.12	412.82	423.14	415.31
	570.21	569.74	563.57	571.62	571.30	565.08
	707.78	700.63	695.40	708.04	704.84	699.52
	711.61	803.41	733.56	715.93	804.76	734.15
	852.23	850.13	831.68	855.88	853.76	835.20
	895.53	950.18	916.91	896.07	951.64	918.25
	1210.02	1236.95	1207.09	1211.28	1238.29	1208.28
	1311.20	1332.10	1298.59	1314.13	1335.14	1301.50
	1642.03	1637.61	1614.62	1643.53	1639.07	1615.57
	3197.76	3268.36	3009.52	3212.25	3282.38	3023.69
	3218.98	3162.01	3082.95	3233.13	3175.91	3096.60
CH_2CCl_2	300.62	301.53	299.68	301.00	301.95	300.01
	378.82	390.46	390.53	378.76	390.63	390.60
	472.19	478.60	478.03	470.32	476.96	476.24
	600.65	593.20	589.64	603.30	595.84	592.13
	686.27	783.29	714.67	686.37	784.29	714.66
	774.85	773.22	757.19	778.51	777.05	760.78
	877.70	964.62	932.35	878.06	966.76	934.16
	1091.62	1101.23	1086.81	1094.83	1104.26	1089.90
	1396.33	1405.57	1384.52	1399.57	1408.79	1387.68
	1653.00	1650.33	1628.85	1655.66	1652.85	1630.95
	3161.32	3111.56	3031.89	3174.82	3124.88	3044.93
	3260.97	3331.73	3059.77	3275.71	3346.05	3074.24
cis-CHFCHF	510.23	512.46	506.67	509.68	512.01	506.04
	773.86	770.59	763.12	775.72	772.07	764.54
	774.24	847.10	795.51	775.72	849.48	797.47
	860.14	925.06	886.02	863.38	927.84	888.59
	1015.73	1008.13	1003.25	1019.09	1011.45	1006.51
	1136.76	1131.26	1104.71	1140.48	1134.85	1108.34
	1282.25	1288.92	1267.18	1285.26	1291.81	1270.15
	1383.47	1393.37	1363.08	1387.07	1396.95	1366.68
	1753.04	1753.23	1720.92	1758.53	1758.58	1725.84
	3212.00	3283.13	3029.22	3226.69	3297.34	3043.92
	3235.23	3181.80	3103.96	3249.68	3196.04	3118.27
trans-CHFCHF	317.05	324.05	321.47	316.76	323.89	321.30
	336.49	337.52	329.77	336.70	337.76	329.85
	553.71	551.19	547.44	554.88	552.33	548.54
	815.92	863.79	826.96	817.74	865.92	828.65
	904.31	955.24	901.75	906.97	958.18	904.21

	1142.68	1129.39	1116.66	1146.31	1132.89	1120.20
	1163.29	1158.18	1125.91	1167.70	1162.40	1130.16
	1287.23	1303.40	1272.43	1290.33	1306.55	1275.53
	1301.54	1310.21	1289.73	1304.34	1313.04	1292.52
	1737.42	1738.67	1708.95	1742.48	1743.54	1713.35
	3217.19	3288.74	3038.93	3232.04	3303.11	3053.73
	3224.59	3170.49	3096.16	3239.54	3185.26	3110.86
OCHCHO	334.93	357.95	345.01	336.16	358.89	346.20
	549.85	549.95	543.97	551.63	551.79	545.86
	804.70	859.39	797.99	805.59	861.01	798.92
	1055.46	1052.50	1030.20	1055.98	1052.90	1030.02
	1067.83	1085.86	1063.89	1061.64	1079.91	1057.03
	1328.56	1342.53	1315.14	1327.94	1342.04	1314.23
	1373.13	1381.43	1356.14	1372.32	1380.83	1355.15
	1809.34	1809.70	1772.45	1806.50	1806.73	1768.76
	1813.48	1790.96	1780.53	1808.88	1785.58	1774.88
	2936.45	3011.07	2746.47	2953.94	3028.01	2763.27
	2940.00	2876.92	2805.18	2957.34	2893.85	2821.88
CH_2CH_2	828.23	875.18	844.88	829.36	876.63	846.13
	967.94	1002.04	970.09	970.30	1004.70	972.57
	971.44	999.35	970.51	973.91	1002.07	973.09
	1056.13	1071.14	1044.64	1059.65	1074.56	1047.79
	1233.81	1243.73	1226.47	1237.15	1247.05	1229.82
	1373.19	1379.87	1362.18	1377.27	1383.88	1366.13
	1464.82	1475.52	1449.00	1469.41	1480.04	1453.53
	1676.59	1679.81	1655.19	1682.44	1685.56	1660.72
	3118.70	3153.04	3000.32	3133.53	3167.46	3015.02
	3133.93	3109.49	3039.20	3148.67	3123.99	3053.72
	3194.35	3229.78	3056.22	3210.52	3245.49	3072.29
	3222.64	3257.16	3086.67	3238.27	3272.35	3102.20
cyclo-C_2H_4O	813.13	849.93	825.72	815.71	852.56	828.26
	834.64	845.09	816.55	839.51	849.77	820.97
	872.47	885.32	869.24	877.11	889.79	873.71
	1038.53	1054.97	1034.61	1042.67	1058.99	1038.48
	1136.71	1152.95	1139.97	1140.96	1156.96	1143.91
	1142.78	1166.67	1152.11	1147.62	1170.70	1155.82
	1164.99	1174.30	1156.38	1168.64	1177.91	1159.93
	1165.83	1180.26	1164.86	1169.12	1183.52	1168.14
	1296.30	1296.24	1282.60	1301.31	1301.20	1287.50
	1497.32	1505.49	1477.79	1502.10	1510.17	1482.54
	1532.45	1539.15	1511.87	1537.62	1544.19	1517.01
	3077.31	3113.79	2955.96	3092.70	3128.77	2971.15
	3084.57	3059.47	2986.46	3099.82	3074.50	3001.44
	3162.65	3200.38	3021.20	3179.38	3216.66	3037.75
	3177.66	3214.84	3038.90	3194.19	3230.93	3055.19
cyclo-C_2H_4NH	762.87	798.02	793.95	766.01	801.49	797.08
	843.87	861.32	831.33	848.86	866.26	836.44
	849.54	868.71	860.02	853.94	873.07	864.26
	910.58	946.52	930.47	912.99	948.99	932.87
	992.47	1023.74	1011.49	995.15	1026.35	1013.94
	1103.33	1125.61	1106.98	1106.80	1129.35	1110.62
	1103.79	1130.93	1112.36	1107.50	1134.31	1115.84
	1142.06	1158.06	1137.52	1145.96	1161.82	1141.43
	1228.29	1237.31	1224.05	1233.26	1242.43	1228.91

	1260.38	1280.01	1251.71	1263.58	1283.23	1254.91
	1282.13	1284.80	1274.21	1286.14	1288.62	1278.11
	1489.69	1499.37	1472.45	1494.46	1504.04	1477.07
	1518.03	1527.06	1500.34	1523.16	1532.08	1505.35
	3095.53	3131.63	2966.39	3110.50	3146.17	2981.20
	3101.17	3073.60	2997.06	3116.03	3088.24	3011.59
	3179.23	3215.47	3029.65	3195.45	3231.23	3045.80
	3192.53	3222.94	3047.99	3208.54	3238.59	3063.80
	3518.46	3340.28	3269.21	3532.12	3353.88	3282.62
CH_2CCHCl	315.78	371.78	356.53	316.66	373.02	357.48
	505.10	505.18	500.61	505.16	505.39	500.50
	564.41	624.47	606.14	562.44	624.14	604.12
	761.16	753.36	739.59	763.06	755.24	741.08
	842.22	917.75	863.46	841.79	919.06	862.15
	902.48	965.45	935.01	902.70	966.61	935.96
	1013.65	1035.01	1015.91	1014.96	1036.46	1017.17
	1127.81	1133.97	1121.01	1129.92	1136.14	1122.86
	1271.68	1298.97	1256.41	1274.53	1301.92	1259.03
	1456.42	1466.96	1445.65	1460.15	1470.70	1449.30
	2042.11	2064.59	2008.96	2045.83	2068.18	2011.54
	3111.83	3056.45	2977.78	3126.20	3070.68	2991.49
	3190.65	3259.77	2980.45	3206.37	3275.06	2995.64
	3200.75	3072.51	3006.82	3214.49	3086.19	3020.29
CH_2CHCHO	317.90	343.12	343.85	317.47	342.97	343.74
	569.75	572.71	572.35	570.62	573.61	573.28
	606.41	663.83	629.47	607.37	665.38	630.34
	915.71	938.49	926.01	918.71	941.67	929.18
	992.86	1036.75	1004.34	992.11	1036.74	1003.67
	1018.10	1042.29	1022.11	1018.41	1041.55	1021.68
	1030.54	1046.76	1030.77	1030.88	1047.99	1031.28
	1165.24	1170.89	1155.45	1168.04	1173.67	1158.04
	1291.69	1302.97	1285.08	1294.38	1305.48	1287.77
	1381.32	1393.79	1367.16	1383.15	1395.86	1368.58
	1448.04	1455.82	1438.01	1450.73	1458.50	1440.65
	1667.30	1670.14	1646.00	1668.73	1671.47	1646.79
	1782.28	1753.31	1741.05	1779.89	1750.03	1737.53
	2877.97	2736.07	2683.77	2894.04	2752.16	2699.57
	3125.35	3073.30	2992.15	3139.63	3087.09	3005.90
	3168.53	3063.55	2985.73	3183.93	3078.05	3000.57
	3220.97	3258.98	3032.88	3236.73	3274.12	3048.13
cyclo-C_3H_6	736.67	776.99	774.68	738.81	779.13	776.84
	738.27	775.93	769.73	740.34	778.16	772.21
	850.88	884.83	880.68	852.46	886.55	882.48
	854.67	877.83	857.10	860.05	882.80	862.65
	855.27	878.12	857.11	860.64	883.64	862.46
	1038.98	1069.19	1058.45	1042.50	1072.67	1061.84
	1041.31	1068.54	1052.97	1044.77	1072.05	1056.50
	1073.46	1096.97	1090.84	1076.53	1100.10	1094.13
	1142.80	1153.67	1139.17	1146.93	1157.77	1143.42
	1201.73	1212.56	1200.45	1205.29	1216.06	1204.08
	1201.98	1212.70	1200.26	1205.54	1216.19	1203.85
	1202.37	1207.79	1196.94	1207.64	1213.08	1202.26
	1463.06	1474.59	1452.67	1467.79	1479.24	1457.35
	1464.12	1474.95	1451.16	1468.84	1479.60	1455.90

	1510.11	1520.15	1498.10	1515.40	1525.34	1503.36
	3116.73	3151.73	2991.10	3131.80	3166.38	3005.94
	3116.90	3133.54	2999.69	3131.94	3148.33	3014.48
	3125.07	3108.79	3030.94	3139.85	3123.31	3045.39
	3196.43	3231.62	3050.63	3212.71	3247.48	3066.70
	3196.54	3231.61	3051.11	3212.80	3247.47	3067.17
	3217.93	3240.70	3093.49	3233.67	3256.09	3108.95
$CH_2CHCHCH_2$	513.81	520.99	525.00	514.12	521.43	525.64
	532.38	594.90	592.07	533.03	596.36	593.59
	774.61	791.53	799.25	773.99	791.44	799.01
	894.41	905.98	900.90	897.88	909.49	904.69
	930.74	964.56	961.69	930.90	965.58	962.73
	930.77	963.68	964.37	931.15	964.55	965.37
	990.72	1017.06	1003.44	992.44	1018.85	1004.31
	996.58	1015.40	1010.11	998.24	1017.13	1012.01
	1047.70	1068.37	1054.82	1050.01	1070.89	1056.61
	1221.68	1225.65	1210.74	1225.36	1229.23	1214.42
	1307.80	1315.39	1300.37	1310.01	1317.43	1302.52
	1312.64	1325.16	1304.69	1315.09	1327.59	1307.27
	1407.35	1415.43	1396.66	1410.75	1418.80	1400.01
	1466.13	1474.63	1452.23	1470.23	1478.65	1456.39
	1638.42	1651.12	1619.89	1641.49	1654.21	1622.41
	1693.28	1698.52	1671.50	1692.22	1697.36	1669.54
	3120.74	3111.39	2993.70	3135.56	3124.69	3008.09
	3127.69	3155.94	3012.91	3142.10	3169.45	3027.79
	3131.30	3113.83	3031.95	3145.54	3127.73	3045.78
	3134.44	3183.44	2980.95	3149.11	3196.34	2997.51
	3219.69	3244.34	3077.93	3235.55	3259.99	3093.60
	3219.98	3257.18	3074.14	3235.78	3272.62	3089.67
$^1CH_2$	1386.47	1374.54	1344.63	1317.19	1290.23	1262.45
	2883.38	2831.87	2807.76	2913.44	2862.40	2838.33
	2952.23	3030.41	2810.18	3001.54	3082.47	2861.46
$^3CH_2$	1044.03	992.20	940.62	1049.16	997.94	945.87
	3105.34	3057.28	3034.06	3120.95	3072.80	3049.46
	3350.24	3431.60	3219.35	3366.32	3447.33	3235.37
HCO	1101.53	1115.02	1067.10	1095.72	1109.59	1060.71
	1942.23	1907.92	1909.24	1946.04	1911.73	1912.87
	2663.65	2414.66	2387.11	2691.95	2445.31	2418.11

Table S4. TOSH nMR (n = 0-2) vibrational frequencies in cm^{-1} for the modified F1 anharmonic test set calculated at the KS-DFT and TAO-DFT B3LYP/aug-cc-pVTZ levels of electronic structure.

	KS-DFT			TAO-DFT		
Molecule	0MR	1MR	2MR	0MR	1MR	2MR
$NClF_2$	358.17	358.78	354.24	358.51	359.10	354.31
	365.80	365.30	362.39	365.55	364.88	361.61
	561.81	560.68	554.14	562.59	561.38	554.66
	687.79	686.43	680.87	686.39	684.38	678.14
	839.66	850.43	821.34	843.89	854.55	824.97
	946.55	941.42	937.05	949.21	943.92	939.37
ClF_3	406.30	407.75	404.64	404.81	406.22	397.23
	519.58	517.95	515.45	521.49	519.87	516.22
	687.61	692.17	679.06	688.93	693.23	677.96
	745.43	737.58	734.86	732.80	724.12	718.96
HOCl	740.20	732.61	729.81	735.71	727.25	724.17
	1259.56	1329.85	1228.18	1258.39	1329.33	1226.27
	3762.81	3578.91	3540.65	3774.69	3590.62	3552.13
ClNO	336.04	333.50	332.92	317.06	312.78	311.54
	610.65	613.57	603.34	587.61	590.32	577.29
	1912.14	1878.12	1872.84	1914.61	1880.39	1875.50
$ClNO_2$	371.42	369.28	367.66	364.17	360.81	358.31
	408.50	409.91	404.63	406.72	408.04	401.97
	670.88	674.65	662.58	661.91	665.32	651.53
	806.22	806.62	799.15	803.05	803.08	794.76
	1338.74	1330.77	1322.83	1339.42	1331.18	1322.91
	1755.23	1775.49	1706.45	1757.80	1778.00	1708.24
ClSN	405.00	404.18	402.56	389.98	388.32	386.50
	1367.53	1350.03	1349.37	1359.27	1341.07	1340.37
NCl_2F	337.77	338.17	333.89	336.24	336.66	331.97
	424.60	423.80	421.70	421.99	420.99	418.49
	611.87	609.95	610.47	608.45	606.28	606.05
	664.55	667.04	656.89	659.40	661.85	650.66
	842.48	826.98	819.92	845.14	828.67	821.67
Cl_2O	657.03	654.59	656.70	628.34	630.98	614.73
	664.08	665.93	652.98	651.19	648.46	650.29
$SOCl_2$	327.24	327.01	325.65	325.08	324.64	323.06
	433.90	435.86	430.36	428.82	430.70	424.29
	475.84	475.26	473.67	472.09	471.16	469.12
	1246.24	1229.35	1227.17	1247.47	1230.31	1228.03
SCl_2	492.04	495.03	488.17	468.29	472.60	464.10
	506.82	505.20	505.57	499.95	498.10	498.32
HOF	985.62	972.24	966.84	984.92	970.75	965.10
	1406.75	1445.27	1366.48	1408.99	1447.70	1368.14
	3718.29	3536.52	3498.62	3732.61	3551.17	3513.14
ONF	520.49	516.26	516.72	520.81	516.05	516.33
	778.45	774.20	769.91	777.80	773.00	768.24
	1945.11	1912.36	1907.98	1945.02	1912.04	1907.76
NSF	363.90	363.98	359.95	364.27	364.27	360.20
	623.89	618.05	616.35	626.86	620.59	618.89
	1401.20	1383.56	1382.94	1396.77	1378.43	1377.80
F_2NH	501.12	499.95	492.88	502.59	501.38	494.24
	879.54	889.39	858.44	883.15	893.09	861.69

	993.59	989.89	984.55	997.21	993.49	988.03
	1329.84	1351.07	1295.22	1332.53	1353.88	1297.65
	1463.52	1481.86	1417.44	1467.14	1485.71	1420.95
	3358.54	3183.84	3117.66	3373.13	3198.92	3132.76
N_2F_2	368.00	368.27	361.70	367.97	368.18	361.43
	421.35	421.07	414.88	422.24	421.96	415.71
	610.24	608.13	601.20	612.00	609.77	602.52
	999.29	1009.10	975.69	1004.05	1013.84	980.16
	1039.32	1031.93	1021.23	1042.93	1035.40	1024.47
	1614.00	1587.52	1581.21	1615.81	1588.94	1582.41
F_2O	481.57	480.79	475.79	480.49	479.50	474.25
	894.08	902.42	878.86	886.92	896.04	870.45
	1018.58	1013.25	1010.87	1018.96	1013.42	1010.63
F_2SO	347.18	345.09	341.46	348.56	346.45	342.81
	373.10	375.17	370.03	373.96	376.03	370.84
	500.19	499.58	495.54	501.97	501.34	497.25
	704.64	710.35	694.04	708.10	713.82	697.39
	770.75	767.87	764.47	774.49	771.56	768.12
	1316.59	1300.20	1297.37	1320.75	1304.31	1301.38
S_2F_2	598.03	590.63	592.30	601.09	593.62	595.00
	658.34	664.03	649.74	657.11	662.98	648.23
	693.64	689.00	689.05	694.69	689.93	689.88
HNO_3	478.81	1280.11	452.49	480.67	1280.81	452.45
	584.80	588.78	582.07	586.23	590.31	583.35
	646.95	646.88	635.13	650.37	650.39	638.55
	782.52	783.26	771.44	781.89	782.25	769.93
	896.05	897.21	881.15	899.70	900.69	884.20
	1315.06	1332.06	1291.26	1317.96	1334.45	1293.63
	1341.32	1342.99	1328.76	1343.29	1345.18	1330.05
	1744.01	1758.41	1709.94	1747.75	1762.22	1713.03
	3710.66	3531.85	3429.44	3722.47	3543.94	3441.19
HN_3	547.61	561.47	546.58	546.11	559.66	544.46
	615.88	623.62	604.67	616.37	623.88	604.71
	1184.10	1212.38	1160.67	1186.98	1215.62	1163.44
	1300.65	1298.34	1284.61	1303.31	1300.93	1287.14
	2268.79	2265.18	2215.40	2274.00	2270.52	2220.02
	3487.95	3322.84	3277.23	3500.14	3334.44	3288.66
H_2O	1627.30	1609.63	1562.72	1631.86	1614.26	1567.20
	3796.11	3711.03	3679.55	3810.93	3725.92	3694.41
	3898.76	3997.10	3696.17	3913.40	4011.65	3710.97
H_2O_2	370.21	1040.67	270.69	368.94	1051.16	268.12
	949.77	938.16	927.60	952.27	940.18	929.11
	1322.14	1351.21	1278.79	1324.75	1354.04	1280.96
	1434.43	1447.14	1402.93	1437.84	1450.59	1406.26
	3754.85	3854.92	3495.65	3768.89	3868.84	3509.26
	3756.01	3666.14	3570.82	3770.02	3680.18	3584.37
H_2S	1208.91	1203.33	1173.51	1212.79	1207.15	1177.11
	2682.26	2635.53	2616.86	2695.14	2648.05	2628.83
	2695.74	2747.84	2585.69	2707.36	2759.54	2596.09
H_2S_2	424.45	545.32	396.18	417.34	543.86	386.47
	497.35	493.41	489.77	494.32	489.76	485.47
	885.91	907.78	885.04	884.29	906.61	883.02
	885.97	905.41	886.03	884.84	904.61	884.63
	2629.03	2576.11	2528.99	2642.63	2588.88	2540.09

	2631.14	2682.77	2489.79	2644.72	2695.66	2500.85
NO_2	766.06	765.38	757.17	758.78	757.19	748.55
	1385.55	1377.58	1372.07	1383.73	1375.30	1369.60
	1687.93	1706.98	1633.26	1689.61	1708.62	1633.72
N_2O	616.79	624.31	609.02	617.50	624.85	609.42
	616.79	624.31	609.02	617.50	624.85	609.42
	1323.76	1313.06	1302.15	1327.68	1317.02	1306.07
	2340.07	2343.45	2285.44	2347.22	2350.80	2292.41
SO_2	513.71	513.83	509.86	512.89	512.88	508.80
	1157.38	1151.58	1148.49	1155.53	1149.35	1146.04
	1339.02	1347.54	1320.26	1339.49	1348.01	1320.03
O_3	746.09	744.09	735.86	729.68	727.11	718.22
	1189.56	1211.57	1163.74	1174.43	1196.70	1145.57
	1249.03	1242.63	1240.78	1225.76	1218.39	1215.96
COClF	405.35	405.98	402.29	406.46	407.09	403.36
	489.72	489.66	485.80	492.11	492.06	488.09
	671.35	675.61	663.56	671.50	675.67	663.45
	754.64	754.42	749.29	757.60	757.30	752.10
	1076.08	1070.04	1051.45	1080.81	1074.88	1056.17
	1908.43	1883.38	1876.68	1912.00	1886.86	1880.00
ClCN	403.98	432.37	396.78	404.42	432.62	397.09
	403.98	432.37	396.78	404.43	432.62	397.09
	741.99	736.69	732.99	745.26	739.86	736.10
	2307.61	2289.99	2268.95	2313.53	2295.88	2274.64
$COCl_2$	435.05	436.05	432.03	436.66	437.64	433.44
	554.70	552.13	552.06	557.76	555.00	554.61
	586.17	593.09	579.59	584.71	591.52	577.66
	810.14	816.15	799.01	815.22	820.99	803.32
	1866.63	1836.99	1833.79	1867.68	1837.79	1834.43
FCN	484.28	500.64	478.39	485.15	501.39	479.21
	484.28	500.64	478.39	485.15	501.39	479.21
	1093.17	1085.38	1078.20	1096.47	1088.66	1081.44
	2408.04	2403.69	2364.38	2415.35	2411.06	2371.56
COF_2	576.32	576.24	570.72	577.54	577.47	571.94
	616.03	617.34	611.18	617.37	618.68	612.50
	775.76	778.36	767.40	776.96	779.50	768.46
	963.40	959.28	955.61	966.83	962.72	959.04
	1218.13	1229.35	1190.98	1223.44	1234.65	1196.31
	1958.51	1939.18	1927.73	1964.04	1944.75	1933.12
CSF_2	418.67	419.12	415.43	418.27	418.69	414.89
	525.30	525.59	520.65	525.39	525.67	520.62
	628.65	629.99	621.88	621.88	622.80	614.15
	795.56	793.40	789.93	795.56	793.36	789.75
	1183.89	1196.20	1151.86	1188.95	1201.22	1157.05
	1352.48	1358.87	1337.37	1354.72	1361.17	1339.03
COS	527.02	539.06	521.86	526.68	538.44	521.05
	527.03	539.06	521.86	526.68	538.44	521.05
	873.91	868.83	864.33	877.13	872.03	867.37
	2108.17	2099.74	2070.31	2113.70	2105.42	2075.64
CO_2	673.82	682.54	668.73	675.01	683.74	669.83
	673.82	682.54	668.73	675.01	683.74	669.83
	1369.14	1363.45	1357.10	1372.65	1366.96	1360.59
	2400.35	2421.47	2351.67	2408.11	2429.19	2359.45
CS_2	403.13	408.09	400.93	399.57	404.32	396.62

	403.13	408.09	400.93	399.57	404.32	396.63
	673.71	671.87	669.35	674.21	672.33	669.64
	1550.76	1562.83	1526.80	1551.52	1563.64	1526.84
HCN	759.19	908.94	732.22	761.93	912.61	733.66
	759.19	908.94	732.22	761.93	912.61	733.66
	2199.78	2180.91	2169.94	2206.01	2187.10	2176.06
	3444.27	3353.70	3253.50	3458.69	3368.02	3267.75
HNCO	575.91	654.89	598.74	576.37	656.27	599.42
	637.05	644.96	627.76	638.38	646.23	628.96
	799.03	826.71	797.66	799.20	827.38	798.04
	1335.27	1329.72	1322.88	1338.76	1333.21	1326.32
	2323.78	2341.72	2277.07	2331.54	2349.46	2284.75
	3675.41	3525.28	3463.18	3688.39	3538.03	3475.77
H_2CO	1198.03	1215.97	1167.23	1197.90	1215.69	1166.09
	1262.92	1274.08	1241.39	1265.33	1276.59	1243.65
	1530.20	1531.59	1500.24	1533.70	1535.16	1503.81
	1813.17	1794.82	1787.53	1815.01	1796.48	1788.84
	2884.64	2820.13	2769.79	2901.88	2837.67	2787.34
	2939.32	3018.35	2745.91	2958.25	3037.05	2765.24
HCOOH	532.87	1055.69	432.35	534.00	1058.93	432.60
	659.70	664.86	660.10	661.00	666.22	661.44
	1032.57	1067.09	1009.19	1035.02	1069.88	1011.31
	1103.73	1097.19	1081.81	1107.86	1101.63	1086.25
	1268.77	1305.40	1229.92	1271.57	1308.05	1232.85
	1413.51	1425.71	1383.85	1418.00	1430.48	1388.40
	1855.69	1841.01	1823.87	1859.69	1844.92	1827.51
	2962.61	2822.74	2774.53	2979.50	2840.27	2791.89
	3780.88	3595.74	3498.23	3793.74	3608.20	3510.43
C_2Cl_2	371.29	395.63	362.52	371.22	395.06	361.61
	371.29	395.63	362.52	371.22	395.06	361.61
	475.76	474.90	472.89	477.22	476.30	474.20
	990.34	996.38	977.87	993.66	999.61	980.62
	2317.34	2308.50	2276.75	2321.95	2312.90	2280.85
C_2N_2	552.18	559.84	545.85	553.16	560.81	546.74
	552.18	559.84	545.85	553.16	560.81	546.74
	883.49	879.93	874.97	886.82	883.28	878.34
	2266.52	2277.94	2236.18	2270.95	2282.36	2240.33
	2429.25	2430.98	2401.98	2432.20	2433.52	2404.28
HCCCl	350.91	374.94	355.65	350.84	374.99	355.48
	350.91	374.94	355.65	350.84	374.99	355.48
	649.37	900.89	624.10	651.60	903.70	625.93
	649.37	900.89	624.10	651.60	903.71	625.94
	751.18	746.55	742.35	754.15	749.33	745.06
	2196.82	2187.78	2155.09	2202.66	2193.52	2160.44
	3477.48	3378.35	3265.99	3491.27	3392.11	3279.46
HCCF	408.47	424.14	401.65	409.44	424.71	402.48
	408.47	424.14	401.65	409.44	424.71	402.48
	617.69	918.41	580.54	620.45	921.40	583.53
	617.69	918.41	580.54	620.45	921.40	583.53
	1082.37	1075.76	1067.60	1085.39	1078.74	1070.51
	2318.78	2319.71	2271.37	2326.22	2327.14	2278.53
	3487.59	3390.20	3274.51	3501.46	3404.05	3288.19
HCCH	666.02	763.55	694.41	668.93	766.45	696.53
	666.02	763.55	694.41	668.93	766.45	696.53

	769.47	861.20	794.79	771.96	864.01	797.01
	769.47	861.20	794.79	771.96	864.01	797.01
	2068.40	2055.04	2034.33	2074.67	2061.23	2040.34
	3412.19	3481.40	3218.20	3426.05	3495.14	3231.91
	3516.92	3479.09	3368.55	3531.06	3493.26	3382.49
trans-CHClCHCl	348.94	348.77	346.87	349.98	349.79	347.84
	801.19	846.82	806.65	801.24	847.18	806.39
	805.62	810.27	789.91	811.03	815.61	795.11
	841.61	841.31	831.91	845.29	844.92	835.46
	929.90	976.86	927.35	930.96	978.06	927.93
	1219.19	1242.02	1206.10	1221.81	1244.83	1208.73
	1300.98	1309.89	1285.57	1303.40	1312.32	1287.98
	1642.86	1632.96	1613.17	1643.67	1633.55	1613.22
	3220.35	3289.58	3036.15	3233.41	3302.60	3049.36
	3223.22	3163.25	3090.19	3236.27	3176.45	3103.26
cis-CHClCHCl	421.32	428.08	420.61	419.70	426.38	418.73
	570.69	570.44	564.26	572.35	572.13	565.94
	704.33	701.27	696.28	708.90	705.77	700.74
	711.42	804.68	735.27	712.04	805.99	735.59
	844.89	848.32	830.25	848.81	852.23	834.11
	909.56	957.08	924.44	910.83	958.28	925.16
	1212.48	1232.45	1202.40	1213.81	1233.86	1203.73
	1317.76	1331.54	1298.09	1320.95	1334.84	1301.30
	1645.79	1635.48	1612.87	1647.26	1636.70	1613.59
	3199.72	3269.96	3009.60	3213.85	3284.03	3023.82
	3220.60	3161.74	3083.57	3234.35	3175.62	3097.28
CH_2CCl_2	295.68	295.68	295.68	296.15	296.15	296.15
	376.95	386.28	386.58	376.97	386.49	386.71
	473.54	477.27	474.39	472.47	476.13	473.06
	594.30	592.32	589.59	597.16	595.15	592.33
	694.95	788.11	718.02	695.08	789.07	717.93
	763.43	771.49	756.90	767.72	775.71	761.01
	903.66	962.72	926.71	904.85	964.44	928.02
	1095.67	1104.01	1089.22	1098.98	1107.22	1092.52
	1406.58	1409.21	1386.93	1409.69	1412.37	1390.05
	1661.26	1652.39	1630.47	1663.75	1654.68	1632.34
	3168.60	3114.99	3035.75	3181.86	3128.40	3048.93
	3260.95	3333.92	3060.47	3275.41	3348.31	3075.00
cis-CHFCHF	513.72	516.77	510.34	513.35	516.46	509.97
	774.59	774.65	767.03	776.15	776.22	768.57
	775.45	846.99	792.29	777.53	849.37	794.12
	876.87	926.35	884.78	879.67	929.17	887.27
	1017.58	1016.36	1010.86	1020.97	1019.78	1014.21
	1132.61	1139.60	1112.58	1136.60	1143.57	1116.60
	1283.15	1291.23	1267.70	1286.24	1294.33	1270.86
	1391.54	1401.82	1368.40	1395.22	1405.61	1372.20
	1756.76	1754.48	1721.22	1762.25	1759.92	1726.23
	3214.05	3283.64	3028.83	3228.51	3298.04	3043.50
	3236.70	3180.39	3103.75	3250.90	3194.74	3118.02
trans-CHFCHF	319.08	325.54	322.68	318.84	325.40	322.54
	336.48	339.35	331.83	336.66	339.54	331.90
	555.27	555.18	551.14	556.45	556.36	552.29
	829.40	868.11	831.20	831.27	870.24	832.96
	908.37	959.23	905.75	911.17	962.14	908.18

	1143.50	1140.66	1127.78	1147.28	1144.45	1131.60
	1160.39	1169.59	1137.72	1165.06	1174.20	1142.39
	1294.40	1309.78	1277.38	1297.52	1313.09	1280.69
	1310.89	1316.54	1294.59	1313.84	1319.60	1297.61
	1743.93	1741.75	1710.89	1748.90	1746.67	1715.36
	3216.48	3285.31	3034.48	3231.02	3299.80	3049.26
	3223.67	3165.41	3091.97	3238.30	3180.22	3106.68
OCHCHO	339.79	358.09	344.22	340.94	359.05	345.48
	551.18	549.08	542.87	552.91	550.91	544.75
	812.28	862.66	800.33	812.75	863.92	800.85
	1062.41	1056.80	1033.79	1062.94	1057.16	1033.60
	1080.68	1093.17	1070.20	1074.53	1087.08	1063.26
	1340.06	1346.76	1316.29	1339.39	1346.24	1315.47
	1380.18	1384.10	1356.40	1379.56	1383.65	1355.60
	1796.04	1807.76	1770.74	1792.68	1782.80	1771.49
	1796.98	1787.66	1776.60	1793.51	1805.25	1767.53
	2937.70	3009.98	2744.68	2954.72	3026.89	2761.32
	2941.56	2875.87	2804.32	2958.41	2892.65	2820.88
CH_2CH_2	834.72	876.67	848.07	835.77	878.11	849.30
	979.00	1006.28	974.35	981.54	1008.95	976.49
	985.00	1007.26	980.32	987.69	1010.04	982.29
	1060.40	1079.55	1056.79	1064.71	1081.52	1057.20
	1245.60	1251.75	1236.21	1248.91	1255.13	1239.50
	1380.60	1383.09	1364.88	1384.65	1387.15	1368.84
	1479.47	1482.93	1455.48	1483.91	1487.43	1459.98
	1689.03	1685.13	1659.80	1694.86	1690.90	1665.33
	3125.91	3158.28	3007.14	3140.50	3172.85	3021.90
	3139.61	3113.14	3045.09	3154.08	3127.73	3059.72
	3194.40	3231.82	3060.18	3210.29	3247.66	3076.27
	3222.66	3258.97	3090.24	3237.99	3274.28	3105.80
cyclo-C_2H_4O	819.67	851.81	827.00	822.08	854.37	829.50
	843.02	848.94	819.44	847.61	853.49	823.96
	887.61	888.38	871.86	892.26	893.01	876.51
	1048.13	1062.87	1040.23	1051.54	1066.41	1043.90
	1149.07	1159.79	1145.50	1152.59	1163.37	1149.07
	1167.60	1180.09	1161.80	1170.74	1183.42	1165.32
	1169.22	1178.28	1159.33	1172.72	1181.84	1162.94
	1176.85	1184.97	1168.78	1180.01	1188.19	1172.10
	1298.97	1296.39	1281.96	1303.89	1301.34	1286.89
	1505.69	1508.88	1481.54	1510.22	1513.47	1486.22
	1538.24	1540.46	1513.55	1543.41	1545.64	1518.76
	3086.01	3118.86	2961.18	3101.05	3133.88	2976.46
	3092.18	3063.34	2991.31	3107.13	3078.41	3006.43
	3165.00	3202.50	3023.39	3181.33	3218.78	3039.99
	3179.84	3216.77	3040.99	3195.94	3232.83	3057.31
cyclo-C_2H_4NH	777.51	805.02	799.06	780.37	808.46	802.28
	852.96	858.90	829.04	858.31	864.19	834.47
	868.01	871.13	861.23	872.51	875.57	865.57
	916.19	948.87	934.28	918.52	951.35	936.79
	1004.08	1031.89	1019.41	1006.70	1034.56	1022.07
	1114.82	1130.40	1111.06	1118.33	1134.06	1114.69
	1123.38	1139.69	1121.76	1126.62	1143.06	1125.28
	1154.26	1164.59	1145.22	1157.86	1168.21	1149.08
	1236.84	1237.40	1224.73	1241.67	1242.47	1229.64

	1264.90	1280.86	1255.13	1267.96	1284.02	1258.28
	1293.70	1295.20	1281.86	1297.42	1298.76	1285.61
	1498.15	1501.19	1475.17	1502.70	1505.78	1479.80
	1524.90	1527.34	1501.22	1529.92	1532.38	1506.30
	3104.54	3138.32	2975.54	3119.17	3152.90	2990.39
	3109.38	3080.26	3005.25	3123.92	3094.90	3019.88
	3180.04	3218.31	3034.37	3195.90	3234.09	3050.51
	3193.45	3228.51	3051.60	3209.07	3244.08	3067.41
	3507.17	3339.39	3269.16	3520.46	3352.89	3282.47
CH_2CCHCl	318.49	372.67	356.70	319.21	373.90	357.66
	503.28	504.77	500.93	503.81	505.24	501.09
	569.01	625.13	606.18	567.29	624.91	604.23
	753.05	753.21	739.78	755.66	755.61	741.80
	851.28	918.09	863.20	851.31	919.50	861.92
	914.00	968.52	935.58	914.54	969.62	936.49
	1021.73	1037.93	1019.97	1022.99	1039.31	1021.19
	1133.72	1135.18	1122.32	1135.86	1137.37	1124.19
	1276.52	1298.13	1256.75	1279.56	1301.22	1259.53
	1469.03	1472.80	1451.91	1472.74	1476.55	1455.59
	2047.23	2062.08	2005.93	2051.07	2065.74	2008.58
	3115.81	3059.48	2981.19	3129.94	3073.73	2994.94
	3187.51	3261.56	2980.88	3202.87	3276.82	2995.99
	3200.49	3071.37	3005.89	3213.81	3084.90	3019.21
CH_2CHCHO	322.41	344.10	344.73	322.08	344.03	344.85
	573.03	573.33	572.89	573.92	574.26	573.90
	614.29	667.52	632.11	615.03	668.97	633.03
	923.87	940.93	927.67	926.81	944.12	930.93
	1003.56	1041.03	1008.69	1003.14	1040.92	1008.18
	1031.27	1047.67	1027.63	1031.39	1046.49	1026.94
	1037.03	1050.87	1034.71	1037.26	1052.30	1035.25
	1173.55	1173.55	1157.06	1176.28	1176.30	1159.68
	1302.77	1307.50	1288.70	1305.49	1310.14	1291.56
	1390.46	1397.04	1369.61	1392.16	1399.03	1371.09
	1458.19	1460.33	1441.37	1460.96	1463.12	1444.18
	1677.66	1673.91	1648.88	1679.20	1675.29	1649.75
	1767.96	1751.66	1739.08	1765.87	1748.78	1736.01
	2879.30	2735.54	2683.49	2895.06	2751.61	2699.23
	3133.14	3076.64	2995.96	3147.13	3090.46	3009.80
	3170.72	3064.46	2986.46	3185.65	3078.85	3001.23
	3222.48	3260.78	3033.96	3237.89	3275.92	3049.24
cyclo-C_3H_6	742.32	777.14	775.08	744.35	779.31	777.30
	743.52	776.36	769.72	745.51	778.55	772.08
	861.17	885.99	882.74	862.62	887.63	884.49
	878.14	881.91	856.95	883.38	887.15	862.37
	878.87	877.25	858.97	884.12	882.48	864.38
	1051.72	1073.75	1061.33	1055.14	1077.16	1064.60
	1053.30	1073.36	1056.22	1056.67	1076.81	1059.65
	1094.87	1105.84	1098.66	1098.00	1109.10	1102.10
	1153.29	1159.52	1144.93	1157.27	1163.55	1149.07
	1210.79	1207.18	1196.24	1216.10	1212.54	1201.63
	1213.17	1217.05	1204.77	1216.64	1220.54	1208.37
	1213.51	1217.24	1204.07	1216.99	1220.73	1207.66
	1475.76	1479.60	1458.29	1480.36	1484.24	1463.00
	1477.07	1480.12	1455.52	1481.65	1484.76	1460.25

	1519.46	1521.25	1499.61	1524.65	1526.45	1504.89
	3126.12	3152.25	3000.83	3140.85	3166.77	3015.76
	3126.46	3146.51	3004.24	3141.19	3161.44	3019.03
	3133.66	3114.74	3037.82	3148.12	3129.28	3052.35
	3198.18	3235.68	3054.56	3214.06	3251.52	3070.61
	3198.47	3235.39	3054.61	3214.35	3251.22	3070.65
	3219.69	3243.98	3097.18	3235.02	3259.30	3112.62
$CH_2CHCHCH_2$	519.36	522.32	530.58	519.71	522.78	526.95
	540.43	598.11	593.38	541.04	599.33	593.67
	781.85	797.42	806.87	781.68	797.27	806.12
	901.31	908.67	908.86	904.74	912.21	906.71
	943.29	970.93	967.89	944.07	971.94	968.07
	945.11	970.29	968.62	945.65	971.18	968.92
	1002.48	1025.32	1013.09	1004.38	1026.55	1012.85
	1007.31	1019.16	1021.44	1008.92	1020.87	1014.80
	1057.11	1073.18	1058.71	1059.45	1075.49	1059.78
	1229.12	1228.64	1215.77	1232.65	1232.17	1217.29
	1317.76	1319.58	1306.84	1320.07	1321.76	1306.78
	1324.03	1329.11	1311.64	1326.47	1331.64	1311.59
	1419.50	1420.99	1404.74	1422.85	1424.37	1403.71
	1479.18	1480.79	1461.44	1483.17	1484.81	1460.53
	1650.81	1656.30	1625.06	1653.94	1659.40	1626.85
	1701.90	1701.04	1673.40	1701.18	1700.05	1671.81
	3124.07	3099.29	2993.03	3138.47	3111.54	3008.65
	3134.20	3156.49	3018.44	3148.24	3170.72	3034.90
	3136.88	3170.95	3003.04	3151.03	3128.96	3047.49
	3137.09	3115.16	3030.47	3151.23	3184.00	3021.44
	3220.60	3245.36	3077.41	3236.09	3261.01	3096.42
	3220.89	3259.44	3073.02	3236.30	3274.93	3091.80
$^1CH_2$	1400.28	1386.27	1356.28	1329.02	1308.32	1280.17
	2909.08	2846.23	2822.46	2939.07	2875.39	2851.71
	2971.58	3043.56	2822.17	3019.84	3091.54	2869.74
$^3CH_2$	1051.02	1002.89	950.81	1056.10	1308.32	955.74
	3123.23	3062.23	3039.20	3138.63	2875.39	3054.62
	3359.75	3431.26	3217.11	3375.53	3091.54	3233.11
HCO	1101.93	1114.22	1065.72	1095.80	1108.56	1059.11
	1929.75	1903.62	1904.71	1934.01	1907.91	1908.84
	2654.07	2417.02	2389.13	2681.77	2447.26	2419.80

Table S5. TOSH nMR (n = 0-2) vibrational frequencies in cm^{-1} for the modified F1 anharmonic test set calculated at the KS-DFT and TAO-DFT B97-1/aug-cc-pVTZ levels of electronic structure.

	KS-DFT			TAO-DFT		
Molecule	0MR	1MR	2MR	0MR	1MR	2MR
$NClF_2$	360.53	361.15	356.84	360.84	361.44	356.92
	372.96	372.52	369.52	372.81	372.23	368.96
	569.60	568.58	562.22	570.38	569.29	562.79
	703.35	701.62	696.12	702.03	699.67	693.61
	865.81	875.60	847.36	870.07	879.77	851.14
	967.20	962.03	957.71	969.91	964.59	960.14
ClF_3	415.76	417.17	409.04	414.43	415.81	407.09
	532.56	530.66	526.89	534.57	532.67	528.51
	704.12	708.10	693.39	705.87	709.60	694.09
	755.53	748.57	744.75	744.15	736.50	731.53
HOCl	756.23	748.90	746.41	752.28	744.21	741.48
	1264.14	1335.35	1233.33	1263.06	1335.09	1231.74
	3795.83	3612.69	3574.45	3808.10	3624.86	3586.41
ClNO	345.13	343.07	343.03	329.13	325.21	324.30
	622.17	624.63	615.34	602.17	604.10	592.23
	1900.80	1867.76	1862.64	1902.17	1868.87	1864.01
$ClNO_2$	388.31	385.66	383.86	382.88	379.12	376.47
	413.58	414.88	409.73	412.14	413.34	407.53
	674.15	677.76	666.06	665.74	669.00	655.76
	808.46	808.84	801.47	805.66	805.72	797.51
	1340.41	1333.07	1325.80	1340.93	1333.32	1325.70
	1754.35	1774.47	1709.74	1756.65	1776.72	1711.22
ClSN	419.68	418.61	417.39	406.67	404.71	403.42
	1366.20	1348.12	1347.68	1357.42	1338.61	1338.17
NCl_2F	343.67	344.04	340.10	342.41	342.79	338.48
	431.15	430.62	428.90	428.70	427.99	425.90
	628.16	626.37	626.68	625.03	622.97	622.51
	687.18	689.12	678.89	682.49	684.34	673.05
	868.98	853.22	847.10	871.77	855.18	849.13
Cl_2O	671.70	669.04	670.56	652.28	653.70	638.43
	685.55	686.36	673.96	666.50	663.57	664.83
$SOCl_2$	336.34	336.26	334.63	334.49	334.23	332.39
	451.62	453.31	447.51	447.13	448.76	442.12
	490.15	489.36	487.77	487.00	485.85	483.85
	1263.74	1246.42	1244.38	1265.28	1247.71	1245.57
SCl_2	510.74	513.59	507.18	488.63	492.66	484.88
	521.28	519.92	520.40	515.06	513.51	513.89
HOF	1007.74	993.80	988.54	1007.71	993.04	987.55
	1419.63	1460.03	1381.72	1422.07	1462.66	1383.65
	3753.47	3571.71	3534.02	3768.08	3586.66	3548.86
ONF	535.70	530.97	532.05	536.71	531.48	532.37
	794.41	789.99	786.01	794.35	789.40	785.02
	1928.48	1896.96	1893.24	1928.42	1896.65	1893.03
NSF	367.96	368.36	364.50	368.31	368.63	364.71
	632.45	627.26	625.67	635.70	630.06	628.46
	1400.75	1382.59	1382.15	1396.11	1377.24	1376.80
F_2NH	507.29	506.19	499.37	508.75	507.62	500.73
	902.12	911.22	881.29	905.77	914.95	884.64

	1010.98	1007.27	1001.95	1014.62	1010.90	1005.49
	1336.61	1357.05	1302.38	1339.35	1359.95	1304.93
	1467.49	1486.48	1423.58	1471.15	1490.38	1427.17
	3364.73	3194.08	3129.04	3379.54	3209.36	3144.34
N_2F_2	370.65	371.09	364.61	370.60	370.99	364.34
	422.97	422.87	416.84	423.82	423.72	417.63
	618.98	617.18	610.44	620.71	618.81	611.81
	1021.87	1031.26	999.13	1026.61	1035.99	1003.65
	1057.72	1050.33	1040.27	1061.35	1053.85	1043.60
	1600.16	1575.70	1568.91	1601.98	1577.08	1570.09
F_2O	491.59	490.81	485.71	490.79	489.82	484.48
	918.07	926.54	901.74	912.13	921.27	894.59
	1039.36	1033.79	1031.26	1040.17	1034.40	1031.50
F_2SO	354.61	352.61	348.92	356.00	353.98	350.27
	378.31	380.23	375.03	379.16	381.07	375.84
	508.35	507.70	503.59	510.15	509.48	505.31
	715.68	722.25	704.71	719.44	725.96	708.25
	781.24	778.41	775.17	785.22	782.31	778.97
	1336.35	1319.86	1317.15	1340.55	1324.02	1321.23
S_2F_2	593.50	587.49	589.03	596.33	590.19	591.49
	671.82	678.18	663.08	670.51	677.09	661.54
	707.64	702.96	703.15	708.63	703.86	703.97
HNO_3	488.62	1247.51	431.81	490.57	1246.47	429.42
	588.08	592.36	584.65	589.37	593.66	585.28
	660.23	660.55	649.33	663.12	663.46	652.24
	784.20	784.90	773.55	783.46	783.79	771.92
	915.00	915.34	898.32	918.97	919.09	901.76
	1321.67	1340.86	1294.61	1324.67	1343.25	1296.02
	1349.49	1349.62	1335.90	1351.59	1351.73	1336.90
	1750.88	1764.84	1718.65	1754.70	1768.73	1721.65
	3743.44	3564.74	3460.08	3755.51	3577.11	3472.02
HN_3	542.50	556.04	540.69	540.89	554.09	538.42
	611.13	618.62	599.66	611.60	618.84	599.64
	1184.66	1209.63	1162.34	1187.64	1212.97	1165.14
	1296.86	1295.33	1281.36	1299.47	1297.87	1283.87
	2254.61	2251.18	2202.49	2259.87	2256.57	2207.14
	3492.50	3331.83	3286.50	3504.90	3343.69	3298.18
H_2O	1634.46	1617.02	1569.69	1639.06	1621.67	1574.20
	3829.83	3744.72	3712.85	3844.94	3759.90	3728.02
	3932.91	4030.44	3730.35	3947.82	4045.25	3745.45
H_2O_2	381.02	1023.99	275.25	379.98	1034.66	274.32
	976.16	963.81	953.02	979.11	966.32	955.08
	1336.38	1364.63	1289.65	1339.13	1367.57	1291.93
	1443.39	1455.96	1410.82	1446.81	1459.42	1414.17
	3787.23	3886.73	3528.54	3801.56	3900.92	3542.49
	3787.96	3697.97	3603.05	3802.27	3712.33	3616.96
H_2S	1202.76	1204.48	1175.19	1206.76	1208.56	1179.06
	2671.23	2625.12	2606.21	2683.89	2638.02	2619.24
	2686.09	2736.35	2577.30	2697.45	2748.65	2588.65
H_2S_2	427.01	533.68	390.41	419.84	530.19	378.65
	510.61	507.05	504.22	507.59	503.50	500.12
	882.49	906.08	889.68	880.94	904.42	886.96
	883.15	903.98	891.11	882.11	902.68	889.09
	2616.52	2567.45	2522.55	2630.33	2580.52	2533.87

	2618.43	2671.81	2484.11	2632.23	2685.19	2495.32
NO_2	764.87	764.26	756.29	757.28	755.75	747.35
	1389.15	1381.64	1376.70	1387.11	1379.14	1374.00
	1688.78	1707.89	1638.00	1690.17	1709.24	1638.08
N_2O	612.85	620.37	605.23	613.54	620.88	605.52
	612.85	620.37	605.23	613.54	620.88	605.52
	1323.02	1313.27	1303.28	1326.91	1317.18	1307.13
	2327.46	2331.77	2274.29	2334.74	2339.22	2281.37
SO_2	517.35	517.47	513.47	516.54	516.53	512.42
	1175.51	1169.56	1166.20	1173.91	1167.58	1164.00
	1361.53	1369.84	1342.40	1362.31	1370.59	1342.48
O_3	751.32	749.48	741.52	735.50	733.10	724.55
	1235.83	1255.69	1209.60	1221.83	1241.90	1192.78
	1276.32	1269.82	1267.47	1254.32	1246.91	1244.00
COClF	404.91	405.63	402.26	405.98	406.70	403.29
	492.22	492.31	488.87	494.45	494.55	491.06
	671.47	675.92	664.11	671.52	675.89	663.89
	760.80	760.76	755.99	763.64	763.56	758.76
	1097.88	1091.23	1072.68	1102.61	1096.08	1077.49
	1919.52	1895.31	1887.83	1923.15	1898.84	1891.20
ClCN	399.28	427.65	391.66	399.60	427.79	391.83
	399.28	427.65	391.66	399.60	427.79	391.83
	740.12	735.43	731.88	743.09	738.33	734.73
	2300.97	2283.34	2262.30	2306.93	2289.26	2268.05
$COCl_2$	436.78	437.86	434.47	438.16	439.23	435.74
	563.28	561.27	561.81	566.00	563.87	564.23
	583.09	590.48	577.36	581.39	588.68	575.17
	825.67	831.63	816.32	830.29	836.13	820.41
	1880.99	1851.98	1848.47	1882.12	1852.86	1849.27
FCN	481.62	497.81	475.68	482.44	498.57	476.37
	481.62	497.81	475.68	482.44	498.57	476.37
	1101.19	1093.52	1086.27	1104.55	1096.86	1089.56
	2402.62	2399.00	2358.62	2410.15	2406.57	2366.00
COF_2	580.41	580.40	574.97	581.64	581.63	576.21
	617.96	619.29	613.14	619.31	620.65	614.49
	778.95	781.67	770.84	780.15	782.81	771.91
	974.33	970.08	966.01	977.79	973.56	969.51
	1239.98	1251.00	1211.65	1245.36	1256.38	1217.08
	1966.91	1948.33	1936.20	1972.59	1954.05	1941.75
CSF_2	418.73	419.15	415.55	418.31	418.71	415.01
	527.69	527.99	523.13	527.78	528.09	523.13
	632.65	634.14	626.03	625.83	626.93	618.29
	800.48	798.27	794.61	800.37	798.14	794.39
	1211.16	1223.11	1178.38	1216.19	1228.12	1183.58
	1363.94	1370.05	1348.67	1366.30	1372.48	1350.57
COS	525.68	538.14	520.95	525.28	537.45	520.00
	525.68	538.15	520.95	525.28	537.45	520.00
	868.89	864.17	860.10	872.35	867.56	863.25
	2118.27	2109.63	2081.31	2123.91	2115.39	2086.66
CO_2	672.86	681.67	667.71	674.05	682.85	668.83
	672.86	681.67	667.71	674.05	682.85	668.83
	1372.41	1366.75	1360.28	1375.97	1370.31	1363.82
	2410.66	2431.36	2362.37	2418.53	2439.18	2370.25
CS_2	400.52	406.50	399.82	396.88	402.49	395.43

	400.52	406.50	399.82	396.88	402.49	395.43
	669.82	668.22	666.23	670.44	668.77	666.57
	1546.13	1558.48	1522.36	1547.16	1559.52	1522.59
HCN	751.99	909.51	732.64	754.79	912.72	735.02
	751.99	909.51	732.64	754.79	912.72	735.03
	2191.25	2172.42	2161.59	2197.62	2178.74	2167.85
	3435.35	3348.16	3248.88	3450.13	3362.80	3263.48
HNCO	573.10	646.87	594.52	573.56	648.07	595.14
	634.24	642.28	624.83	635.58	643.56	626.03
	810.58	839.26	810.29	810.73	839.98	810.62
	1331.09	1325.56	1318.41	1334.67	1329.13	1321.95
	2322.42	2339.89	2275.64	2330.30	2347.76	2283.43
	3678.12	3531.15	3468.59	3691.34	3544.16	3481.44
H_2CO	1194.13	1213.09	1166.11	1193.99	1212.78	1164.90
	1259.24	1270.89	1239.89	1261.70	1273.44	1242.15
	1526.44	1528.31	1498.46	1530.06	1531.99	1502.09
	1822.46	1803.86	1797.28	1824.40	1805.61	1798.70
	2876.66	2814.64	2766.62	2894.21	2832.48	2784.43
	2933.21	3011.96	2745.75	2952.46	3031.03	2765.32
HCOOH	535.31	1060.22	433.19	536.41	1063.75	433.58
	660.29	665.61	660.99	661.58	666.96	662.32
	1031.22	1065.79	1008.58	1033.67	1068.61	1010.70
	1114.75	1108.93	1093.24	1118.87	1113.43	1097.70
	1275.13	1310.80	1237.53	1278.02	1313.47	1240.58
	1411.52	1422.12	1380.38	1416.14	1427.00	1385.02
	1865.59	1851.11	1833.69	1869.70	1855.15	1837.44
	2953.87	2819.14	2771.02	2971.14	2836.95	2788.64
	3814.55	3629.84	3532.24	3827.75	3642.71	3544.86
C_2Cl_2	353.95	380.56	345.28	353.80	379.84	344.14
	353.95	380.56	345.28	353.80	379.84	344.14
	475.65	474.81	472.64	476.82	475.96	473.77
	992.47	998.11	980.58	995.13	1000.77	982.81
	2311.88	2302.98	2270.12	2316.34	2307.24	2274.13
C_2N_2	545.48	553.18	539.16	546.43	554.17	539.90
	545.48	553.18	539.16	546.43	554.17	539.90
	878.65	875.12	870.16	882.14	878.62	873.66
	2258.58	2269.92	2228.19	2263.05	2274.37	2232.35
	2423.22	2425.03	2395.97	2426.07	2427.46	2398.13
HCCCl	343.22	368.31	347.45	343.00	368.00	347.06
	343.22	368.31	347.45	343.00	368.00	347.06
	634.12	910.28	619.84	636.29	913.04	621.63
	634.12	910.28	619.84	636.29	913.04	621.64
	751.42	747.09	742.51	754.07	749.62	745.05
	2189.40	2180.17	2146.72	2195.27	2185.93	2152.09
	3469.96	3374.07	3262.08	3484.12	3388.14	3275.81
HCCF	398.97	415.24	393.64	399.99	416.07	393.97
	398.97	415.24	393.64	399.99	416.07	393.97
	600.35	925.37	580.58	602.98	928.82	582.42
	600.35	925.37	580.58	602.99	928.82	582.42
	1091.59	1085.20	1076.71	1094.66	1088.22	1079.66
	2313.97	2315.53	2265.34	2321.61	2323.14	2272.66
	3481.19	3386.97	3270.59	3495.43	3401.12	3284.54
HCCH	649.88	756.92	692.51	652.70	759.52	694.54
	649.89	756.93	692.51	652.70	759.52	694.54

	763.22	861.23	803.54	765.67	863.97	805.58
	763.22	861.23	803.54	765.67	863.97	805.58
	2058.58	2044.88	2024.05	2065.03	2051.20	2030.12
	3404.01	3473.33	3214.44	3418.26	3487.48	3228.39
	3509.74	3473.34	3364.54	3524.30	3487.88	3378.75
trans-CHClCHCl	349.21	349.11	347.64	350.17	350.04	348.43
	789.32	838.17	801.31	789.23	838.44	800.92
	815.70	820.71	802.26	821.04	825.87	807.03
	844.65	845.06	837.37	848.26	848.55	840.67
	924.53	974.54	929.99	925.42	975.60	930.39
	1208.00	1233.06	1201.09	1210.64	1235.93	1203.84
	1292.08	1302.24	1281.52	1294.51	1304.71	1284.02
	1635.73	1626.12	1607.02	1636.43	1626.59	1606.85
	3212.13	3281.52	3033.60	3225.58	3294.94	3047.15
	3215.62	3157.81	3087.44	3229.06	3171.38	3100.87
cis-CHClCHCl	417.55	424.22	416.79	415.72	422.31	414.69
	569.72	569.77	564.60	571.28	571.35	566.06
	707.02	802.46	735.26	707.49	803.80	735.67
	712.09	709.77	706.34	716.71	714.19	710.47
	849.67	853.78	837.49	853.63	857.61	841.00
	898.27	947.87	916.26	899.33	949.03	917.04
	1201.37	1222.29	1194.27	1202.71	1223.69	1195.57
	1308.53	1323.32	1292.45	1311.77	1326.65	1295.63
	1639.27	1629.37	1607.37	1640.59	1630.43	1607.85
	3192.08	3262.29	3006.51	3206.63	3276.78	3021.08
	3213.51	3156.72	3080.13	3227.69	3170.99	3094.19
CH_2CCl_2	293.61	293.61	293.61	294.02	294.02	294.02
	372.44	382.43	383.88	372.38	382.57	383.90
	468.62	472.58	469.48	467.40	471.22	467.73
	599.09	597.57	595.96	601.95	600.27	598.31
	692.38	786.41	718.85	692.39	787.34	718.69
	773.24	782.86	771.72	777.28	786.76	775.19
	893.00	954.32	919.14	894.20	956.09	920.51
	1093.42	1101.52	1089.67	1096.84	1104.83	1092.93
	1398.92	1402.18	1382.05	1402.13	1405.43	1385.21
	1654.08	1645.36	1624.39	1656.54	1647.62	1626.12
	3159.32	3107.58	3029.93	3172.92	3121.31	3043.40
	3255.95	3328.82	3059.69	3270.77	3343.58	3074.52
cis-CHFCHF	515.17	517.94	511.54	514.73	517.56	511.11
	773.22	847.60	798.73	775.27	850.00	800.52
	777.74	777.80	769.93	779.31	779.38	771.49
	866.62	919.92	881.94	869.49	922.77	884.41
	1023.55	1022.24	1016.02	1026.98	1025.69	1019.34
	1145.58	1151.85	1124.15	1149.68	1155.89	1128.23
	1283.76	1291.37	1269.53	1287.01	1294.61	1272.75
	1392.43	1402.38	1370.70	1396.30	1406.31	1374.55
	1753.09	1751.61	1717.36	1758.70	1757.17	1722.45
	3203.83	3273.42	3023.11	3218.72	3288.24	3038.06
	3227.07	3172.64	3097.90	3241.68	3187.35	3112.45
trans-CHFCHF	318.24	324.92	321.88	317.97	324.76	321.72
	338.10	341.17	333.88	338.24	341.31	333.92
	557.37	557.29	553.28	558.56	558.48	554.43
	820.08	860.43	824.22	821.92	862.59	826.08
	906.90	959.31	907.65	909.62	962.23	910.17

	1154.50	1151.99	1139.77	1158.38	1155.86	1143.66
	1175.81	1185.10	1153.93	1180.57	1189.77	1158.68
	1293.49	1308.49	1276.23	1296.79	1311.95	1279.73
	1307.78	1313.35	1291.58	1310.83	1316.50	1294.71
	1738.79	1737.58	1705.89	1743.90	1742.63	1710.46
	3205.72	3274.56	3027.91	3220.64	3289.47	3043.08
	3213.01	3156.59	3084.91	3228.02	3171.76	3100.02
OCHCHO	334.37	352.99	337.65	335.61	354.01	338.96
	547.38	545.34	539.45	549.14	547.21	541.38
	805.87	857.54	795.15	806.42	858.95	795.83
	1057.78	1052.65	1030.65	1058.20	1052.94	1030.39
	1075.32	1088.12	1065.79	1068.96	1081.83	1058.66
	1332.68	1338.35	1306.22	1332.01	1337.84	1305.38
	1375.36	1378.32	1348.90	1374.69	1377.80	1348.02
	1805.76	1817.30	1780.66	1803.38	1814.95	1777.63
	1807.91	1798.53	1787.71	1803.80	1793.88	1782.86
	2928.71	3000.62	2740.28	2946.11	3017.95	2757.23
	2932.77	2869.34	2799.13	2950.01	2886.47	2816.04
CH_2CH_2	826.45	869.71	843.68	827.54	871.20	844.96
	974.57	1002.61	972.85	977.13	1005.30	975.10
	975.07	998.37	972.54	977.79	1001.18	974.80
	1058.67	1075.51	1053.43	1062.49	1077.96	1054.84
	1240.11	1246.30	1232.00	1243.50	1249.76	1235.43
	1375.20	1377.79	1360.55	1379.35	1381.95	1364.62
	1470.93	1474.74	1449.18	1475.50	1479.37	1453.81
	1680.23	1676.51	1652.29	1686.13	1682.38	1657.90
	3117.23	3149.92	3002.19	3132.23	3164.88	3017.20
	3131.88	3106.55	3040.82	3146.74	3121.48	3055.67
	3189.69	3227.26	3059.20	3206.00	3243.50	3075.56
	3218.09	3254.58	3089.55	3233.84	3270.29	3105.36
cyclo-C_2H_4O	813.04	846.89	828.41	815.55	849.54	830.85
	837.85	844.72	816.11	842.55	849.37	820.70
	873.17	876.04	861.80	877.97	880.79	866.55
	1045.98	1060.85	1041.02	1049.49	1064.48	1044.70
	1143.81	1155.55	1145.79	1147.44	1159.22	1149.28
	1158.86	1172.25	1158.46	1162.15	1175.70	1161.91
	1168.02	1176.80	1158.69	1171.60	1180.46	1162.46
	1169.22	1177.70	1164.38	1172.49	1181.03	1167.83
	1294.07	1291.65	1278.97	1299.12	1296.72	1283.98
	1498.99	1502.31	1475.98	1503.65	1507.04	1480.91
	1532.04	1534.35	1508.72	1537.34	1539.68	1514.17
	3076.22	3109.67	2957.96	3091.62	3125.08	2973.57
	3082.95	3055.61	2988.29	3098.26	3071.04	3003.74
	3158.71	3196.63	3023.58	3175.41	3213.30	3040.52
	3173.68	3211.03	3041.26	3190.14	3227.48	3057.91
cyclo-C_2H_4NH	768.48	794.21	791.42	771.53	797.97	794.91
	839.91	846.32	818.53	845.28	851.65	823.98
	850.60	854.65	847.85	855.08	859.00	852.01
	909.71	942.95	929.87	912.11	945.50	932.51
	999.33	1026.63	1013.44	1001.98	1029.39	1016.23
	1107.49	1122.91	1103.28	1111.04	1126.62	1106.95
	1112.04	1129.13	1112.07	1115.39	1132.59	1115.57
	1148.34	1158.25	1139.47	1152.04	1161.99	1143.46
	1228.55	1229.25	1217.70	1233.49	1234.39	1222.69

	1258.81	1276.38	1252.90	1261.94	1279.62	1256.19
	1290.63	1292.42	1279.27	1294.42	1296.06	1283.14
	1489.70	1492.81	1467.15	1494.38	1497.53	1471.92
	1516.80	1519.32	1493.90	1521.95	1524.49	1499.13
	3097.02	3131.22	2972.59	3112.00	3146.16	2987.73
	3102.32	3074.52	3002.40	3117.20	3089.49	3017.32
	3176.83	3215.34	3035.62	3193.03	3231.47	3052.04
	3190.37	3225.58	3052.92	3206.33	3241.52	3069.01
	3512.27	3349.90	3280.14	3525.77	3363.62	3293.67
CH_2CCHCl	314.52	371.30	362.99	315.24	372.55	363.90
	503.55	505.20	502.40	503.88	505.48	502.37
	562.95	621.50	609.91	561.11	621.20	607.73
	760.16	761.06	749.11	762.58	763.35	751.05
	843.49	913.09	864.54	843.29	914.38	862.77
	904.13	962.70	934.86	904.60	963.71	935.57
	1015.01	1032.00	1016.88	1016.25	1033.41	1018.17
	1127.61	1129.75	1118.47	1129.76	1131.95	1120.32
	1268.89	1291.79	1254.01	1271.92	1294.86	1256.74
	1460.58	1464.98	1446.90	1464.36	1468.82	1450.73
	2038.62	2053.58	1997.47	2042.47	2057.25	2000.06
	3109.21	3055.03	2979.00	3123.62	3069.54	2992.91
	3185.27	3259.25	2983.70	3200.92	3274.82	2998.97
	3194.67	3069.64	3004.94	3208.36	3083.48	3018.55
CH_2CHCHO	317.50	339.98	340.12	317.15	340.03	340.55
	569.31	569.59	568.66	570.23	570.56	569.72
	610.21	664.04	631.14	610.98	665.53	632.07
	916.67	933.54	919.29	919.70	936.80	922.59
	997.85	1037.84	1004.34	997.32	1037.66	1003.66
	1023.99	1041.27	1020.96	1024.01	1040.71	1020.56
	1033.99	1048.08	1033.68	1034.07	1048.89	1033.91
	1165.86	1165.63	1148.07	1168.63	1168.40	1150.68
	1295.52	1299.44	1278.54	1298.33	1302.18	1281.58
	1382.97	1390.20	1364.23	1384.73	1392.35	1365.97
	1450.75	1452.87	1433.76	1453.55	1455.73	1436.71
	1669.22	1665.17	1639.42	1670.71	1666.54	1640.25
	1778.37	1761.21	1749.74	1776.38	1758.50	1746.80
	2870.71	2732.19	2680.84	2886.77	2748.46	2696.75
	3122.91	3068.27	2988.83	3137.27	3082.46	3002.99
	3164.16	3061.45	2983.74	3179.52	3076.25	2998.86
	3216.15	3254.12	3031.97	3231.96	3269.72	3047.52
cyclo-C_3H_6	734.89	769.25	766.09	736.97	771.47	768.43
	736.02	768.22	760.93	738.04	770.48	763.51
	850.15	875.47	873.32	851.63	877.17	875.22
	853.44	854.77	835.90	858.78	860.06	841.36
	854.59	857.19	835.20	859.93	862.56	840.62
	1041.48	1064.10	1051.08	1044.91	1067.54	1054.40
	1043.18	1063.07	1044.63	1046.55	1066.56	1048.16
	1079.95	1091.03	1082.61	1083.20	1094.40	1086.20
	1147.37	1153.54	1138.06	1151.45	1157.66	1142.31
	1199.92	1196.74	1187.17	1205.27	1202.13	1192.57
	1204.85	1208.63	1195.97	1208.43	1212.23	1199.69
	1205.08	1208.81	1196.36	1208.64	1212.40	1200.11
	1465.92	1469.93	1449.45	1470.63	1474.68	1454.32
	1467.47	1470.67	1446.13	1472.15	1475.42	1451.10

	1509.51	1511.47	1491.04	1514.79	1516.77	1496.49
	3118.05	3137.59	3000.54	3133.15	3154.34	3015.21
	3118.29	3144.63	2999.19	3133.35	3158.38	3014.99
	3125.97	3108.25	3036.10	3140.78	3123.13	3051.01
	3194.58	3232.30	3055.88	3210.82	3248.41	3072.32
	3194.78	3232.39	3056.62	3210.98	3248.59	3073.06
	3216.05	3240.80	3099.35	3231.72	3256.47	3115.13
$CH_2CHCHCH_2$	514.05	517.36	523.01	514.38	517.80	523.63
	535.84	595.94	600.04	536.40	597.25	601.23
	778.43	793.22	806.82	778.01	792.99	806.02
	895.73	903.11	899.67	899.26	906.77	903.54
	936.90	965.49	967.89	937.53	966.44	968.62
	937.34	963.85	968.12	937.82	964.70	968.83
	996.13	1018.76	1008.98	997.70	1020.09	1009.21
	998.80	1010.80	1006.86	1000.46	1012.57	1008.73
	1052.74	1069.40	1058.45	1054.92	1071.68	1059.88
	1222.54	1222.02	1207.51	1226.16	1225.66	1211.25
	1310.92	1312.66	1298.19	1313.29	1314.92	1300.63
	1315.58	1320.58	1300.47	1318.11	1323.21	1303.34
	1411.62	1413.13	1394.09	1415.04	1416.57	1397.41
	1470.75	1472.36	1449.91	1474.86	1476.49	1454.06
	1641.98	1647.32	1615.87	1645.09	1650.41	1618.37
	1693.71	1692.67	1665.11	1692.84	1691.53	1663.21
	3117.10	3105.68	2994.41	3131.91	3118.21	3009.01
	3125.07	3150.53	3014.42	3139.54	3164.44	3029.61
	3128.35	3108.72	3032.55	3142.64	3122.91	3046.46
	3130.24	3178.32	2983.81	3144.90	3191.40	3001.27
	3215.15	3240.60	3080.34	3231.09	3256.61	3096.04
	3215.37	3254.09	3075.88	3231.24	3270.01	3091.40
$^1CH_2$	1399.23	1386.23	1356.47	1334.18	1314.85	1286.92
	2891.04	2830.59	2808.25	2919.80	2858.54	2836.42
	2955.54	3027.36	2811.12	3001.27	3072.97	2856.27
$^3CH_2$	1054.69	1006.52	956.80	1059.58	1011.66	961.39
	3107.76	3048.91	3027.55	3123.40	3064.64	3043.16
	3343.87	3415.38	3205.66	3359.94	3431.43	3221.85
HCO	1101.69	1112.95	1064.78	1095.77	1107.33	1058.11
	1936.15	1910.47	1911.15	1940.43	1914.77	1915.28
	2658.46	2435.30	2408.01	2686.32	2465.61	2438.65

Table S6. TOSH 2MR vibrational frequencies in cm^{-1} for the modified F1 anharmonic test set calculated using the 6-311++G(d,p) basis set and modified KS-B3LYP functional with different amounts of exact Hartree-Fock exchange parameterized between 10-20%.

Molecule	10% HF	12% HF	14% HF	16% HF	18% HF	20% HF
$NClF_2$	337.23	339.87	342.66	345.37	348.02	350.61
	337.03	341.18	345.07	348.91	352.68	356.39
	532.55	536.88	541.07	545.12	549.04	552.84
	646.21	651.66	657.01	662.28	667.46	672.57
	759.07	769.46	779.78	790.02	800.16	810.22
	896.74	903.21	909.66	916.09	922.51	928.91
ClF_3	348.05	353.32	358.34	363.14	367.74	372.15
	461.91	464.83	467.76	470.71	473.68	476.67
	635.89	643.16	650.22	656.90	658.48	660.01
	651.75	653.55	655.26	657.08	663.75	670.25
HOCl	675.43	679.09	682.73	686.35	689.94	693.51
	1175.30	1180.42	1185.48	1190.48	1195.42	1200.31
	3479.90	3494.10	3508.22	3522.23	3536.15	3549.97
ClNO	317.82	318.77	319.63	320.41	321.12	321.74
	584.93	587.04	589.09	591.07	593.01	594.90
	1855.78	1864.38	1872.79	1881.02	1889.05	1896.89
$ClNO_2$	347.37	349.70	352.09	354.56	357.10	359.72
	393.50	396.09	398.67	401.25	403.82	406.39
	641.85	645.40	648.92	652.42	655.90	659.35
	785.14	788.46	791.76	795.04	798.32	801.58
	1310.72	1315.40	1319.98	1324.47	1328.89	1333.23
	1706.11	1710.00	1713.70	1717.22	1720.56	1723.74
ClSN	375.42	377.08	378.70	380.34	381.92	383.51
	1317.64	1323.65	1329.44	1335.02	1340.38	1345.52
NCl_2F	312.52	316.22	319.81	323.32	326.73	330.06
	399.03	402.68	406.24	409.72	413.11	416.40
	587.49	591.12	594.59	597.93	601.13	604.24
	599.97	607.76	615.56	623.34	631.11	638.84
	770.55	778.54	786.58	794.67	802.79	810.93
Cl_2O	560.60	570.42	580.10	589.63	598.99	608.19
	613.12	616.06	618.97	621.85	624.71	627.56
$SOCl_2$	302.38	304.74	307.07	309.38	311.66	313.93
	396.92	399.37	401.85	404.35	406.87	409.41
	440.17	442.82	445.43	448.03	450.61	453.17
	1182.37	1188.09	1193.65	1199.03	1204.25	1209.31
SCl_2	450.12	452.80	455.49	458.18	460.86	463.55
	472.68	474.81	476.92	478.99	481.05	483.08
HOF	896.32	902.29	908.22	914.10	919.94	925.74
	1321.93	1329.15	1336.28	1343.34	1350.32	1357.23
	3434.84	3449.44	3463.94	3478.34	3492.65	3506.86
ONF	481.35	484.00	486.60	489.14	491.65	494.14
	741.49	744.58	747.67	750.76	753.86	756.98
	1892.41	1899.66	1906.69	1913.52	1920.14	1926.57
NSF	324.76	327.47	330.17	332.86	335.55	338.23
	554.91	557.82	560.70	563.57	566.44	569.30
	1357.86	1363.48	1368.90	1374.12	1379.16	1383.99
F_2NH	473.37	476.96	480.48	483.91	487.28	490.57
	800.58	809.73	818.79	827.75	836.60	845.35

	943.95	950.14	956.26	962.31	968.29	974.21
	1268.84	1273.42	1277.95	1282.43	1286.86	1291.25
	1375.89	1383.83	1391.66	1399.41	1407.06	1414.62
	3040.14	3056.01	3071.71	3087.25	3102.62	3117.83
N_2F_2	348.04	350.31	352.54	354.74	356.91	359.05
	405.46	408.01	410.51	412.95	415.34	417.69
	578.06	581.90	585.68	589.38	593.02	596.60
	930.97	937.79	944.53	951.17	957.74	964.22
	981.37	989.46	997.39	1005.18	1012.83	1020.35
	1527.55	1538.70	1549.75	1560.70	1571.57	1582.33
F_2O	448.65	452.75	456.76	460.69	464.54	468.31
	781.63	792.09	802.53	812.94	823.30	833.59
	949.93	955.97	961.91	967.77	973.56	979.27
F_2SO	301.57	305.04	308.47	311.84	315.17	318.45
	338.88	341.39	343.88	346.34	348.79	351.21
	451.54	455.53	459.48	463.37	467.22	471.03
	621.74	626.00	630.26	634.53	638.79	643.06
	693.16	697.78	702.36	706.91	711.42	715.90
	1257.90	1263.95	1269.87	1275.65	1281.31	1286.84
S_2F_2	572.93	572.63	570.91	569.20	567.53	565.90
	574.37	578.50	584.10	589.72	595.34	600.97
	609.15	614.95	620.71	626.45	632.16	637.83
HNO_3	343.01	346.15	349.49	352.87	356.18	359.36
	559.51	563.82	568.01	572.08	576.03	579.85
	604.90	611.45	617.86	624.10	630.18	636.09
	743.48	747.52	751.51	755.43	759.31	763.13
	849.94	856.21	862.51	868.84	875.20	881.56
	1251.70	1258.99	1266.16	1273.19	1280.05	1286.76
	1300.50	1306.35	1312.24	1318.14	1324.06	1329.97
	1689.02	1695.03	1700.97	1706.86	1712.69	1718.44
	3368.53	3382.56	3396.47	3410.26	3423.94	3437.50
HN_3	522.07	523.07	524.06	525.03	525.98	526.91
	573.94	575.84	577.71	579.56	581.39	583.20
	1126.25	1131.20	1136.07	1140.84	1145.51	1150.05
	1260.11	1263.86	1267.62	1271.38	1275.15	1278.95
	2178.79	2186.01	2193.16	2200.23	2207.24	2214.18
	3230.51	3240.19	3249.82	3259.41	3268.95	3278.45
H_2O	1519.88	1524.59	1529.31	1534.04	1538.78	1543.52
	3632.82	3645.63	3658.35	3670.98	3683.52	3695.98
	3653.39	3665.95	3678.42	3690.81	3703.11	3715.33
H_2O_2	206.86	207.25	207.63	207.99	208.36	208.70
	878.19	884.61	890.97	897.28	903.54	909.75
	1228.94	1235.11	1241.18	1247.15	1253.03	1258.83
	1392.49	1399.04	1405.50	1411.87	1418.16	1424.37
	3441.13	3455.44	3469.65	3483.76	3497.77	3511.68
	3517.16	3531.39	3545.52	3559.56	3573.49	3587.33
H_2S	1155.23	1159.59	1163.95	1168.32	1172.70	1177.08
	2567.67	2575.99	2584.27	2592.51	2600.72	2608.88
	2540.01	2548.13	2556.20	2564.24	2572.24	2580.19
H_2S_2	401.65	400.71	399.78	398.88	398.13	397.39
	458.42	460.39	462.35	464.32	466.29	468.26
	852.34	856.37	860.32	864.21	868.05	871.82
	858.78	862.65	866.46	870.21	873.91	877.53
	2483.91	2493.60	2503.21	2512.76	2522.25	2531.67

	2444.30	2454.00	2463.63	2473.20	2482.71	2492.15
NO_2	743.00	745.63	748.22	750.78	753.29	755.78
	1342.80	1349.90	1356.88	1363.75	1370.52	1377.19
	1613.29	1620.25	1626.95	1633.40	1639.60	1645.55
N_2O	572.56	574.77	576.94	579.10	581.30	583.46
	572.56	574.77	576.94	579.10	581.30	583.46
	1288.11	1291.80	1295.32	1298.68	1301.86	1304.88
	2248.67	2256.60	2264.46	2272.25	2279.97	2287.64
SO_2	484.78	487.95	491.08	494.18	497.24	500.28
	1086.36	1093.76	1101.06	1108.27	1115.40	1122.44
	1251.34	1259.57	1267.70	1275.72	1283.64	1291.46
O_3	715.04	719.51	723.85	728.07	732.17	736.16
	1077.60	1094.43	1110.62	1126.19	1141.14	1155.49
	1193.26	1202.23	1211.05	1219.75	1228.31	1236.75
COClF	399.04	400.35	401.65	402.94	404.22	405.48
	478.39	480.45	482.49	484.51	486.50	488.48
	646.16	648.89	651.61	654.30	656.97	659.62
	735.39	738.52	741.62	744.69	747.73	750.74
	1011.74	1017.71	1023.59	1029.40	1035.13	1040.80
	1859.94	1866.09	1872.20	1878.25	1884.24	1890.19
ClCN	379.32	380.73	382.15	383.58	385.02	386.39
	379.32	380.73	382.15	383.58	385.02	386.39
	721.42	723.34	725.22	727.07	728.89	730.68
	2226.26	2235.33	2244.34	2253.28	2262.16	2270.98
$COCl_2$	426.95	428.96	430.96	432.85	434.80	436.73
	544.64	546.50	548.35	548.79	550.63	552.46
	568.43	570.52	572.60	574.35	576.40	578.45
	788.19	792.34	796.51	798.13	802.25	806.35
	1816.96	1822.83	1828.64	1834.75	1840.46	1846.12
FCN	469.19	471.21	473.26	475.30	477.36	479.42
	469.19	471.21	473.26	475.30	477.36	479.42
	1052.49	1056.26	1059.97	1063.62	1067.21	1070.75
	2324.74	2333.78	2342.75	2351.64	2360.47	2369.22
COF_2	559.62	561.74	563.85	565.92	567.98	570.02
	597.41	600.03	602.61	605.17	607.70	610.19
	746.18	749.71	753.21	756.69	760.14	763.56
	926.64	930.93	935.17	939.34	943.46	947.52
	1141.83	1148.65	1155.38	1162.02	1168.56	1175.01
	1911.60	1917.96	1924.27	1930.51	1936.71	1942.84
CSF_2	409.20	410.64	412.06	413.46	414.85	416.23
	512.16	513.97	515.75	517.51	519.25	520.97
	599.99	602.58	605.14	607.69	610.22	612.73
	769.86	772.85	775.79	778.70	781.56	784.38
	1103.83	1110.76	1117.57	1124.26	1130.85	1137.33
	1311.09	1316.01	1320.86	1325.64	1330.35	1334.99
COS	498.69	500.69	502.71	504.65	506.61	508.55
	498.69	500.69	502.71	504.65	506.61	508.55
	858.35	860.12	861.84	863.49	865.10	866.64
	2048.93	2054.91	2060.82	2066.66	2072.43	2078.14
CO_2	648.99	652.06	655.09	658.11	661.12	664.11
	648.99	652.06	655.09	658.11	661.12	664.11
	1335.98	1341.08	1346.11	1351.10	1356.02	1360.89
	2342.52	2348.29	2353.96	2359.55	2365.05	2370.46
CS_2	388.86	390.31	391.74	393.15	394.52	395.89

	388.86	390.31	391.74	393.15	394.52	395.89
	660.10	662.09	664.05	665.99	667.90	669.78
	1518.17	1520.76	1523.28	1525.71	1528.05	1530.32
HCN	713.20	717.02	721.00	724.81	728.55	732.27
	713.20	717.02	721.00	724.81	728.55	732.27
	2128.67	2136.30	2143.88	2151.40	2158.87	2166.28
	3219.83	3228.72	3237.54	3246.29	3254.98	3263.61
HNCO	591.11	592.09	593.01	593.87	594.70	595.49
	610.83	613.56	616.28	618.98	621.67	624.35
	760.02	762.39	764.79	767.23	769.71	772.23
	1304.33	1308.57	1312.76	1316.89	1320.98	1325.01
	2267.86	2272.86	2277.79	2282.62	2287.38	2292.05
	3428.59	3438.06	3447.45	3456.76	3466.00	3475.16
H_2CO	1144.05	1148.80	1153.54	1158.28	1163.01	1167.75
	1218.64	1222.86	1227.06	1231.24	1235.39	1239.52
	1477.87	1482.82	1487.75	1492.66	1497.56	1502.45
	1760.53	1766.36	1772.16	1777.93	1783.66	1789.35
	2716.42	2726.78	2737.07	2747.29	2757.45	2767.54
	2688.99	2700.74	2712.38	2723.90	2735.30	2746.59
HCOOH	471.86	468.83	465.85	462.93	460.07	457.26
	649.74	652.20	654.62	657.01	659.37	661.70
	984.25	989.23	994.19	999.11	1004.01	1008.88
	1056.37	1060.74	1065.04	1069.26	1073.40	1077.46
	1212.41	1216.95	1221.47	1225.94	1230.39	1234.80
	1362.05	1367.75	1373.42	1379.04	1384.62	1390.17
	1799.68	1805.72	1811.70	1817.63	1823.50	1829.32
	2719.00	2729.41	2739.78	2750.09	2760.35	2770.57
	3436.34	3451.74	3466.93	3481.92	3496.70	3511.29
C_2Cl_2	208.65	218.01	226.93	235.47	243.70	251.61
	208.65	218.01	226.93	235.47	243.70	251.61
	467.83	468.68	469.52	470.35	471.18	472.01
	968.19	969.88	971.57	973.25	974.93	976.61
	2267.79	2275.11	2282.40	2289.64	2296.85	2304.02
C_2N_2	546.66	548.16	549.67	551.21	552.76	554.33
	546.66	548.16	549.68	551.21	552.76	554.33
	871.13	872.40	873.67	874.93	876.18	877.43
	2188.42	2197.59	2206.66	2215.66	2224.56	2233.39
	2359.62	2369.66	2379.61	2389.47	2399.25	2408.95
HCCCl	374.37	373.71	373.27	372.99	372.86	372.88
	374.37	373.71	373.27	372.99	372.86	372.88
	645.74	649.22	654.39	657.47	659.77	663.66
	645.74	649.23	654.39	657.47	659.77	663.66
	733.43	734.75	736.06	737.37	738.67	739.97
	2119.42	2126.19	2132.91	2139.57	2146.19	2152.77
	3230.21	3238.47	3246.72	3254.86	3262.91	3270.96
HCCF	384.44	387.68	390.91	394.11	397.30	400.46
	384.44	387.68	390.91	394.11	397.30	400.46
	553.57	558.28	563.05	567.83	572.63	577.45
	553.57	558.28	563.05	567.83	572.63	577.45
	1046.51	1049.17	1051.82	1054.45	1057.05	1059.63
	2239.90	2246.69	2253.44	2260.13	2266.78	2273.38
	3243.76	3251.92	3260.02	3268.08	3276.10	3284.08
HCCH	765.83	764.87	764.18	763.74	763.54	763.56
	765.83	764.87	764.18	763.74	763.54	763.56

	788.48	791.07	793.70	796.35	799.02	801.71
	788.48	791.07	793.70	796.35	799.02	801.72
	1998.86	2004.65	2010.41	2016.12	2021.80	2027.44
	3184.54	3192.51	3200.42	3208.29	3216.13	3223.92
	3333.26	3341.44	3349.56	3357.65	3365.69	3373.68
trans-CHClCHCl	342.10	343.02	343.93	344.83	345.72	346.61
	779.67	783.75	787.79	791.81	795.79	799.73
	778.78	780.94	783.09	785.21	787.31	789.40
	821.75	824.37	826.96	829.53	832.08	834.60
	905.15	909.45	913.72	917.97	922.20	926.40
	1188.55	1193.12	1197.65	1202.16	1206.64	1211.10
	1265.21	1269.91	1274.59	1279.24	1283.87	1288.47
	1585.68	1591.36	1597.00	1602.62	1608.20	1613.75
	2993.92	3002.48	3010.99	3019.46	3027.89	3036.28
	3047.82	3056.36	3064.86	3073.32	3081.74	3090.12
cis-CHClCHCl	410.82	412.09	413.36	414.62	415.87	417.12
	556.63	558.04	559.44	560.82	562.20	563.57
	718.16	721.26	690.41	692.09	693.75	695.40
	687.01	688.72	724.36	727.44	730.51	733.56
	819.25	821.78	824.29	826.78	829.24	831.68
	892.11	897.14	902.13	907.09	912.02	916.91
	1185.49	1189.85	1194.19	1198.51	1202.81	1207.09
	1274.04	1279.00	1283.94	1288.85	1293.73	1298.59
	1586.78	1592.41	1598.00	1603.57	1609.11	1614.62
	2967.73	2976.17	2984.56	2992.92	3001.24	3009.52
	3041.21	3049.64	3058.02	3066.37	3074.68	3082.95
CH_2CCl_2	298.15	298.84	299.53	298.31	299.00	299.68
	385.47	386.48	387.48	388.56	389.55	390.53
	472.24	473.44	474.64	475.66	476.85	478.03
	582.44	584.05	585.65	586.50	588.08	589.64
	701.33	704.02	706.71	709.35	712.01	714.67
	741.98	745.35	748.69	750.62	753.92	757.19
	908.48	913.23	917.97	922.94	927.65	932.35
	1066.37	1070.59	1074.78	1078.52	1082.67	1086.81
	1359.61	1364.61	1369.60	1374.63	1379.58	1384.52
	1601.35	1606.91	1612.45	1617.89	1623.38	1628.85
	2993.23	3001.02	3008.79	3016.51	3024.21	3031.89
	3020.27	3028.25	3036.20	3044.07	3051.94	3059.77
cis-CHFCHF	497.98	499.74	501.50	503.24	504.96	506.67
	777.43	781.09	784.73	758.74	760.94	763.12
	752.05	754.30	756.53	788.34	791.94	795.51
	860.64	865.80	870.92	876.00	881.03	886.02
	987.96	991.09	994.18	997.24	1000.26	1003.25
	1088.29	1091.64	1094.96	1098.24	1101.49	1104.71
	1245.44	1249.83	1254.21	1258.56	1262.88	1267.18
	1337.59	1342.75	1347.88	1352.98	1358.04	1363.08
	1689.75	1696.07	1702.35	1708.58	1714.77	1720.92
	2983.98	2993.15	3002.25	3011.30	3020.29	3029.22
	3058.87	3068.00	3077.07	3086.09	3095.06	3103.96
trans-CHFCHF	315.53	316.74	317.94	319.13	320.30	321.47
	323.46	324.74	326.01	327.27	328.53	329.77
	539.31	540.97	542.61	544.23	545.84	547.44
	804.14	808.79	813.39	817.95	822.48	826.96
	879.75	884.20	888.62	893.02	897.40	901.75

	1099.03	1102.64	1106.21	1109.73	1113.22	1116.66
	1109.62	1112.95	1116.25	1119.50	1122.73	1125.91
	1248.64	1253.45	1258.24	1263.00	1267.73	1272.43
	1266.92	1271.53	1276.11	1280.67	1285.21	1289.73
	1677.52	1683.90	1690.24	1696.52	1702.76	1708.95
	2994.21	3003.26	3012.25	3021.20	3030.09	3038.93
	3051.60	3060.61	3069.57	3078.48	3087.34	3096.16
OCHCHO	337.69	339.17	340.65	342.11	343.57	345.01
	533.81	535.88	537.93	539.96	541.98	543.97
	777.99	782.10	786.16	790.16	794.11	797.99
	1009.59	1013.76	1017.91	1022.04	1026.13	1030.20
	1041.64	1046.14	1050.62	1055.06	1059.49	1063.89
	1289.89	1295.00	1300.08	1305.13	1310.15	1315.14
	1331.34	1336.35	1341.33	1346.29	1351.23	1356.14
	1743.76	1751.25	1758.68	1760.09	1766.30	1772.45
	1741.08	1747.49	1753.82	1766.03	1773.31	1780.53
	2690.49	2701.88	2713.17	2724.37	2735.47	2746.47
	2750.89	2761.92	2772.87	2783.73	2794.50	2805.18
CH_2CH_2	833.10	835.47	837.83	840.19	842.54	844.88
	949.25	954.88	958.69	962.50	966.30	970.09
	951.06	953.54	957.81	962.06	966.29	970.51
	1029.42	1032.48	1035.53	1038.57	1041.61	1044.64
	1206.59	1210.58	1214.56	1218.54	1222.51	1226.47
	1342.81	1346.71	1350.60	1354.47	1358.34	1362.18
	1424.90	1429.73	1434.56	1439.38	1444.19	1449.00
	1630.36	1635.36	1640.35	1645.31	1650.26	1655.19
	2961.69	2969.48	2977.24	2984.97	2992.66	3000.32
	2999.95	3007.86	3015.74	3023.59	3031.42	3039.20
	3016.60	3024.60	3032.56	3040.49	3048.37	3056.22
	3047.30	3055.24	3063.16	3071.03	3078.87	3086.67
cyclo-C_2H_4O	812.48	815.18	817.85	820.49	823.12	825.72
	798.61	802.31	805.94	809.53	813.07	816.55
	855.73	858.48	861.20	863.90	866.58	869.24
	1015.02	1018.98	1022.92	1026.83	1030.73	1034.61
	1115.60	1120.51	1125.40	1130.27	1135.13	1139.97
	1128.12	1132.94	1137.75	1142.55	1147.34	1152.11
	1135.70	1139.88	1144.04	1148.17	1152.29	1156.38
	1145.08	1149.06	1153.03	1156.99	1160.93	1164.86
	1262.85	1266.83	1270.80	1274.75	1278.68	1282.60
	1452.14	1457.29	1462.43	1467.56	1472.68	1477.79
	1484.54	1490.04	1495.52	1500.99	1506.44	1511.87
	2913.78	2922.32	2930.81	2939.25	2947.63	2955.96
	2943.90	2952.52	2961.08	2969.60	2978.06	2986.46
	2977.31	2986.23	2995.08	3003.86	3012.56	3021.20
	2994.98	3003.90	3012.75	3021.54	3030.25	3038.90
cyclo-C_2H_4NH	779.85	782.75	785.61	788.43	791.21	793.95
	815.33	818.62	821.86	825.06	828.21	831.33
	847.03	849.68	852.30	854.90	857.48	860.02
	915.02	918.14	921.25	924.34	927.41	930.47
	996.57	999.61	1002.62	1005.60	1008.55	1011.49
	1085.84	1090.08	1094.31	1098.54	1102.76	1106.98
	1089.35	1093.98	1098.59	1103.19	1107.78	1112.36
	1117.55	1121.56	1125.56	1129.56	1133.54	1137.52
	1203.10	1207.35	1211.57	1215.76	1219.92	1224.05

	1230.91	1235.09	1239.26	1243.42	1247.57	1251.71
	1256.44	1260.02	1263.59	1267.14	1270.68	1274.21
	1447.27	1452.32	1457.37	1462.40	1467.43	1472.45
	1473.70	1479.06	1484.40	1489.73	1495.04	1500.34
	2926.96	2934.94	2942.87	2950.75	2958.59	2966.39
	2957.23	2965.28	2973.29	2981.25	2989.18	2997.06
	2989.03	2997.26	3005.44	3013.56	3021.63	3029.65
	3007.23	3015.49	3023.69	3031.84	3039.94	3047.99
	3212.33	3223.89	3235.36	3246.73	3258.02	3269.21
CH_2CCHCl	349.42	350.87	352.30	353.73	355.13	356.53
	492.27	493.97	495.65	497.32	498.97	500.61
	597.64	599.35	601.06	602.76	604.45	606.14
	726.11	728.86	731.59	734.28	736.95	739.59
	846.20	849.69	853.16	856.62	860.05	863.46
	913.96	918.19	922.41	926.62	930.82	935.01
	998.01	1001.61	1005.20	1008.78	1012.35	1015.91
	1105.54	1108.67	1111.78	1114.87	1117.95	1121.01
	1234.50	1238.91	1243.31	1247.69	1252.05	1256.41
	1420.75	1425.75	1430.74	1435.72	1440.69	1445.65
	1982.25	1987.65	1993.01	1998.36	2003.67	2008.96
	2936.48	2944.83	2953.13	2961.39	2969.60	2977.78
	2936.25	2945.21	2954.11	2962.95	2971.72	2980.45
	2964.40	2972.96	2981.48	2989.96	2998.41	3006.82
CH_2CHCHO	339.53	340.41	341.27	342.14	343.00	343.85
	565.68	567.03	568.38	569.71	571.04	572.35
	620.15	622.03	623.90	625.77	627.62	629.47
	913.49	916.02	918.53	921.04	923.53	926.01
	983.50	987.69	991.87	996.04	1000.20	1004.34
	1001.15	1005.40	1009.63	1013.83	1017.99	1022.11
	1013.94	1017.40	1020.82	1024.19	1027.51	1030.77
	1138.24	1141.70	1145.16	1148.60	1152.03	1155.45
	1263.87	1268.14	1272.40	1276.64	1280.87	1285.08
	1342.65	1347.61	1352.54	1357.44	1362.31	1367.16
	1413.16	1418.17	1423.16	1428.13	1433.08	1438.01
	1618.97	1624.45	1629.90	1635.30	1640.67	1646.00
	1707.07	1713.98	1720.84	1727.63	1734.37	1741.05
	2627.50	2638.95	2650.30	2661.55	2672.71	2683.77
	2950.77	2959.16	2967.49	2975.77	2983.99	2992.15
	2943.39	2951.96	2960.47	2968.94	2977.36	2985.73
	2992.82	3000.92	3008.97	3016.98	3024.95	3032.88
cyclo-C_3H_6	763.41	765.69	767.96	770.22	772.46	774.68
	757.97	760.35	762.72	765.08	767.41	769.73
	869.16	871.49	873.80	876.10	878.40	880.68
	846.71	848.82	850.92	853.00	855.06	857.10
	846.72	848.83	850.93	853.01	855.07	857.11
	1038.16	1042.23	1046.29	1050.35	1054.40	1058.45
	1032.26	1036.41	1040.56	1044.70	1048.84	1052.97
	1068.95	1073.35	1077.74	1082.12	1086.49	1090.84
	1119.95	1123.80	1127.65	1131.49	1135.33	1139.17
	1180.80	1184.75	1188.69	1192.62	1196.54	1200.45
	1180.55	1184.51	1188.47	1192.41	1196.34	1200.26
	1183.82	1186.47	1189.11	1191.73	1194.34	1196.94
	1428.18	1433.09	1437.99	1442.89	1447.78	1452.67
	1426.45	1431.41	1436.36	1441.30	1446.24	1451.16

	1471.75	1477.05	1482.33	1487.60	1492.86	1498.10
	2954.27	2961.71	2969.11	2976.47	2983.81	2991.10
	2963.01	2970.42	2977.78	2985.12	2992.41	2999.69
	2993.60	3001.14	3008.64	3016.11	3023.54	3030.94
	3012.96	3020.58	3028.16	3035.69	3043.18	3050.63
	3013.48	3021.09	3028.65	3036.17	3043.65	3051.11
	3055.94	3063.53	3071.08	3078.59	3086.06	3093.49
$CH_2CHCHCH_2$	519.20	520.38	521.55	522.71	523.86	525.00
	584.71	586.21	587.69	589.16	590.62	592.07
	787.18	789.63	792.05	794.47	796.86	799.25
	891.24	893.19	895.13	897.06	898.98	900.90
	940.93	945.13	949.30	953.45	957.58	961.69
	943.14	947.43	951.70	955.94	960.16	964.37
	983.27	987.35	991.40	995.43	999.45	1003.44
	993.94	997.21	1000.46	1003.69	1006.91	1010.11
	1036.86	1040.49	1044.10	1047.68	1051.26	1054.82
	1194.49	1197.75	1201.00	1204.25	1207.49	1210.74
	1279.25	1283.51	1287.75	1291.98	1296.18	1300.37
	1282.67	1287.11	1291.53	1295.94	1300.32	1304.69
	1373.81	1378.42	1383.01	1387.58	1392.13	1396.66
	1427.81	1432.71	1437.61	1442.49	1447.36	1452.23
	1594.16	1599.37	1604.54	1609.69	1614.80	1619.89
	1642.65	1648.48	1654.28	1660.05	1665.79	1671.50
	2949.62	2958.48	2967.31	2976.13	2984.92	2993.70
	2970.82	2984.04	2993.30	3000.40	3006.71	3012.91
	2972.55	2997.67	3006.27	3014.85	3023.42	3031.95
	2989.09	2974.74	2975.20	2975.91	2977.79	2980.95
	3038.91	3046.79	3054.63	3062.44	3070.20	3077.93
	3034.33	3042.37	3050.38	3058.34	3066.26	3074.14
$^1CH_2$	1323.16	1327.44	1331.72	1336.02	1340.32	1344.63
	2756.95	2767.27	2777.51	2787.67	2797.75	2807.76
	2760.53	2770.63	2780.64	2790.57	2800.41	2810.18
$^3CH_2$	907.12	913.92	920.67	927.37	934.02	940.62
	2996.60	3004.15	3011.67	3019.16	3026.62	3034.06
	3183.44	3190.68	3197.90	3205.08	3212.23	3219.35
HCO	1049.04	1052.66	1056.28	1059.89	1063.50	1067.10
	1876.18	1882.93	1889.62	1896.23	1902.77	1909.24
	2333.20	2344.15	2355.02	2365.80	2376.49	2387.11

Table S7. TOSH 2MR vibrational frequencies in cm^{-1} for the modified F1 anharmonic test set calculated using the 6-311++G(d,p) basis set and modified KS-B3LYP functional with different amounts of exact Hartree-Fock exchange parameterized between 22-32%.

Molecule	22% HF	24% HF	26% HF	28% HF	30% HF	32% HF
$NClF_2$	353.14	355.61	358.02	360.38	362.70	364.96
	360.04	363.62	367.13	370.57	373.95	377.25
	556.52	560.10	563.57	566.94	570.21	573.40
	677.61	682.59	687.49	692.34	697.14	701.89
	820.18	830.04	839.79	849.43	858.95	868.37
	935.30	941.67	948.02	954.36	960.67	966.96
ClF_3	376.39	380.48	384.44	388.27	391.98	395.58
	479.66	482.67	485.68	488.70	491.72	494.74
	661.49	662.93	664.33	665.69	667.01	668.30
	676.59	682.79	688.84	694.76	700.57	706.26
HOCl	697.05	700.57	704.07	707.54	710.99	714.42
	1205.15	1209.94	1214.69	1219.40	1224.07	1228.70
	3563.71	3577.35	3590.90	3604.36	3617.73	3631.02
ClNO	322.30	322.78	323.20	323.56	323.85	324.09
	596.76	598.58	600.37	602.15	603.90	605.64
	1904.54	1911.99	1919.26	1926.32	1933.19	1939.87
$ClNO_2$	362.42	365.21	368.09	371.06	374.12	377.28
	408.94	411.49	414.03	416.55	419.05	421.54
	662.78	666.18	669.55	672.89	676.20	679.49
	804.84	808.09	811.35	814.61	817.87	821.15
	1337.50	1341.71	1345.86	1349.96	1354.02	1358.05
	1726.75	1729.62	1732.35	1734.93	1737.39	1739.73
ClSN	385.08	386.65	388.21	389.78	391.36	392.94
	1350.44	1355.14	1359.61	1363.85	1367.85	1371.62
NCl_2F	333.29	336.44	339.51	342.49	345.39	348.22
	419.61	422.73	425.77	428.72	431.58	434.35
	607.25	610.19	613.07	615.89	618.68	621.42
	646.55	654.22	661.84	669.41	676.92	684.38
	819.06	827.18	835.28	843.35	851.38	859.35
Cl_2O	617.22	633.19	635.97	638.75	641.50	644.25
	630.38	626.08	634.78	643.31	651.68	659.90
$SOCl_2$	316.17	318.39	320.59	322.78	324.95	327.10
	411.96	414.54	417.13	419.73	422.35	424.99
	455.71	458.23	460.74	463.23	465.71	468.17
	1214.22	1218.97	1223.56	1228.01	1232.31	1236.47
SCl_2	466.22	468.90	471.56	474.21	476.86	479.49
	485.09	487.08	489.05	491.01	492.94	494.86
HOF	931.49	937.19	942.85	948.47	954.04	959.56
	1364.07	1370.85	1377.55	1384.18	1390.76	1397.26
	3520.97	3534.99	3548.92	3562.75	3576.50	3590.15
ONF	496.60	499.06	501.52	503.98	506.47	508.99
	760.12	763.28	766.48	769.72	772.99	776.30
	1932.81	1938.87	1944.75	1950.46	1956.02	1961.43
NSF	340.91	343.58	346.26	348.92	351.58	354.24
	572.17	575.05	577.94	580.85	583.77	586.71
	1388.64	1393.09	1397.34	1401.40	1405.26	1408.93
F_2NH	493.80	496.96	500.06	503.10	506.08	509.01
	853.98	862.50	870.91	879.20	887.38	895.44

	980.07	985.87	991.61	997.29	1002.92	1008.48
	1295.61	1299.93	1304.23	1308.49	1312.73	1316.94
	1422.10	1429.49	1436.80	1444.04	1451.19	1458.28
	3132.88	3147.77	3162.51	3177.11	3191.55	3205.85
N_2F_2	361.16	363.23	365.28	367.31	369.30	371.27
	419.99	422.25	424.46	426.63	428.77	430.87
	600.12	603.57	606.97	610.31	613.60	616.84
	970.62	976.95	983.20	989.38	995.48	1001.51
	1027.75	1035.02	1042.18	1049.23	1056.18	1063.03
	1593.00	1603.57	1614.04	1624.42	1634.70	1644.89
F_2O	472.01	475.64	479.21	482.71	486.16	489.55
	843.81	853.95	863.99	873.94	883.79	893.53
	984.92	990.51	996.06	1001.55	1007.00	1012.40
F_2SO	321.69	324.87	328.01	331.10	334.14	337.14
	353.61	355.99	358.36	360.70	363.02	365.33
	474.79	478.50	482.17	485.80	489.38	492.92
	647.32	651.58	655.83	660.08	664.32	668.55
	720.34	724.75	729.14	733.49	737.81	742.10
	1292.25	1297.54	1302.72	1307.79	1312.74	1317.60
S_2F_2	564.32	562.80	560.52	559.13	557.83	556.60
	606.60	612.21	617.24	622.80	628.32	633.80
	643.45	649.03	653.91	659.38	664.79	670.14
HNO_3	362.38	365.22	367.87	370.36	372.67	374.82
	583.56	587.16	590.67	594.10	597.45	600.74
	641.83	647.40	652.81	658.05	663.14	668.07
	766.91	770.64	774.33	777.98	781.59	785.16
	887.94	894.33	900.72	907.09	913.46	919.81
	1293.30	1299.67	1305.90	1311.99	1317.95	1323.78
	1335.89	1341.80	1347.71	1353.63	1359.55	1365.47
	1724.12	1729.73	1735.26	1740.72	1746.10	1751.39
	3450.95	3464.28	3477.52	3490.65	3503.68	3516.62
HN_3	527.82	528.72	529.59	530.45	531.30	532.13
	584.99	586.76	588.51	590.24	591.95	593.64
	1154.45	1158.71	1162.79	1166.69	1170.38	1173.86
	1282.77	1286.62	1290.50	1294.42	1298.38	1302.37
	2221.05	2227.86	2234.60	2241.28	2247.89	2254.44
	3287.91	3297.33	3306.72	3316.06	3325.37	3334.64
H_2O	1548.28	1553.04	1557.80	1562.58	1567.36	1572.14
	3708.36	3720.65	3732.86	3744.99	3757.05	3769.03
	3727.46	3739.51	3751.48	3763.37	3775.18	3786.91
H_2O_2	209.03	209.34	209.62	209.88	210.12	210.33
	915.91	922.01	928.06	934.07	940.02	945.93
	1264.54	1270.17	1275.73	1281.21	1286.63	1291.97
	1430.51	1436.58	1442.58	1448.51	1454.39	1460.20
	3525.49	3539.21	3552.83	3566.35	3579.79	3593.13
	3601.07	3614.72	3628.27	3641.74	3655.11	3668.39
H_2S	1181.47	1185.86	1190.25	1194.66	1199.07	1203.48
	2617.00	2625.07	2633.11	2641.11	2649.08	2657.01
	2588.11	2595.97	2603.80	2611.60	2619.36	2627.09
H_2S_2	396.75	396.29	395.91	395.54	395.18	394.83
	470.22	472.18	474.14	476.09	478.03	479.97
	875.54	879.22	882.87	886.46	890.01	893.51
	881.09	884.61	888.08	891.49	894.84	898.16
	2541.03	2550.34	2559.59	2568.79	2577.92	2587.00

	2501.53	2510.87	2520.16	2529.38	2538.55	2547.68
NO_2	758.22	760.64	763.02	765.38	767.70	769.99
	1383.75	1390.21	1396.57	1402.83	1409.00	1415.07
	1651.26	1656.73	1661.97	1666.97	1671.75	1676.30
N_2O	585.59	587.67	589.78	591.88	593.92	595.96
	585.59	587.67	589.78	591.88	593.92	595.96
	1307.72	1310.39	1312.89	1315.21	1317.35	1319.32
	2295.24	2302.80	2310.30	2317.75	2325.16	2332.52
SO_2	503.28	506.25	509.19	512.10	514.98	517.83
	1129.39	1136.26	1143.05	1149.76	1156.40	1162.96
	1299.18	1306.80	1314.32	1321.75	1329.09	1336.34
O_3	740.05	743.83	747.52	751.12	754.64	758.07
	1169.24	1182.41	1194.99	1207.01	1218.45	1229.33
	1245.07	1253.28	1261.37	1269.35	1277.23	1285.00
COClF	406.73	407.96	409.19	410.40	411.60	412.80
	490.43	492.37	494.29	496.18	498.06	499.92
	662.25	664.86	667.46	670.04	672.60	675.14
	753.72	756.67	759.59	762.49	765.36	768.21
	1046.39	1051.92	1057.39	1062.79	1068.12	1073.40
	1896.09	1901.94	1907.74	1913.49	1919.19	1924.84
ClCN	387.88	389.32	390.76	392.19	393.62	395.10
	387.89	389.32	390.76	392.19	393.62	395.10
	732.45	734.18	735.90	737.58	739.25	740.89
	2279.74	2288.43	2297.06	2305.64	2314.15	2322.60
$COCl_2$	438.65	440.54	442.41	444.27	446.10	447.92
	554.28	556.10	557.91	559.71	561.51	563.30
	580.47	582.49	584.49	586.48	588.46	590.43
	810.41	814.46	818.49	822.50	826.49	830.44
	1851.74	1857.30	1862.82	1868.29	1873.71	1879.09
FCN	481.49	483.56	485.64	487.73	489.83	491.95
	481.49	483.56	485.64	487.73	489.83	491.95
	1074.24	1077.68	1081.08	1084.43	1087.74	1091.01
	2377.91	2386.53	2395.08	2403.56	2411.98	2420.34
COF_2	572.04	574.03	576.01	577.97	579.92	581.84
	612.66	615.10	617.52	619.90	622.26	624.60
	766.97	770.34	773.70	777.03	780.34	783.63
	951.53	955.49	959.40	963.26	967.07	970.84
	1181.38	1187.66	1193.86	1199.97	1206.00	1211.96
	1948.92	1954.95	1960.92	1966.83	1972.69	1978.50
CSF_2	417.58	418.93	420.25	421.57	422.87	424.15
	522.67	524.35	526.01	527.65	529.28	530.88
	615.22	617.69	620.15	622.59	625.01	627.41
	787.16	789.90	792.61	795.27	797.91	800.51
	1143.71	1150.00	1156.18	1162.28	1168.28	1174.20
	1339.55	1344.05	1348.47	1352.83	1357.11	1361.32
COS	510.48	512.40	514.31	516.20	518.08	519.94
	510.48	512.40	514.31	516.20	518.08	519.95
	868.13	869.57	870.95	872.28	873.55	874.77
	2083.77	2089.34	2094.86	2100.30	2105.69	2111.01
CO_2	667.09	670.06	673.01	675.95	678.87	681.79
	667.09	670.06	673.01	675.95	678.87	681.79
	1365.71	1370.48	1375.19	1379.85	1384.47	1389.03
	2375.79	2381.04	2386.20	2391.28	2396.28	2401.21
CS_2	397.23	398.56	399.88	401.19	402.49	403.78

	397.23	398.56	399.88	401.19	402.49	403.78
	671.64	673.48	675.28	677.07	678.83	680.56
	1532.50	1534.60	1536.62	1538.56	1540.42	1542.20
HCN	735.98	739.70	743.45	747.22	751.11	755.35
	735.98	739.70	743.45	747.22	751.11	755.35
	2173.63	2180.94	2188.19	2195.39	2202.54	2209.64
	3272.19	3280.70	3289.17	3297.58	3305.94	3314.28
HNCO	596.24	596.96	597.65	598.33	598.99	599.64
	627.01	629.66	632.30	634.92	637.53	640.13
	774.78	777.38	780.01	782.68	785.39	788.14
	1329.00	1332.93	1336.82	1340.66	1344.45	1348.19
	2296.64	2301.15	2305.59	2309.94	2314.23	2318.44
	3484.25	3493.27	3502.22	3511.10	3519.91	3528.65
H_2CO	1172.47	1177.20	1181.92	1186.63	1191.35	1196.06
	1243.63	1247.71	1251.77	1255.82	1259.84	1263.84
	1507.33	1512.19	1517.03	1521.87	1526.69	1531.49
	1795.01	1800.64	1806.24	1811.80	1817.33	1822.82
	2777.59	2787.57	2797.48	2807.34	2817.14	2826.88
	2757.79	2768.88	2779.86	2790.73	2801.51	2812.19
HCOOH	454.51	451.81	449.17	446.58	444.04	441.55
	664.01	666.28	668.53	670.75	672.95	675.12
	1013.72	1018.54	1023.32	1028.08	1032.82	1037.53
	1081.46	1085.39	1089.25	1093.05	1096.79	1100.47
	1239.19	1243.55	1247.87	1252.18	1256.45	1260.71
	1395.68	1401.16	1406.60	1412.01	1417.38	1422.73
	1835.09	1840.81	1846.47	1852.09	1857.66	1863.18
	2780.73	2790.85	2800.91	2810.93	2820.90	2830.81
	3525.69	3539.92	3553.97	3567.86	3581.59	3595.17
C_2Cl_2	259.25	266.64	273.81	280.77	287.54	294.12
	259.25	266.64	273.81	280.77	287.54	294.12
	472.83	473.65	474.47	475.28	476.09	476.89
	978.28	979.95	981.62	983.28	984.94	986.60
	2311.16	2318.25	2325.31	2332.33	2339.32	2346.27
C_2N_2	555.90	557.51	559.11	560.74	562.37	564.03
	555.90	557.51	559.11	560.74	562.37	564.03
	878.68	879.93	881.17	882.41	883.65	884.88
	2242.14	2250.81	2259.40	2267.92	2276.36	2284.73
	2418.56	2428.10	2437.55	2446.93	2456.22	2465.44
HCCCl	373.05	373.32	373.68	374.14	374.67	375.27
	373.05	373.32	373.68	374.14	374.67	375.27
	667.88	671.73	675.42	679.21	682.79	686.29
	667.88	671.73	675.42	679.21	682.79	686.29
	741.26	742.54	743.82	745.10	746.37	747.64
	2159.30	2165.79	2172.24	2178.64	2185.00	2191.33
	3278.98	3286.94	3294.84	3302.71	3310.53	3318.30
HCCF	403.59	406.70	409.79	412.85	415.89	418.87
	403.60	406.70	409.79	412.85	415.89	418.87
	582.28	587.12	591.96	596.79	600.80	605.53
	582.28	587.12	591.96	596.79	600.80	605.53
	1062.20	1064.75	1067.28	1069.79	1072.29	1074.77
	2279.94	2286.46	2292.93	2299.36	2305.75	2312.09
	3292.01	3299.90	3307.74	3315.55	3323.29	3331.02
HCCH	763.77	764.18	764.76	765.48	766.33	766.73
	763.77	764.18	764.76	765.48	766.33	766.73

	804.43	807.17	809.92	812.69	815.46	817.61
	804.43	807.17	809.92	812.69	815.46	817.61
	2033.04	2038.61	2044.14	2049.64	2055.11	2060.54
	3231.67	3239.39	3247.06	3254.71	3262.31	3269.84
	3381.64	3389.55	3397.43	3405.27	3413.06	3420.78
trans-CHClCHCl	347.50	348.37	349.24	350.11	350.97	351.82
	803.65	807.54	811.40	815.23	819.03	822.80
	791.47	793.52	795.55	797.57	799.57	801.56
	837.10	839.58	842.04	844.48	846.90	849.30
	930.59	934.76	938.90	943.03	947.14	951.24
	1215.53	1219.94	1224.31	1228.68	1233.02	1237.34
	1293.06	1297.62	1302.16	1306.68	1311.18	1315.66
	1619.26	1624.74	1630.19	1635.61	1641.00	1646.35
	3044.64	3052.96	3061.24	3069.48	3077.69	3085.87
	3098.47	3106.78	3115.06	3123.31	3131.51	3139.69
cis-CHClCHCl	418.36	419.59	420.82	422.03	423.24	424.45
	564.93	566.29	567.63	568.97	570.30	571.63
	697.04	698.66	700.27	701.87	703.45	705.02
	736.60	739.63	742.64	745.64	748.63	751.61
	834.10	836.51	838.89	841.25	843.60	845.92
	921.77	926.60	931.39	936.16	940.89	945.59
	1211.35	1215.60	1219.82	1224.03	1228.22	1232.39
	1303.43	1308.25	1313.05	1317.83	1322.58	1327.32
	1620.10	1625.56	1630.98	1636.38	1641.75	1647.10
	3017.76	3025.96	3034.13	3042.26	3050.36	3058.42
	3091.19	3099.39	3107.56	3115.69	3123.79	3131.86
CH_2CCl_2	300.36	301.03	301.69	302.35	303.01	303.66
	391.50	392.47	393.43	394.39	395.33	396.28
	479.21	480.38	481.54	482.70	483.87	485.02
	591.18	592.72	594.24	595.74	597.24	598.72
	717.31	719.94	722.56	725.18	727.78	730.37
	760.44	763.66	766.85	770.03	773.17	776.29
	937.04	941.72	946.39	951.05	955.70	960.33
	1090.92	1095.02	1099.10	1103.16	1107.20	1111.22
	1389.44	1394.34	1399.22	1404.09	1408.95	1413.79
	1634.29	1639.72	1645.12	1650.49	1655.85	1661.18
	3039.53	3047.14	3054.73	3062.28	3069.81	3077.30
	3067.57	3075.33	3083.06	3090.75	3098.40	3106.02
cis-CHFCHF	508.37	510.06	511.73	513.39	515.04	516.68
	765.29	767.45	769.59	771.71	773.83	775.93
	799.05	802.58	806.08	809.57	813.03	816.47
	890.96	895.87	900.73	905.55	910.33	915.07
	1006.21	1009.13	1012.03	1014.89	1017.73	1020.53
	1107.89	1111.05	1114.18	1117.27	1120.34	1123.38
	1271.46	1275.72	1279.95	1284.16	1288.36	1292.53
	1368.08	1373.04	1377.98	1382.89	1387.77	1392.62
	1727.02	1733.08	1739.10	1745.08	1751.02	1756.92
	3038.10	3046.92	3055.69	3064.41	3073.07	3081.68
	3112.82	3121.62	3130.37	3139.07	3147.72	3156.32
trans-CHFCHF	322.62	323.75	324.88	326.00	327.10	328.20
	331.01	332.24	333.46	334.67	335.87	337.06
	549.03	550.61	552.17	553.72	555.26	556.79
	831.41	835.82	840.19	844.53	848.83	853.10
	906.08	910.39	914.67	918.94	923.19	927.41

	1120.07	1123.44	1126.78	1130.08	1133.35	1136.59
	1129.06	1132.18	1135.26	1138.31	1141.33	1144.32
	1277.11	1281.77	1286.40	1291.00	1295.59	1300.15
	1294.23	1298.71	1303.17	1307.60	1312.03	1316.43
	1715.09	1721.19	1727.24	1733.24	1739.20	1745.12
	3047.73	3056.47	3065.17	3073.82	3082.42	3090.98
	3104.93	3113.65	3122.32	3130.95	3139.54	3148.08
OCHCHO	346.44	347.85	349.26	350.66	352.04	353.41
	545.95	547.90	549.84	551.76	553.66	555.54
	801.84	805.64	809.39	813.11	816.78	820.41
	1034.24	1038.25	1042.23	1046.18	1050.12	1054.02
	1068.26	1072.62	1076.95	1081.26	1085.55	1089.82
	1320.09	1325.02	1329.92	1334.79	1339.63	1344.45
	1361.02	1365.89	1370.73	1375.55	1380.35	1385.13
	1778.53	1784.56	1790.53	1796.44	1802.30	1808.10
	1787.69	1794.78	1801.81	1808.79	1815.70	1822.56
	2757.39	2768.21	2778.95	2789.60	2800.16	2810.64
	2815.78	2826.30	2836.74	2847.10	2857.38	2867.59
CH_2CH_2	847.22	849.55	851.88	854.20	856.51	858.82
	973.89	977.67	981.45	985.22	988.99	992.75
	974.71	978.89	983.05	987.20	991.33	995.44
	1047.66	1050.68	1053.69	1056.70	1059.69	1062.68
	1230.42	1234.37	1238.30	1242.24	1246.16	1250.08
	1366.02	1369.84	1373.66	1377.46	1381.24	1385.02
	1453.81	1458.60	1463.40	1468.19	1472.97	1477.75
	1660.11	1665.01	1669.90	1674.77	1679.63	1684.47
	3007.95	3015.55	3023.12	3030.66	3038.16	3045.64
	3046.96	3054.69	3062.39	3070.05	3077.69	3085.29
	3064.03	3071.80	3079.54	3087.23	3094.89	3102.51
	3094.43	3102.16	3109.85	3117.51	3125.13	3132.72
cyclo-C_2H_4O	828.30	830.86	833.40	835.92	838.42	840.90
	819.99	823.37	826.71	830.01	833.25	836.46
	871.87	874.48	877.07	879.64	882.19	884.73
	1038.47	1042.31	1046.14	1049.95	1053.74	1057.51
	1144.79	1149.60	1154.40	1159.18	1163.94	1168.68
	1156.88	1161.63	1166.37	1171.10	1175.82	1180.53
	1160.45	1164.50	1168.53	1172.54	1176.53	1180.50
	1168.78	1172.69	1176.59	1180.48	1184.35	1188.22
	1286.49	1290.37	1294.24	1298.08	1301.91	1305.73
	1482.89	1487.98	1493.06	1498.13	1503.19	1508.24
	1517.29	1522.70	1528.09	1533.47	1538.83	1544.18
	2964.24	2972.48	2980.66	2988.79	2996.88	3004.92
	2994.82	3003.14	3011.40	3019.62	3027.79	3035.92
	3029.77	3038.28	3046.73	3055.11	3063.43	3071.70
	3047.48	3056.00	3064.46	3072.86	3081.20	3089.48
cyclo-C_2H_4NH	796.67	799.35	802.00	804.62	807.22	809.79
	834.41	837.44	840.44	843.40	846.32	849.21
	862.55	865.06	867.54	870.01	872.45	874.88
	933.51	936.55	939.57	942.57	945.57	948.56
	1014.40	1017.29	1020.17	1023.03	1025.87	1028.70
	1111.20	1115.42	1119.63	1123.84	1128.05	1132.26
	1116.92	1121.47	1126.01	1130.54	1135.05	1139.55
	1141.50	1145.46	1149.42	1153.37	1157.32	1161.26
	1228.16	1232.24	1236.29	1240.31	1244.31	1248.28

	1255.84	1259.97	1264.09	1268.20	1272.30	1276.40
	1277.74	1281.25	1284.76	1288.27	1291.77	1295.27
	1477.46	1482.46	1487.46	1492.45	1497.43	1502.40
	1505.63	1510.91	1516.18	1521.43	1526.68	1531.91
	2974.15	2981.86	2989.54	2997.17	3004.77	3012.32
	3004.90	3012.70	3020.46	3028.19	3035.87	3043.52
	3037.62	3045.53	3053.40	3061.21	3068.98	3076.70
	3055.99	3063.94	3071.84	3079.70	3087.51	3095.27
	3280.33	3291.36	3302.30	3313.16	3323.95	3334.65
CH_2CCHCl	357.91	359.29	360.65	362.00	363.34	364.67
	502.23	503.84	505.43	507.01	508.58	510.13
	607.82	609.49	611.16	612.81	614.47	616.11
	742.21	744.80	747.37	749.92	752.44	754.94
	866.86	870.24	873.60	876.94	880.27	883.58
	939.19	943.36	947.52	951.66	955.81	959.94
	1019.47	1023.01	1026.54	1030.06	1033.57	1037.07
	1124.05	1127.07	1130.08	1133.07	1136.04	1139.00
	1260.74	1265.07	1269.37	1273.66	1277.94	1282.21
	1450.60	1455.55	1460.48	1465.40	1470.32	1475.23
	2014.23	2019.47	2024.68	2029.87	2035.03	2040.16
	2985.91	2994.00	3002.04	3010.05	3018.02	3025.95
	2989.11	2997.72	3006.27	3014.77	3023.22	3031.62
	3015.20	3023.54	3031.84	3040.11	3048.35	3056.55
CH_2CHCHO	344.70	345.54	346.38	347.22	348.06	348.89
	573.66	574.96	576.24	577.52	578.80	580.06
	631.30	633.12	634.94	636.75	638.55	640.34
	928.48	930.94	933.39	935.83	938.27	940.69
	1008.47	1012.59	1016.69	1020.78	1024.87	1028.94
	1026.18	1030.19	1034.12	1037.97	1041.73	1045.39
	1033.96	1037.08	1040.12	1043.07	1045.94	1048.73
	1158.87	1162.27	1165.66	1169.05	1172.42	1175.79
	1289.28	1293.46	1297.63	1301.78	1305.93	1310.05
	1371.98	1376.78	1381.55	1386.30	1391.02	1395.72
	1442.92	1447.81	1452.69	1457.55	1462.39	1467.21
	1651.30	1656.56	1661.79	1666.99	1672.15	1677.28
	1747.68	1754.25	1760.77	1767.23	1773.64	1780.00
	2694.74	2705.61	2716.40	2727.10	2737.71	2748.24
	3000.26	3008.32	3016.34	3024.30	3032.22	3040.09
	2994.05	3002.33	3010.56	3018.75	3026.89	3035.00
	3040.77	3048.62	3056.44	3064.22	3071.96	3079.67
cyclo-C_3H_6	776.89	779.09	781.27	783.44	785.60	787.74
	772.04	774.33	776.60	778.87	781.11	783.35
	882.95	885.22	887.47	889.72	891.95	894.18
	859.13	861.14	863.13	865.11	867.08	869.03
	859.12	861.13	863.11	865.08	867.02	868.95
	1062.50	1066.54	1070.58	1074.61	1078.64	1082.67
	1057.10	1061.23	1065.35	1069.46	1073.58	1077.69
	1095.18	1099.51	1103.83	1108.14	1112.44	1116.72
	1142.99	1146.82	1150.64	1154.45	1158.26	1162.06
	1204.36	1202.08	1204.63	1207.16	1209.68	1212.19
	1204.17	1208.25	1212.13	1216.01	1219.88	1223.74
	1199.52	1208.08	1211.97	1215.86	1219.73	1223.60
	1457.54	1462.42	1467.28	1472.14	1477.00	1481.85
	1456.08	1461.00	1465.90	1470.80	1475.70	1480.58

	1503.33	1508.55	1513.76	1518.95	1524.13	1529.30
	2998.37	3005.60	3012.80	3019.96	3027.10	3034.20
	3006.91	3014.10	3021.25	3028.37	3035.45	3042.50
	3038.31	3045.64	3052.94	3060.20	3067.43	3074.64
	3058.03	3065.39	3072.71	3079.99	3087.23	3094.43
	3058.49	3065.84	3073.14	3080.41	3087.63	3094.81
	3100.88	3108.23	3115.54	3122.81	3130.05	3137.25
$CH_2CHCHCH_2$	526.14	531.51	532.64	533.75	534.86	535.96
	593.51	595.65	597.06	598.47	599.86	601.23
	801.62	803.81	806.15	808.48	810.80	813.11
	902.80	910.73	912.62	914.50	916.37	918.24
	965.78	970.30	974.35	978.37	982.38	986.37
	968.55	973.15	977.29	981.41	985.50	989.58
	1007.42	1011.35	1015.29	1019.22	1023.14	1027.03
	1013.29	1024.97	1028.13	1031.27	1034.40	1037.51
	1058.38	1062.02	1065.55	1069.07	1072.58	1076.07
	1213.97	1219.29	1222.55	1225.80	1229.06	1232.31
	1304.54	1310.93	1315.08	1319.21	1323.32	1327.42
	1309.04	1315.99	1320.31	1324.62	1328.91	1333.19
	1401.18	1410.09	1414.55	1419.00	1423.42	1427.83
	1457.07	1467.05	1471.89	1476.72	1481.53	1486.34
	1624.95	1630.77	1635.80	1640.81	1645.78	1650.73
	1677.17	1682.62	1688.24	1693.84	1699.41	1704.95
	3002.46	3009.60	3018.27	3026.92	3035.55	3044.16
	3019.22	3022.43	3029.08	3035.89	3042.82	3049.84
	3040.41	3045.89	3054.25	3062.49	3070.58	3078.51
	2985.19	2989.10	2994.91	3001.16	3007.75	3014.59
	3085.62	3089.83	3097.45	3105.03	3112.58	3120.09
	3081.98	3086.40	3094.16	3101.89	3109.57	3117.22
$^1CH_2$	1348.96	1353.29	1357.62	1361.97	1366.32	1370.68
	2817.69	2827.56	2837.34	2847.06	2856.71	2866.30
	2819.87	2829.49	2839.03	2848.50	2857.90	2867.22
$^3CH_2$	947.17	953.68	960.14	966.55	972.93	979.26
	3041.46	3048.84	3056.19	3063.51	3070.81	3078.08
	3226.45	3233.51	3240.55	3247.55	3254.53	3261.48
HCO	1070.70	1074.30	1077.90	1081.50	1085.09	1088.69
	1915.64	1921.97	1928.23	1934.43	1940.56	1946.61
	2397.64	2408.09	2418.46	2428.75	2438.95	2449.05

Table S8. TOSH 2MR vibrational frequencies in cm^{-1} for the modified F1 anharmonic test set calculated using the 6-311++G(d,p) basis set and modified KS-B3LYP functional with different amounts of exact Hartree-Fock exchange parameterized between 34-40%.

Molecule	34% HF	36% HF	38% HF	40% HF
$NClF_2$	367.17	369.34	371.46	373.54
	380.49	383.66	386.77	389.81
	576.50	579.51	582.45	585.31
	706.59	711.24	715.86	720.44
	877.67	886.85	895.91	904.86
	973.23	979.48	985.70	991.89
ClF_3	399.06	397.60	400.97	404.26
	497.08	499.34	502.34	505.34
	667.70	667.74	668.88	669.99
	711.57	716.30	721.72	727.05
HOCl	717.83	721.21	724.57	727.91
	1233.29	1237.85	1242.38	1246.88
	3644.22	3657.33	3670.37	3683.30
ClNO	324.27	324.40	324.47	324.50
	607.37	609.09	610.81	612.52
	1946.36	1952.66	1958.78	1964.71
$ClNO_2$	380.54	383.87	387.31	390.83
	424.01	426.47	428.90	431.31
	682.75	685.97	689.17	692.33
	824.43	827.73	831.04	834.37
	1362.04	1366.01	1369.96	1373.90
	1741.95	1744.06	1746.06	1747.97
ClSN	394.54	396.16	397.79	399.45
	1375.15	1378.44	1381.49	1384.28
NCl_2F	350.98	353.66	356.28	358.83
	437.04	439.66	442.19	444.64
	624.14	626.83	629.51	632.17
	691.77	699.09	706.35	713.53
	867.27	875.14	882.93	890.66
Cl_2O	646.98	649.69	652.40	652.48
	667.95	675.86	683.62	687.52
$SOCl_2$	329.24	331.36	333.47	335.56
	427.63	430.29	432.97	435.65
	470.61	473.04	475.46	477.87
	1240.49	1244.37	1248.12	1251.73
SCl_2	482.11	484.73	487.32	489.91
	496.77	498.66	500.54	502.41
HOF	965.04	970.47	975.85	981.19
	1403.71	1410.09	1416.42	1422.69
	3603.72	3617.20	3630.59	3643.90
ONF	511.54	514.14	516.80	519.52
	779.66	783.05	786.49	789.97
	1966.69	1971.82	1976.82	1981.71
NSF	356.90	359.55	362.19	364.84
	589.68	592.66	595.67	598.71
	1412.40	1415.67	1418.74	1421.62
F_2NH	511.88	514.71	517.48	520.21
	903.39	911.22	918.93	926.53

	1014.00	1019.45	1024.86	1030.20
	1321.13	1325.29	1329.43	1333.54
	1465.28	1472.22	1479.09	1485.89
	3220.00	3234.03	3247.91	3261.66
N_2F_2	373.22	375.14	377.04	378.92
	432.93	434.96	436.95	438.92
	620.03	623.17	626.26	629.31
	1007.47	1013.37	1019.19	1024.95
	1069.79	1076.45	1083.02	1089.51
	1654.98	1664.98	1674.88	1684.69
F_2O	492.89	496.18	499.41	502.60
	903.16	912.68	922.09	931.39
	1017.77	1023.10	1028.40	1033.66
F_2SO	340.09	343.00	345.86	348.68
	367.61	369.88	372.13	374.37
	496.42	499.87	503.29	506.66
	672.77	676.99	681.19	685.38
	746.36	750.60	754.81	758.98
	1322.35	1327.01	1331.56	1336.02
S_2F_2	555.46	554.40	553.44	552.57
	639.23	644.61	649.94	655.20
	675.42	680.64	685.80	690.88
HNO_3	376.82	378.67	380.39	381.98
	603.96	607.13	610.23	613.29
	672.85	677.49	682.00	686.37
	788.70	792.20	795.66	799.10
	926.13	932.43	938.69	944.91
	1329.50	1335.12	1340.64	1346.06
	1371.40	1377.33	1383.25	1389.17
	1756.61	1761.75	1766.79	1771.76
	3529.47	3542.22	3554.89	3567.47
HN_3	532.93	533.73	534.51	535.27
	595.31	596.96	598.59	600.20
	1177.11	1180.10	1182.84	1185.30
	1306.40	1310.48	1314.59	1318.75
	2260.93	2267.35	2273.72	2280.02
	3343.89	3353.10	3362.27	3371.43
H_2O	1576.93	1581.72	1586.52	1591.32
	3780.93	3792.75	3804.50	3816.18
	3798.57	3810.16	3821.67	3833.11
H_2O_2	210.51	210.66	210.79	210.88
	951.78	957.58	963.34	969.04
	1297.26	1302.49	1307.65	1312.76
	1465.95	1471.65	1477.29	1482.89
	3606.38	3619.54	3632.62	3645.61
	3681.58	3694.69	3707.70	3720.64
H_2S	1207.90	1212.32	1216.75	1221.19
	2664.90	2672.75	2680.57	2688.36
	2634.76	2642.41	2650.02	2657.59
H_2S_2	394.59	394.40	394.15	393.95
	481.91	483.83	485.75	487.66
	897.00	900.47	903.89	907.27
	901.45	904.71	907.92	911.10
	2596.04	2605.03	2613.97	2622.85

	2556.76	2565.80	2574.77	2583.68
NO_2	772.25	774.49	776.70	778.88
	1421.05	1426.93	1432.73	1438.43
	1680.62	1684.73	1688.62	1692.29
N_2O	597.99	599.99	601.99	603.96
	597.99	599.99	601.99	603.96
	1321.11	1322.72	1324.14	1325.39
	2339.84	2347.13	2354.38	2361.60
SO_2	520.66	523.46	526.23	528.98
	1169.44	1175.85	1182.20	1188.47
	1343.49	1350.56	1357.54	1364.44
O_3	761.42	764.70	767.90	771.04
	1239.65	1249.42	1258.62	1267.28
	1292.67	1300.25	1307.72	1315.10
COClF	413.98	415.15	416.31	417.46
	501.76	503.58	505.39	507.18
	677.67	680.18	682.68	685.16
	771.03	773.83	776.60	779.35
	1078.61	1083.76	1088.86	1093.90
	1930.45	1936.01	1941.52	1946.99
ClCN	396.56	398.01	399.46	400.91
	396.56	398.01	399.46	400.91
	742.51	744.11	745.69	747.25
	2330.99	2339.33	2347.61	2355.83
$COCl_2$	449.72	451.49	453.25	454.99
	565.08	566.86	568.63	570.40
	592.38	594.33	596.26	598.18
	834.39	838.31	842.21	846.09
	1884.43	1889.72	1894.97	1900.18
FCN	494.05	496.16	498.26	500.38
	494.05	496.16	498.26	500.38
	1094.24	1097.43	1100.59	1103.71
	2428.64	2436.87	2445.04	2453.15
COF_2	583.75	585.64	427.93	589.38
	626.91	629.19	535.60	633.70
	786.90	790.15	634.52	796.58
	974.56	978.24	808.11	985.46
	1217.84	1223.65	1191.46	1235.05
	1984.25	1989.95	1373.54	2001.18
CSF_2	425.42	426.68	427.93	429.16
	532.47	534.05	535.60	537.14
	629.80	632.17	634.52	636.86
	803.07	805.61	808.11	810.58
	1180.03	1185.79	1191.46	1197.06
	1365.46	1369.54	1373.54	1377.47
COS	521.80	523.64	525.46	527.21
	521.80	523.64	525.46	527.21
	875.93	877.03	878.08	879.08
	2116.28	2121.49	2126.63	2131.72
CO_2	684.70	687.59	690.47	693.33
	684.70	687.59	690.47	693.33
	1393.54	1398.00	1402.42	1406.78
	2406.06	2410.84	2415.54	2420.17
CS_2	405.06	406.32	407.58	408.82

	405.06	406.32	407.58	408.82
	682.27	683.95	685.61	687.25
	1543.90	1545.52	1547.06	1548.51
HCN	758.28	762.12	766.00	769.77
	758.28	762.12	766.00	769.77
	2216.68	2223.68	2230.64	2237.54
	3322.50	3330.72	3338.89	3347.02
HNCO	600.28	600.93	601.58	602.24
	642.71	645.28	647.84	650.38
	790.92	793.75	796.61	799.50
	1351.89	1355.54	1359.14	1362.69
	2322.58	2326.64	2330.64	2334.57
	3537.34	3545.96	3554.52	3563.02
H_2CO	1200.76	1205.46	1210.16	1214.86
	1267.82	1271.78	1275.73	1279.66
	1536.29	1541.08	1545.85	1550.61
	1828.28	1833.71	1839.11	1844.48
	2836.57	2846.21	2855.79	2865.32
	2822.78	2833.27	2843.67	2853.98
HCOOH	439.11	436.73	434.40	432.11
	677.27	679.39	681.49	683.57
	1042.22	1046.88	1051.52	1056.14
	1104.09	1107.66	1111.17	1114.64
	1264.93	1269.14	1273.33	1277.49
	1428.04	1433.33	1438.58	1443.80
	1868.66	1874.08	1879.47	1884.81
	2840.67	2850.48	2860.24	2869.95
	3608.60	3621.88	3635.02	3648.03
C_2Cl_2	411.18	410.91	411.00	411.41
	411.18	410.91	411.00	411.41
	476.81	477.63	478.44	479.25
	986.41	988.10	989.77	991.45
	2328.01	2335.26	2342.46	2349.60
C_2N_2	565.70	567.38	569.07	570.77
	565.70	567.38	569.07	570.77
	886.11	887.34	888.57	889.79
	2293.03	2301.26	2309.43	2317.52
	2474.59	2483.66	2492.66	2501.59
HCCCl	375.92	376.63	377.44	378.27
	375.92	376.63	377.44	378.27
	689.70	693.37	698.03	702.21
	689.70	693.37	698.03	702.21
	748.90	750.16	751.42	752.67
	2197.61	2203.85	2210.06	2216.23
	3326.02	3333.72	3341.43	3349.07
HCCF	421.82	424.75	427.65	430.52
	421.82	424.75	427.65	430.52
	610.49	615.35	620.18	625.00
	610.49	615.35	620.18	625.00
	1077.23	1079.69	1082.12	1084.55
	2318.40	2324.67	2330.90	2337.10
	3338.72	3346.38	3354.01	3361.60
HCCH	767.85	769.19	770.64	772.21
	767.85	769.19	770.64	772.21

	820.35	823.20	826.05	828.91
	820.35	823.20	826.05	828.91
	2065.94	2071.31	2076.65	2081.96
	3277.37	3284.87	3292.34	3299.78
	3428.50	3436.19	3443.85	3451.47
trans-CHClCHCl	352.67	353.52	354.35	355.18
	826.55	805.48	807.42	809.35
	803.53	830.28	833.98	837.65
	851.68	854.05	856.40	858.73
	955.32	959.38	963.42	967.45
	1241.63	1245.92	1250.17	1254.40
	1320.12	1324.57	1328.99	1333.39
	1651.67	1656.97	1662.23	1667.46
	3094.01	3102.12	3110.20	3118.24
	3147.83	3155.94	3164.02	3172.07
cis-CHClCHCl	425.65	426.84	428.03	429.21
	572.94	574.25	575.56	576.85
	706.58	708.13	709.67	711.20
	754.57	757.52	760.46	763.39
	848.23	850.52	852.79	855.05
	950.26	954.91	959.52	964.10
	1236.55	1240.68	1244.81	1248.92
	1332.04	1336.73	1341.41	1346.07
	1652.41	1657.70	1662.95	1668.18
	3066.45	3074.44	3082.40	3090.33
	3139.89	3147.89	3155.85	3163.79
CH_2CCl_2	304.31	304.95	305.59	306.22
	397.22	398.15	399.08	400.00
	486.17	487.31	488.44	489.56
	600.19	601.65	603.10	604.53
	732.94	735.50	738.06	740.59
	779.39	782.46	785.51	788.53
	964.96	969.57	974.16	978.73
	1115.23	1119.22	1123.18	1127.12
	1418.61	1423.42	1428.22	1433.00
	1666.49	1671.78	1677.04	1682.29
	3084.77	3092.21	3099.63	3107.02
	3113.61	3121.17	3128.69	3136.18
cis-CHFCHF	518.30	519.92	521.52	523.11
	778.01	780.09	782.15	784.20
	819.89	823.28	826.66	830.02
	919.77	924.43	929.06	933.64
	1023.31	1026.06	1028.78	1031.48
	1126.40	1129.39	1132.35	1135.28
	1296.69	1300.82	1304.94	1309.03
	1397.44	1402.23	1406.99	1411.73
	1762.77	1768.59	1774.37	1780.10
	3090.24	3098.75	3107.21	3115.62
	3164.87	3173.37	3181.82	3190.22
trans-CHFCHF	329.28	330.36	331.42	332.47
	338.25	339.43	340.60	341.76
	558.30	559.81	561.31	562.79
	857.34	861.54	865.71	869.84
	931.62	935.80	939.97	944.12

	1139.79	1142.96	1146.10	1149.21
	1147.28	1150.20	1153.10	1155.97
	1304.69	1309.20	1313.70	1318.17
	1320.81	1325.17	1329.52	1333.85
	1750.99	1756.81	1762.60	1768.34
	3099.50	3107.97	3116.39	3124.78
	3156.58	3165.04	3173.46	3181.83
OCHCHO	354.78	356.13	357.47	358.81
	557.40	559.25	561.07	562.89
	824.01	827.57	831.09	834.59
	1057.89	1061.73	1065.55	1069.35
	1094.07	1098.30	1102.51	1106.70
	1349.24	1354.00	1358.74	1363.46
	1389.88	1394.60	1399.31	1404.01
	1813.85	1819.55	1825.19	1830.78
	1829.36	1836.11	1842.80	1849.44
	2821.04	2831.36	2841.60	2851.77
	2877.72	2887.78	2897.76	2907.68
CH_2CH_2	861.12	863.42	865.71	868.00
	996.50	1000.25	1003.98	1007.72
	999.54	1003.62	1007.68	1011.72
	1065.66	1068.64	1071.60	1074.56
	1253.99	1257.89	1261.79	1265.68
	1388.78	1392.53	1396.27	1399.99
	1482.52	1487.29	1492.06	1496.81
	1689.30	1694.11	1698.91	1703.70
	3053.08	3060.49	3067.88	3075.23
	3092.86	3100.41	3107.92	3115.40
	3110.09	3117.64	3125.15	3132.62
	3140.27	3147.78	3155.26	3162.70
cyclo-C_2H_4O	843.36	845.80	848.23	850.64
	839.61	842.73	845.80	848.82
	887.24	889.73	892.20	894.66
	1061.27	1065.01	1068.74	1072.45
	1173.41	1178.12	1182.81	1187.49
	1185.22	1189.91	1194.58	1199.24
	1184.45	1188.39	1192.31	1196.21
	1192.08	1195.93	1199.77	1203.59
	1309.53	1313.32	1317.10	1320.86
	1513.29	1518.32	1523.35	1528.36
	1549.52	1554.85	1560.16	1565.46
	3012.92	3020.87	3028.78	3036.65
	3044.00	3052.04	3060.04	3068.00
	3079.91	3088.06	3096.15	3104.19
	3097.71	3105.88	3114.00	3122.06
cyclo-C_2H_4NH	812.34	814.87	817.38	819.86
	852.06	854.88	857.66	860.41
	877.29	879.67	882.04	884.40
	951.53	954.49	957.44	960.38
	1031.52	1034.32	1037.12	1039.90
	1136.46	1140.66	1144.86	1149.05
	1144.04	1148.52	1152.99	1157.44
	1165.20	1169.13	1173.06	1176.98
	1252.22	1256.14	1260.03	1263.89

	1280.50	1284.58	1288.66	1292.74
	1298.76	1302.25	1305.75	1309.24
	1507.37	1512.33	1517.29	1522.24
	1537.13	1542.35	1547.55	1552.74
	3019.84	3027.32	3034.76	3042.17
	3051.13	3058.70	3066.24	3073.74
	3084.37	3092.00	3099.58	3107.11
	3102.99	3110.66	3118.29	3125.88
	3345.28	3355.83	3366.31	3376.71
CH_2CCHCl	365.99	367.31	368.61	369.90
	511.67	513.20	514.72	516.22
	617.75	619.38	621.01	622.63
	757.42	759.88	762.31	764.72
	886.87	890.15	893.42	896.66
	964.06	968.18	972.28	976.38
	1040.56	1044.04	1047.52	1050.98
	1141.94	1144.86	1147.77	1150.67
	1286.45	1290.69	1294.91	1299.12
	1480.13	1485.03	1489.91	1494.79
	2045.27	2050.36	2055.42	2060.46
	3033.85	3041.71	3049.53	3057.32
	3039.96	3048.26	3056.51	3064.71
	3064.72	3072.85	3080.95	3089.02
CH_2CHCHO	349.73	350.56	351.38	352.21
	581.32	582.57	583.81	585.04
	642.12	643.89	645.65	647.40
	943.10	945.50	947.90	950.28
	1032.99	1037.04	1041.08	1045.10
	1048.95	1052.42	1055.81	1059.14
	1051.45	1054.12	1056.79	1059.46
	1179.15	1182.49	1185.83	1189.16
	1314.17	1318.27	1322.36	1326.44
	1400.40	1405.05	1409.69	1414.30
	1472.02	1476.81	1481.58	1486.34
	1682.39	1687.46	1692.50	1697.52
	1786.30	1792.55	1798.76	1804.90
	2758.68	2769.04	2779.32	2789.53
	3047.91	3055.70	3063.44	3071.13
	3043.06	3051.08	3059.06	3067.00
	3087.35	3094.99	3102.59	3110.17
cyclo-C_3H_6	789.87	791.99	794.10	796.19
	785.57	787.77	789.97	792.15
	896.40	898.61	900.81	903.01
	870.97	872.89	874.81	876.71
	870.87	872.76	874.64	876.50
	1086.70	1090.72	1094.74	1098.75
	1081.79	1085.90	1089.99	1094.09
	1121.00	1125.27	1129.52	1133.77
	1165.86	1169.66	1173.45	1177.24
	1214.68	1217.16	1219.62	1222.08
	1227.59	1231.43	1235.27	1239.10
	1227.46	1231.31	1235.16	1238.99
	1486.69	1491.53	1496.36	1501.19
	1485.46	1490.34	1495.20	1500.06

	1534.46	1539.61	1544.75	1549.87
	3041.28	3048.32	3055.34	3062.33
	3049.51	3056.49	3063.43	3070.34
	3081.80	3088.94	3096.05	3103.13
	3101.58	3108.70	3115.78	3122.82
	3101.95	3109.05	3116.12	3123.14
	3144.41	3151.54	3158.63	3165.69
$CH_2CHCHCH_2$	537.06	538.14	539.22	540.30
	602.60	603.95	605.29	606.62
	815.40	817.68	819.94	822.20
	920.09	921.94	923.78	925.61
	990.33	994.28	998.21	1002.13
	993.63	997.67	1001.68	1005.68
	1030.91	1034.77	1046.75	1049.80
	1040.60	1043.68	1038.62	1042.45
	1079.56	1083.03	1086.49	1089.94
	1235.55	1238.79	1242.03	1245.26
	1331.50	1335.57	1339.62	1343.66
	1337.45	1341.69	1345.92	1350.13
	1432.22	1436.59	1440.95	1445.29
	1491.13	1495.91	1500.68	1505.44
	1655.66	1660.56	1665.44	1670.29
	1710.47	1715.95	1721.41	1726.84
	3052.75	3061.29	3069.79	3078.24
	3056.93	3064.05	3071.21	3078.38
	3086.28	3093.88	3101.32	3108.61
	3021.61	3028.75	3035.98	3043.27
	3127.55	3134.98	3142.37	3149.72
	3124.82	3132.38	3139.91	3147.38
$^1CH_2$	1375.04	1379.41	1383.79	1388.17
	2875.81	2885.26	2894.65	2903.97
	2876.48	2885.67	2894.79	2903.84
$^3CH_2$	985.55	991.81	998.02	1004.20
	3085.32	3092.54	3099.73	3106.90
	3268.41	3275.30	3282.17	3289.01
HCO	1092.29	1095.90	1099.50	1103.11
	1952.61	1958.54	1964.40	1970.20
	2459.10	2469.07	2478.96	2488.77

Table S9. TOSH 2MR vibrational frequencies in cm^{-1} for the modified F1 anharmonic test set calculated using the 6-311++G(d,p) basis set and modified TAO-B3LYP functional with different amounts of exact Hartree-Fock exchange parameterized between 10-20%.

Molecule	10% HF	12% HF	14% HF	16% HF	18% HF	20% HF
$NClF_2$	323.02	331.06	338.01	343.95	347.25	350.32
	332.07	336.44	340.37	344.12	349.60	354.59
	530.13	535.31	540.18	544.76	549.09	553.20
	622.96	634.33	644.27	653.09	661.03	668.27
	762.19	772.84	783.35	793.70	803.92	813.99
	898.20	904.94	911.61	918.20	924.75	931.24
ClF_3	320.90	333.55	344.09	353.00	360.66	367.35
	451.12	458.08	463.72	468.58	472.90	476.86
	548.66	573.92	596.13	615.36	631.91	646.21
	619.12	629.15	637.40	644.15	649.68	654.22
HOCl	633.09	647.42	659.29	669.22	677.65	684.91
	1157.29	1167.28	1176.03	1183.83	1190.90	1197.41
	3488.18	3503.54	3518.51	3533.13	3547.46	3561.53
ClNO	256.14	267.45	277.73	286.70	294.31	295.04
	505.87	521.20	534.92	547.04	557.64	562.36
	1867.43	1874.31	1881.34	1888.44	1895.55	1902.17
$ClNO_2$	297.78	310.92	321.94	331.27	339.27	346.23
	374.98	382.14	388.25	393.54	398.21	402.39
	593.63	607.81	619.68	629.75	638.39	645.90
	763.24	771.24	778.36	784.74	790.50	795.76
	1304.66	1310.97	1316.92	1322.54	1327.87	1332.96
	1705.31	1710.00	1714.39	1718.50	1722.33	1725.91
ClSN	318.27	328.23	337.33	345.58	352.99	359.62
	1291.81	1302.19	1311.93	1320.99	1329.37	1337.09
NCl_2F	296.41	304.38	311.17	317.07	322.29	326.98
	381.55	389.04	395.71	401.69	407.07	411.96
	562.12	571.50	579.56	586.55	592.65	598.04
	566.31	581.39	595.15	607.77	619.45	630.33
	770.98	779.45	787.90	796.29	804.66	812.97
Cl_2O	396.61	441.11	478.79	510.67	537.74	560.90
	577.33	588.01	597.33	605.41	612.41	618.53
$SOCl_2$	287.21	293.04	298.20	300.39	304.68	308.58
	365.84	374.50	382.12	387.36	393.42	398.87
	413.44	421.57	428.70	433.98	439.67	444.76
	1178.54	1185.72	1192.45	1197.84	1203.86	1209.55
SCl_2	369.78	385.54	399.65	412.15	423.16	432.85
	439.93	448.24	455.57	462.03	467.71	472.73
HOF	860.63	878.27	892.47	904.22	914.21	922.93
	1312.01	1323.34	1333.33	1342.38	1350.73	1358.59
	3451.43	3465.71	3479.88	3493.93	3507.86	3521.68
ONF	465.69	472.65	478.63	483.83	488.41	492.49
	719.01	728.26	736.13	742.90	748.81	754.05
	1887.64	1896.15	1904.25	1911.95	1919.29	1926.29
NSF	321.46	325.16	328.67	332.01	335.22	338.32
	547.69	552.91	557.65	561.99	566.00	569.76
	1336.55	1346.66	1355.87	1364.26	1371.90	1378.89
F_2NH	472.92	477.16	481.13	484.88	488.46	491.89
	796.50	808.17	819.04	829.28	839.04	848.40

	945.65	952.54	959.15	965.52	971.72	977.76
	1268.85	1274.31	1279.45	1284.36	1289.09	1293.68
	1378.20	1386.58	1394.72	1402.65	1410.42	1418.04
	3057.77	3073.24	3088.54	3103.67	3118.62	3133.39
N_2F_2	344.08	347.50	350.61	353.48	356.15	358.67
	405.48	408.31	411.01	413.60	416.09	418.50
	575.45	580.66	585.41	589.80	593.92	597.83
	933.72	941.26	948.46	955.38	962.09	968.63
	981.26	990.52	999.29	1007.65	1015.69	1023.47
	1520.71	1534.19	1547.09	1559.49	1571.48	1583.12
F_2O	428.71	439.23	447.74	454.82	460.87	466.19
	704.43	738.07	765.15	787.46	806.32	822.68
	928.66	941.87	952.97	962.52	970.91	978.47
F_2SO	302.61	306.21	309.72	313.15	316.51	319.79
	338.75	341.53	344.22	346.84	349.40	351.91
	452.54	456.80	460.93	464.96	468.89	472.75
	623.09	627.99	632.73	637.34	641.84	646.27
	695.65	700.72	705.62	710.38	715.02	719.56
	1260.04	1266.62	1272.93	1279.00	1284.86	1290.53
S_2F_2	559.13	567.74	571.73	570.84	569.79	568.62
	572.74	572.39	575.82	583.47	590.76	597.73
	601.74	609.53	616.96	624.04	630.82	637.34
HNO_3	340.20	345.00	349.48	353.63	357.45	360.95
	560.24	564.78	569.13	573.30	577.30	581.15
	608.43	615.10	621.51	627.70	633.68	639.47
	732.62	739.32	745.40	750.96	756.12	760.95
	849.40	856.80	863.96	870.93	877.75	884.45
	1254.13	1261.64	1268.94	1276.02	1282.90	1289.59
	1294.21	1302.19	1309.78	1317.03	1324.00	1330.73
	1683.63	1692.08	1699.94	1707.32	1714.29	1720.92
	3381.65	3395.57	3409.32	3422.90	3436.33	3449.61
HN_3	513.87	516.68	519.15	521.33	523.28	525.03
	571.82	574.46	576.94	579.30	581.54	583.68
	1128.18	1133.37	1138.44	1143.40	1148.22	1152.91
	1261.15	1265.45	1269.62	1273.70	1277.70	1281.66
	2178.62	2187.18	2195.40	2203.33	2211.00	2218.45
	3238.31	3249.28	3259.90	3270.24	3280.31	3290.17
H_2O	1524.13	1528.82	1533.50	1538.17	1542.85	1547.52
	3648.96	3661.71	3674.28	3686.69	3698.96	3711.09
	3669.29	3681.78	3694.09	3706.24	3718.26	3730.15
H_2O_2	177.15	185.60	191.80	196.40	199.85	202.46
	862.68	874.92	885.43	894.71	903.09	910.79
	1223.05	1231.86	1239.80	1247.10	1253.91	1260.36
	1393.98	1401.21	1408.14	1414.83	1421.31	1427.64
	3455.11	3469.51	3483.73	3497.78	3511.66	3525.38
	3530.88	3545.27	3559.45	3573.45	3587.28	3600.95
H_2S	1157.70	1162.41	1167.04	1171.60	1176.12	1180.62
	2576.15	2585.58	2594.69	2603.52	2612.13	2620.55
	2543.86	2553.91	2563.49	2572.68	2581.55	2590.15
H_2S_2	372.25	376.50	379.96	382.83	385.14	387.02
	433.60	440.83	447.09	452.56	457.36	461.64
	839.57	846.49	852.83	858.69	864.12	869.20
	848.72	854.95	860.70	866.05	871.04	875.73
	2496.19	2505.81	2515.36	2524.82	2534.21	2543.49

	2456.35	2466.05	2475.68	2485.21	2494.65	2503.99
NO_2	709.96	719.73	728.02	735.09	741.18	746.47
	1325.65	1336.87	1347.21	1356.79	1365.73	1374.12
	1598.55	1609.87	1620.03	1629.21	1637.56	1645.19
N_2O	571.36	574.25	576.93	579.56	582.06	584.46
	571.36	574.25	576.93	579.56	582.06	584.46
	1290.79	1294.82	1298.60	1302.14	1305.46	1308.56
	2254.75	2262.99	2271.05	2278.97	2286.76	2294.43
SO_2	477.77	482.58	487.04	491.22	495.15	498.87
	1069.57	1080.73	1091.15	1100.92	1110.14	1118.88
	1238.36	1250.11	1261.07	1271.37	1281.10	1290.34
O_3	652.66	669.12	683.79	696.64	707.77	717.39
	1005.11	1036.23	1064.69	1090.52	1113.86	1134.96
	1114.44	1136.80	1157.74	1176.96	1194.39	1210.14
COClF	399.80	401.22	402.59	403.92	405.22	406.49
	480.27	482.46	484.58	486.64	488.66	490.63
	642.12	645.98	649.59	652.98	656.21	659.29
	737.28	740.68	743.97	747.17	750.30	753.35
	1016.58	1022.50	1028.33	1034.06	1039.70	1045.27
	1860.41	1867.46	1874.24	1880.80	1887.18	1893.40
ClCN	377.60	379.60	381.49	383.29	385.02	386.68
	377.60	379.60	381.49	383.29	385.02	386.68
	723.23	725.55	727.74	729.81	731.78	733.66
	2230.99	2240.42	2249.69	2258.80	2267.79	2276.66
$COCl_2$	427.55	429.81	431.91	434.03	436.08	438.09
	544.43	547.07	548.03	550.36	552.57	554.68
	559.20	563.29	566.59	569.98	573.11	576.02
	788.76	793.91	796.23	800.98	805.56	810.00
	1812.59	1819.98	1827.34	1834.07	1840.55	1846.81
FCN	468.39	470.85	473.22	475.52	477.77	479.97
	468.39	470.85	473.22	475.52	477.77	479.97
	1055.46	1059.32	1063.08	1066.75	1070.33	1073.84
	2331.89	2341.00	2349.99	2358.85	2367.61	2376.26
COF_2	560.89	563.01	565.10	567.17	569.21	571.22
	598.71	601.34	603.93	606.48	608.99	611.46
	745.72	749.72	753.57	757.31	760.96	764.53
	930.50	934.70	938.83	942.91	946.92	950.88
	1147.81	1154.45	1161.00	1167.45	1173.81	1180.09
	1915.70	1922.50	1929.10	1935.55	1941.86	1948.04
CSF_2	406.18	408.26	410.23	412.09	413.86	415.55
	510.33	512.61	514.78	516.87	518.88	520.83
	577.73	584.10	589.86	595.10	599.88	604.28
	766.25	770.16	773.89	777.45	780.87	784.15
	1112.19	1118.21	1124.25	1130.28	1136.30	1142.29
	1305.41	1312.12	1318.49	1324.55	1330.31	1335.82
COS	494.21	497.19	499.91	502.56	505.08	507.49
	494.21	497.19	499.91	502.56	505.08	507.49
	859.70	861.96	864.06	866.03	867.88	869.62
	2053.18	2059.46	2065.58	2071.57	2077.42	2083.16
CO_2	649.73	652.94	656.10	659.20	662.27	665.31
	649.73	652.94	656.10	659.20	662.27	665.31
	1339.65	1344.73	1349.73	1354.66	1359.52	1364.32
	2351.06	2356.70	2362.23	2367.65	2372.97	2378.20
CS_2	378.91	381.83	384.52	387.01	389.35	391.54

	378.91	381.83	384.52	387.01	389.35	391.54
	657.94	660.59	663.12	665.54	667.86	670.09
	1511.30	1515.79	1519.94	1523.76	1527.28	1530.54
HCN	715.31	719.15	723.17	727.07	730.91	734.69
	715.31	719.15	723.17	727.07	730.91	734.69
	2134.58	2142.31	2149.94	2157.46	2164.89	2172.24
	3235.82	3244.34	3252.79	3261.15	3269.45	3277.67
HNCO	591.10	592.31	593.40	594.38	595.28	596.10
	611.57	614.50	617.37	620.19	622.96	625.70
	760.10	762.66	765.22	767.79	770.37	772.97
	1307.29	1311.69	1315.99	1320.19	1324.32	1328.36
	2275.48	2280.61	2285.57	2290.40	2295.11	2299.69
	3440.17	3450.14	3459.85	3469.34	3478.65	3487.78
H_2CO	1130.05	1138.33	1145.89	1152.84	1159.32	1165.41
	1216.16	1221.80	1227.10	1232.13	1236.93	1241.54
	1478.38	1484.25	1489.89	1495.34	1500.65	1505.82
	1751.09	1760.11	1768.40	1776.11	1783.33	1790.16
	2737.80	2747.14	2756.54	2765.98	2775.42	2784.86
	2713.12	2723.60	2734.14	2744.72	2755.29	2765.83
HCOOH	471.42	468.63	465.85	463.07	460.32	457.59
	650.72	653.30	655.80	658.25	660.64	662.99
	983.45	989.27	994.87	1000.29	1005.56	1010.70
	1061.11	1065.45	1069.68	1073.82	1077.87	1081.84
	1215.45	1220.06	1224.61	1229.10	1233.53	1237.92
	1366.56	1372.35	1378.06	1383.68	1389.25	1394.75
	1799.48	1806.66	1813.52	1820.12	1826.48	1832.66
	2739.35	2749.10	2758.84	2768.55	2778.22	2787.86
	3448.27	3464.06	3479.48	3494.56	3509.34	3523.84
C_2Cl_2	207.44	217.70	227.34	236.44	245.08	253.32
	207.44	217.70	227.34	236.44	245.08	253.32
	467.01	468.45	469.76	470.96	472.07	473.12
	965.94	969.06	971.87	974.44	976.80	979.00
	2269.36	2277.63	2285.67	2293.51	2301.18	2308.70
C_2N_2	545.84	547.64	549.38	551.08	552.76	554.43
	545.84	547.64	549.38	551.08	552.76	554.43
	875.17	876.25	877.34	878.45	879.55	880.67
	2189.95	2199.82	2209.45	2218.88	2228.11	2237.18
	2355.75	2367.45	2378.76	2389.74	2400.43	2410.85
HCCCl	369.22	369.38	369.63	369.94	370.32	370.78
	369.22	369.38	369.62	369.94	370.32	370.78
	644.27	648.33	652.35	656.34	660.29	664.22
	644.27	648.33	652.35	656.34	660.29	664.22
	734.16	736.04	737.78	739.41	740.95	742.42
	2122.87	2130.33	2137.59	2144.67	2151.60	2158.40
	3244.62	3252.72	3260.71	3268.61	3276.43	3284.16
HCCF	382.17	386.25	390.12	393.81	397.40	400.89
	382.17	386.25	390.12	393.81	397.40	400.89
	552.38	557.51	562.60	567.63	572.65	577.64
	552.38	557.50	562.60	567.63	572.65	577.64
	1048.69	1051.56	1054.35	1057.06	1059.71	1062.30
	2246.41	2253.48	2260.41	2267.22	2273.91	2280.51
	3258.70	3266.62	3274.45	3282.22	3289.90	3297.53
HCCH	747.61	748.12	748.76	749.39	750.32	751.39
	747.61	748.12	748.76	749.39	750.32	751.39

	787.65	790.60	793.51	796.35	799.22	802.08
	787.65	790.60	793.51	796.35	799.22	802.08
	2003.93	2010.07	2016.08	2021.97	2027.76	2033.47
	3200.03	3207.62	3215.15	3222.63	3230.06	3237.44
	3348.61	3356.49	3364.30	3372.04	3379.72	3387.35
trans-CHClCHCl	342.02	343.22	344.36	345.43	346.47	347.46
	776.02	781.05	785.90	790.57	795.10	799.49
	782.55	785.10	787.51	789.82	792.03	794.16
	824.18	827.09	829.89	832.60	835.24	837.80
	902.26	907.56	912.68	917.63	922.42	927.09
	1190.87	1195.50	1200.08	1204.60	1209.08	1213.53
	1266.71	1271.64	1276.51	1281.29	1286.02	1290.70
	1579.38	1586.91	1594.11	1601.02	1607.68	1614.11
	3009.22	3017.35	3025.44	3033.49	3041.49	3049.47
	3062.76	3070.92	3079.05	3087.13	3095.16	3103.16
cis-CHClCHCl	404.90	407.31	409.53	411.58	413.51	415.31
	557.35	559.02	560.61	562.15	563.63	565.08
	716.34	720.13	723.79	727.34	697.82	699.52
	690.21	692.27	694.21	696.06	730.79	734.15
	822.30	825.04	827.68	830.25	832.75	835.20
	890.66	896.49	902.14	907.64	913.00	918.25
	1185.88	1190.48	1195.01	1199.48	1203.90	1208.28
	1276.76	1281.81	1286.80	1291.74	1296.64	1301.50
	1581.65	1588.97	1595.99	1602.73	1609.25	1615.57
	2984.05	2992.07	3000.04	3007.96	3015.84	3023.69
	3056.76	3064.82	3072.84	3080.80	3088.73	3096.60
CH_2CCl_2	298.12	298.96	299.76	298.48	299.26	300.01
	384.88	386.08	387.23	388.44	389.53	390.60
	466.64	468.90	470.98	472.73	474.54	476.24
	583.91	585.88	587.75	588.75	590.47	592.13
	697.78	701.48	704.98	708.30	711.53	714.66
	744.14	748.00	751.72	753.87	757.37	760.78
	908.81	913.94	919.01	924.27	929.24	934.16
	1069.44	1073.70	1077.92	1081.63	1085.78	1089.90
	1362.40	1367.54	1372.63	1377.73	1382.73	1387.68
	1598.67	1605.58	1612.24	1618.61	1624.86	1630.95
	3008.00	3015.48	3022.92	3030.28	3037.62	3044.93
	3036.86	3044.44	3051.97	3059.40	3066.84	3074.24
cis-CHFCHF	494.53	497.12	499.54	501.82	503.98	506.04
	778.17	782.21	786.16	760.13	762.35	764.54
	753.27	755.60	757.88	790.00	793.77	797.47
	861.90	867.48	872.93	878.25	883.47	888.59
	991.21	994.40	997.52	1000.57	1003.57	1006.51
	1092.33	1095.63	1098.88	1102.08	1105.23	1108.34
	1248.49	1252.91	1257.28	1261.61	1265.90	1270.15
	1341.54	1346.66	1351.73	1356.75	1361.73	1366.68
	1691.94	1699.10	1706.05	1712.80	1719.40	1725.84
	3000.98	3009.69	3018.34	3026.93	3035.46	3043.92
	3075.24	3083.98	3092.65	3101.25	3109.79	3118.27
trans-CHFCHF	315.20	316.46	317.69	318.91	320.11	321.30
	321.94	323.70	325.35	326.92	328.42	329.85
	540.20	541.93	543.63	545.30	546.93	548.54
	803.81	809.06	814.15	819.10	823.93	828.65
	880.03	885.14	890.09	894.91	899.61	904.21

	1102.87	1106.46	1109.97	1113.43	1116.84	1120.20
	1114.40	1117.66	1120.86	1124.01	1127.11	1130.16
	1252.02	1256.79	1261.53	1266.23	1270.89	1275.53
	1269.24	1274.02	1278.73	1283.38	1287.97	1292.52
	1678.78	1686.12	1693.21	1700.10	1706.80	1713.35
	3011.37	3019.95	3028.47	3036.94	3045.36	3053.73
	3068.43	3077.03	3085.57	3094.05	3102.48	3110.86
OCHCHO	343.24	343.45	343.87	344.49	345.28	346.20
	536.54	538.31	540.15	542.03	543.94	545.86
	777.14	781.69	786.16	790.51	794.77	798.92
	1015.65	1025.52	1013.58	1019.36	1024.83	1030.02
	1001.02	1007.46	1034.51	1042.69	1050.17	1057.03
	1281.41	1288.58	1295.45	1301.99	1308.24	1314.23
	1322.18	1329.47	1336.39	1342.94	1349.19	1355.15
	1715.32	1729.47	1742.35	1754.11	1764.90	1768.76
	1720.84	1732.07	1742.37	1751.84	1760.60	1774.88
	2713.48	2723.02	2732.85	2742.88	2753.04	2763.27
	2773.45	2782.79	2792.37	2802.12	2811.97	2821.88
CH_2CH_2	834.36	836.74	839.11	841.46	843.80	846.13
	951.19	956.86	960.86	964.80	968.70	972.57
	952.81	955.69	960.12	964.49	968.82	973.09
	1030.20	1033.97	1037.59	1041.09	1044.49	1047.79
	1210.23	1214.18	1218.11	1222.03	1225.93	1229.82
	1346.42	1350.46	1354.44	1358.38	1362.27	1366.13
	1429.96	1434.69	1439.41	1444.13	1448.83	1453.53
	1634.46	1639.92	1645.26	1650.50	1655.65	1660.72
	2978.79	2986.09	2993.37	3000.62	3007.83	3015.02
	3016.63	3024.12	3031.57	3038.99	3046.37	3053.72
	3035.30	3042.77	3050.20	3057.60	3064.96	3072.29
	3065.35	3072.79	3080.19	3087.56	3094.90	3102.20
cyclo-C_2H_4O	815.10	817.80	820.47	823.10	825.69	828.26
	802.75	806.62	810.36	813.99	817.52	820.97
	860.38	863.15	865.86	868.52	871.13	873.71
	1019.21	1023.13	1027.02	1030.87	1034.69	1038.48
	1119.56	1124.52	1129.42	1134.29	1139.12	1143.91
	1132.06	1136.87	1141.64	1146.39	1151.12	1155.82
	1139.42	1143.61	1147.75	1151.84	1155.90	1159.93
	1148.68	1152.61	1156.52	1160.41	1164.29	1168.14
	1268.16	1272.11	1276.01	1279.87	1283.70	1287.50
	1457.30	1462.39	1467.46	1472.51	1477.53	1482.54
	1490.03	1495.50	1500.92	1506.31	1511.67	1517.01
	2931.08	2939.23	2947.30	2955.31	2963.26	2971.15
	2961.01	2969.22	2977.36	2985.45	2993.47	3001.44
	2996.31	3004.75	3013.11	3021.40	3029.61	3037.75
	3013.63	3022.10	3030.48	3038.79	3047.03	3055.19
cyclo-C_2H_4NH	783.57	786.34	789.07	791.77	794.44	797.08
	821.18	824.32	827.42	830.47	833.47	836.44
	851.31	853.99	856.63	859.21	861.76	864.26
	917.56	920.67	923.76	926.81	929.85	932.87
	999.00	1002.07	1005.09	1008.08	1011.03	1013.94
	1089.60	1093.84	1098.06	1102.27	1106.45	1110.62
	1093.16	1097.74	1102.29	1106.83	1111.34	1115.84
	1121.75	1125.72	1129.68	1133.61	1137.53	1141.43
	1207.99	1212.29	1216.53	1220.70	1224.83	1228.91

	1234.09	1238.32	1242.51	1246.67	1250.80	1254.91
	1260.34	1263.96	1267.55	1271.10	1274.62	1278.11
	1452.21	1457.22	1462.22	1467.19	1472.14	1477.07
	1478.96	1484.30	1489.61	1494.88	1500.12	1505.35
	2943.58	2951.24	2958.83	2966.35	2973.81	2981.20
	2973.52	2981.27	2988.94	2996.55	3004.10	3011.59
	3007.31	3015.15	3022.92	3030.61	3038.24	3045.80
	3025.07	3032.96	3040.77	3048.51	3056.18	3063.80
	3225.72	3237.49	3249.04	3260.40	3271.59	3282.62
CH_2CCHCl	350.55	351.97	353.36	354.74	356.12	357.48
	489.02	491.62	494.05	496.33	498.47	500.50
	590.34	593.54	596.48	599.20	601.74	604.12
	724.10	727.90	731.47	734.83	738.03	741.08
	838.75	843.97	848.87	853.51	857.92	862.15
	912.47	917.40	922.20	926.88	931.46	935.96
	998.17	1002.10	1005.96	1009.75	1013.48	1017.17
	1105.79	1109.39	1112.88	1116.29	1119.61	1122.86
	1235.48	1240.40	1245.20	1249.90	1254.51	1259.03
	1423.81	1429.02	1434.16	1439.25	1444.30	1449.30
	1979.54	1986.42	1993.02	1999.39	2005.56	2011.54
	2951.59	2959.71	2967.76	2975.74	2983.64	2991.49
	2953.12	2961.79	2970.37	2978.88	2987.30	2995.64
	2979.59	2987.82	2996.01	3004.15	3012.24	3020.29
CH_2CHCHO	339.16	340.10	341.03	342.03	342.92	343.74
	565.74	567.36	568.92	570.44	571.89	573.28
	619.33	621.71	623.99	626.27	628.38	630.34
	917.39	919.70	922.06	924.47	926.84	929.18
	976.49	982.51	988.19	993.63	998.78	1003.67
	992.95	999.48	1005.56	1011.29	1016.66	1021.68
	1007.59	1013.00	1018.05	1022.81	1027.20	1031.28
	1139.93	1143.62	1147.29	1150.94	1154.52	1158.04
	1266.09	1270.51	1274.89	1279.26	1283.55	1287.77
	1339.71	1345.96	1351.94	1357.72	1363.25	1368.58
	1413.81	1419.39	1424.86	1430.25	1435.51	1440.65
	1611.96	1619.65	1626.93	1633.87	1640.47	1646.79
	1687.55	1699.05	1709.70	1719.61	1728.85	1737.53
	2647.60	2657.86	2668.23	2678.68	2689.14	2699.57
	2967.68	2975.33	2982.99	2990.66	2998.30	3005.90
	2960.59	2968.69	2976.73	2984.74	2992.69	3000.57
	3011.08	3018.52	3025.94	3033.37	3040.77	3048.13
cyclo-C_3H_6	765.87	768.09	770.31	772.50	774.68	776.84
	760.82	763.13	765.42	767.70	769.96	772.21
	871.07	873.38	875.68	877.96	880.23	882.48
	852.78	854.82	856.82	858.79	860.73	862.65
	852.45	854.52	856.55	858.55	860.52	862.46
	1041.60	1045.68	1049.75	1053.79	1057.82	1061.84
	1035.84	1040.01	1044.16	1048.28	1052.40	1056.50
	1072.64	1076.97	1081.28	1085.57	1089.86	1094.13
	1124.50	1128.32	1132.12	1135.90	1139.67	1143.42
	1184.73	1188.63	1192.52	1196.39	1200.25	1204.08
	1184.42	1188.35	1192.25	1196.13	1200.00	1203.85
	1189.86	1192.37	1194.87	1197.35	1199.81	1202.26
	1433.37	1438.19	1442.99	1447.79	1452.57	1457.35
	1431.69	1436.56	1441.41	1446.25	1451.08	1455.90

	1477.47	1482.69	1487.90	1493.07	1498.23	1503.36
	2971.20	2978.24	2985.22	2992.17	2999.07	3005.94
	2979.93	2986.92	2993.87	3000.80	3007.66	3014.48
	3010.08	3017.23	3024.34	3031.40	3038.42	3045.39
	3031.35	3038.53	3045.64	3052.71	3059.73	3066.70
	3031.87	3039.03	3046.14	3053.22	3060.22	3067.17
	3073.54	3080.73	3087.86	3094.94	3101.97	3108.95
$CH_2CHCHCH_2$	519.66	520.90	522.11	523.30	524.48	525.64
	585.57	587.26	588.93	590.53	592.07	593.59
	782.95	786.50	789.86	793.05	796.09	799.01
	896.10	897.81	899.52	901.24	902.96	904.69
	939.29	944.22	949.02	953.69	958.25	962.73
	941.72	946.67	951.50	956.22	960.83	965.37
	980.90	985.87	990.68	995.34	999.88	1004.31
	995.86	999.15	1002.40	1005.63	1008.83	1012.01
	1036.04	1040.41	1044.63	1048.72	1052.71	1056.61
	1198.92	1202.01	1205.10	1208.21	1211.31	1214.42
	1280.25	1284.85	1289.35	1293.80	1298.20	1302.52
	1285.10	1289.61	1294.08	1298.51	1302.90	1307.27
	1376.57	1381.37	1386.11	1390.79	1395.42	1400.01
	1432.41	1437.23	1442.05	1446.84	1451.62	1456.39
	1592.66	1598.99	1605.10	1611.03	1616.79	1622.41
	1632.72	1640.61	1648.21	1655.55	1662.65	1669.54
	2966.15	2974.58	2982.99	2991.38	2999.74	3008.09
	2986.22	2999.53	3008.80	3015.82	3021.91	3027.79
	3004.99	3013.15	3021.32	3029.49	3037.65	3045.78
	2990.04	2992.28	2992.73	2993.28	2994.80	2997.51
	3057.41	3064.70	3071.97	3079.21	3086.42	3093.60
	3052.73	3060.18	3067.60	3074.99	3082.34	3089.67
$^1CH_2$	1049.31	1099.53	1148.78	1193.35	1231.29	1262.45
	2830.85	2828.59	2828.36	2830.07	2833.49	2838.33
	2898.92	2884.94	2874.16	2866.81	2862.71	2861.46
$^3CH_2$	912.07	919.04	925.90	932.64	939.30	945.87
	3012.74	3020.31	3027.75	3035.08	3042.31	3049.46
	3200.66	3207.79	3214.81	3221.74	3228.59	3235.37
HCO	1015.24	1027.35	1037.63	1046.42	1054.03	1060.71
	1877.04	1884.71	1892.09	1899.22	1906.14	1912.87
	2389.56	2393.01	2397.88	2403.85	2410.66	2418.11

Table S10. TOSH 2MR vibrational frequencies in cm^{-1} for the modified F1 anharmonic test set calculated using the 6-311++G(d,p) basis set and modified TAO-B3LYP functional with different amounts of exact Hartree-Fock exchange parameterized between 22-32%.

Molecule	22% HF	24% HF	26% HF	28% HF	30% HF	32% HF
$NClF_2$	353.21	355.94	358.55	361.05	363.46	365.78
	359.18	363.47	367.50	371.31	374.95	378.44
	557.12	560.86	564.45	567.91	571.23	574.45
	674.94	681.17	687.04	692.62	697.96	703.11
	823.92	833.73	843.41	852.96	862.40	871.71
	937.69	944.09	950.46	956.79	963.09	969.36
ClF_3	373.29	378.64	383.52	388.03	392.24	396.20
	480.55	484.05	487.42	490.67	493.84	496.94
	657.98	661.13	663.81	666.12	668.12	669.90
	658.65	669.57	679.28	688.02	695.97	703.31
HOCl	691.28	696.96	702.09	706.78	711.16	715.27
	1203.48	1209.21	1214.67	1219.90	1224.97	1229.89
	3575.37	3589.01	3602.48	3615.80	3628.96	3642.00
ClNO	300.57	305.24	309.15	312.41	315.09	317.30
	570.66	577.84	584.06	589.46	594.16	598.30
	1909.04	1915.82	1922.51	1929.09	1935.53	1941.85
$ClNO_2$	352.39	357.92	362.97	367.65	372.06	376.26
	406.19	409.70	412.96	416.04	418.97	421.77
	652.52	658.43	663.77	668.65	673.17	677.39
	800.61	805.14	809.40	813.45	817.34	821.09
	1337.82	1342.51	1347.03	1351.43	1355.72	1359.92
	1729.26	1732.39	1735.32	1738.07	1740.64	1743.07
ClSN	365.53	370.80	375.49	379.69	383.45	386.86
	1344.19	1350.69	1356.63	1362.07	1367.04	1371.57
NCl_2F	331.25	335.17	338.83	342.25	345.48	348.55
	416.43	420.55	424.35	427.90	431.23	434.36
	602.86	607.21	611.18	614.86	618.30	621.55
	640.55	650.22	659.43	668.25	676.73	684.94
	821.24	829.46	837.62	845.72	853.77	861.75
Cl_2O	580.91	598.38	613.82	637.06	640.74	644.18
	623.92	628.74	633.08	627.62	640.10	651.51
$SOCl_2$	312.15	315.45	318.53	321.41	324.15	326.74
	403.81	408.32	412.49	416.38	420.04	423.51
	449.37	453.56	457.42	461.00	464.34	467.49
	1214.97	1220.12	1225.02	1229.71	1234.19	1238.48
SCl_2	441.37	448.89	455.55	461.50	466.84	471.69
	477.18	481.16	484.73	487.98	490.95	493.69
HOF	930.73	937.86	944.50	950.78	956.78	962.58
	1366.06	1373.26	1380.23	1387.03	1393.68	1400.22
	3535.40	3549.01	3562.52	3575.94	3589.26	3602.49
ONF	496.19	499.59	502.77	505.78	508.67	511.48
	758.79	763.14	767.22	771.09	774.81	778.44
	1932.98	1939.39	1945.55	1951.46	1957.17	1962.69
NSF	341.32	344.25	347.11	349.92	352.68	355.42
	573.32	576.73	580.01	583.19	586.32	589.39
	1385.28	1391.14	1396.52	1401.48	1406.05	1410.26
F_2NH	495.20	498.40	501.51	504.54	507.51	510.40
	857.44	866.21	874.76	883.11	891.28	899.29

	983.68	989.48	995.20	1000.82	1006.37	1011.86
	1298.16	1302.55	1306.87	1311.13	1315.34	1319.51
	1425.53	1432.91	1440.18	1447.36	1454.44	1461.44
	3148.00	3162.44	3176.72	3190.86	3204.84	3218.67
N_2F_2	361.07	363.36	365.57	367.71	369.79	371.81
	420.84	423.11	425.34	427.51	429.63	431.72
	601.56	605.16	608.63	612.01	615.31	618.53
	975.02	981.28	987.44	993.50	999.48	1005.38
	1031.02	1038.37	1045.56	1052.60	1059.51	1066.29
	1594.45	1605.52	1616.36	1627.01	1637.47	1647.77
F_2O	470.96	475.34	479.42	483.28	486.96	490.50
	837.21	850.39	862.57	874.02	884.89	895.33
	985.40	991.87	998.01	1003.89	1009.58	1015.13
F_2SO	323.02	326.18	329.29	332.35	335.35	338.31
	354.37	356.79	359.18	361.54	363.86	366.16
	476.53	480.25	483.91	487.51	491.05	494.55
	650.62	654.92	659.18	663.40	667.59	671.76
	724.03	728.43	732.77	737.06	741.30	745.50
	1296.02	1301.35	1306.53	1311.57	1316.48	1321.27
S_2F_2	567.38	566.10	563.94	562.65	561.38	560.16
	604.45	610.94	616.66	622.78	628.74	634.56
	643.64	649.74	655.00	660.78	666.42	671.93
HNO_3	364.16	367.09	369.78	372.25	374.52	376.61
	584.87	588.47	591.97	595.38	598.72	601.98
	645.07	650.49	655.75	660.85	665.78	670.57
	765.51	769.84	773.99	778.00	781.88	785.65
	891.06	897.59	904.07	910.49	916.86	923.18
	1296.09	1302.42	1308.59	1314.61	1320.50	1326.27
	1337.28	1343.68	1349.97	1356.16	1362.28	1368.34
	1727.26	1733.35	1739.23	1744.93	1750.46	1755.84
	3462.76	3475.78	3488.69	3501.48	3514.18	3526.77
HN_3	526.61	528.05	529.37	530.59	531.73	532.79
	585.74	587.72	589.64	591.51	593.32	595.08
	1157.44	1161.80	1165.98	1169.96	1173.73	1177.27
	1285.58	1289.49	1293.40	1297.31	1301.24	1305.19
	2225.70	2232.78	2239.70	2246.49	2253.16	2259.72
	3299.85	3309.37	3318.75	3328.01	3337.18	3346.26
H_2O	1552.19	1556.86	1561.53	1566.21	1570.89	1575.57
	3723.11	3735.03	3746.84	3758.56	3770.19	3781.74
	3741.93	3753.61	3765.18	3776.66	3788.06	3799.38
H_2O_2	204.47	206.02	207.23	208.19	208.95	209.55
	918.00	924.83	931.36	937.68	943.81	949.80
	1266.52	1272.46	1278.22	1283.83	1289.31	1294.68
	1433.82	1439.89	1445.86	1451.74	1457.54	1463.27
	3538.97	3552.41	3565.73	3578.94	3592.02	3605.00
	3614.48	3627.87	3641.13	3654.27	3667.29	3680.22
H_2S	1185.09	1189.55	1193.94	1198.34	1202.69	1207.03
	2628.81	2636.93	2644.89	2652.76	2660.52	2668.18
	2598.52	2606.68	2614.66	2622.49	2630.19	2637.76
H_2S_2	388.51	389.71	390.65	391.38	391.95	392.38
	465.46	468.92	472.08	474.99	477.70	480.24
	873.99	878.53	882.86	887.01	891.00	894.86
	880.17	884.40	888.44	892.32	896.05	899.66
	2552.70	2561.81	2570.83	2579.79	2588.67	2597.47

	2513.24	2522.40	2531.47	2540.47	2549.39	2558.23
NO_2	751.13	755.28	759.03	762.44	765.60	768.54
	1382.04	1389.57	1396.76	1403.67	1410.33	1416.77
	1652.21	1658.69	1664.70	1670.28	1675.48	1680.34
N_2O	586.80	589.07	591.28	593.45	595.58	597.67
	586.80	589.07	591.28	593.45	595.58	597.67
	1311.45	1314.14	1316.64	1318.94	1321.05	1322.97
	2302.00	2309.49	2316.89	2324.22	2331.49	2338.71
SO_2	502.42	505.83	509.11	512.29	515.38	518.39
	1127.20	1135.16	1142.83	1150.22	1157.40	1164.37
	1299.18	1307.67	1315.87	1323.81	1331.52	1339.04
O_3	725.73	733.01	739.42	745.14	750.29	754.99
	1154.07	1171.48	1187.40	1202.02	1215.50	1227.96
	1224.38	1237.34	1249.22	1260.21	1270.47	1280.12
COClF	407.75	408.98	410.19	411.38	412.56	413.73
	492.56	494.47	496.34	498.18	500.00	501.79
	662.26	665.13	667.93	670.65	673.32	675.95
	756.34	759.28	762.18	765.03	767.84	770.62
	1050.75	1056.16	1061.50	1066.77	1071.98	1077.12
	1899.49	1905.46	1911.33	1917.11	1922.81	1928.44
ClCN	388.30	389.87	391.42	392.94	394.44	395.93
	388.30	389.87	391.42	392.94	394.44	395.93
	735.47	737.22	738.92	740.57	742.18	743.76
	2285.42	2294.08	2302.66	2311.15	2319.56	2327.89
$COCl_2$	440.04	441.96	443.84	445.68	447.50	449.28
	556.70	558.66	560.55	562.40	564.20	565.97
	578.74	581.31	583.75	586.09	588.34	590.52
	814.31	818.52	822.64	826.69	830.66	834.59
	1852.89	1858.82	1864.61	1870.28	1875.86	1881.34
FCN	482.14	484.29	486.44	488.57	490.68	492.78
	482.14	484.29	486.44	488.57	490.68	492.78
	1077.28	1080.66	1083.99	1087.26	1090.49	1093.67
	2384.83	2393.31	2401.71	2410.04	2418.29	2426.47
COF_2	573.21	575.17	577.12	579.05	580.96	582.85
	613.91	616.32	618.70	621.06	623.38	625.69
	768.03	771.47	774.87	778.23	781.54	784.83
	954.78	958.63	962.43	966.18	969.89	973.55
	1186.28	1192.39	1198.42	1204.36	1210.23	1216.03
	1954.13	1960.12	1966.02	1971.85	1977.60	1983.29
CSF_2	417.17	418.72	420.22	421.67	423.08	424.45
	522.71	524.54	526.32	528.06	529.75	531.42
	608.34	612.12	615.66	619.00	622.16	625.18
	787.32	790.37	793.33	796.19	798.98	801.70
	1148.25	1154.17	1160.03	1165.85	1171.61	1177.30
	1341.09	1346.14	1351.00	1355.69	1360.23	1364.61
COS	509.80	512.09	514.24	516.34	518.38	520.37
	509.80	512.09	514.24	516.34	518.38	520.37
	871.24	872.78	874.22	875.57	876.85	878.04
	2088.80	2094.34	2099.78	2105.14	2110.42	2115.63
CO_2	668.31	671.29	674.25	677.18	680.09	682.98
	668.31	671.29	674.25	677.18	680.09	682.98
	1369.07	1373.75	1378.38	1382.95	1387.47	1391.93
	2383.34	2388.38	2393.34	2398.22	2403.01	2407.73
CS_2	393.60	395.54	397.38	399.14	400.82	402.43

	393.60	395.54	397.38	399.14	400.82	402.43
	672.23	674.30	676.31	678.25	680.13	681.95
	1533.55	1536.34	1538.92	1541.32	1543.53	1545.59
HCN	738.42	742.11	745.81	749.56	753.40	757.03
	738.42	742.11	745.81	749.56	753.40	757.03
	2179.52	2186.72	2193.86	2200.94	2207.95	2214.91
	3285.83	3293.93	3301.97	3309.96	3317.90	3325.78
HNCO	596.87	597.59	598.28	598.94	599.58	600.20
	628.40	631.07	633.72	636.34	638.95	641.53
	775.58	778.22	780.88	783.57	786.29	789.04
	1332.33	1336.24	1340.08	1343.86	1347.58	1351.25
	2304.17	2308.55	2312.83	2317.02	2321.12	2325.14
	3496.77	3505.62	3514.35	3522.98	3531.51	3539.94
H_2CO	1171.20	1176.73	1182.08	1187.26	1192.33	1197.29
	1246.01	1250.35	1254.59	1258.75	1262.84	1266.86
	1510.89	1515.88	1520.79	1525.65	1530.46	1535.23
	1796.67	1802.93	1808.99	1814.88	1820.63	1826.26
	2794.27	2803.66	2813.01	2822.31	2831.57	2840.78
	2776.31	2786.73	2797.08	2807.34	2817.51	2827.61
HCOOH	454.89	452.22	449.59	447.01	444.48	441.98
	665.30	667.57	669.81	672.01	674.18	676.33
	1015.74	1020.69	1025.56	1030.38	1035.13	1039.84
	1085.72	1089.54	1093.28	1096.96	1100.57	1104.13
	1242.27	1246.58	1250.85	1255.09	1259.29	1263.47
	1400.20	1405.59	1410.94	1416.25	1421.52	1426.75
	1838.69	1844.58	1850.35	1856.02	1861.60	1867.10
	2797.46	2807.03	2816.54	2826.00	2835.42	2844.80
	3538.09	3552.12	3565.93	3579.54	3592.96	3606.21
C_2Cl_2	261.14	268.78	276.08	283.13	289.95	296.57
	261.14	268.78	276.08	283.13	289.95	296.57
	474.11	475.04	475.94	476.81	477.64	478.46
	981.06	983.02	984.89	986.68	988.41	990.09
	2316.09	2323.36	2330.53	2337.62	2344.62	2351.55
C_2N_2	556.08	557.72	559.35	560.99	562.63	564.27
	556.08	557.72	559.35	560.99	562.63	564.27
	881.78	882.90	884.02	885.15	886.27	887.39
	2246.10	2254.89	2263.55	2272.09	2280.53	2288.87
	2421.05	2431.03	2440.83	2450.47	2459.95	2469.30
HCCCl	371.31	371.87	372.49	373.17	373.89	374.64
	371.31	371.87	372.49	373.17	373.89	374.65
	668.09	671.97	675.83	679.67	683.49	687.30
	668.09	671.97	675.83	679.67	683.49	687.30
	743.83	745.18	746.49	747.78	749.03	750.26
	2165.08	2171.65	2178.13	2184.53	2190.85	2197.10
	3291.83	3299.43	3306.98	3314.47	3321.91	3329.30
HCCF	404.29	407.60	410.84	414.01	417.14	420.21
	404.29	407.60	410.84	414.01	417.14	420.21
	582.60	587.54	592.45	597.34	602.20	606.94
	582.60	587.54	592.45	597.34	602.21	606.94
	1064.86	1067.38	1069.86	1072.32	1074.75	1077.16
	2287.03	2293.47	2299.84	2306.15	2312.40	2318.59
	3305.09	3312.61	3320.07	3327.49	3334.86	3342.19
HCCH	752.57	753.86	755.23	756.78	758.38	760.06
	752.57	753.86	755.23	756.78	758.38	760.06

	804.93	807.77	810.60	813.44	816.27	819.10
	804.93	807.77	810.60	813.44	816.27	819.10
	2039.09	2044.64	2050.13	2055.56	2060.93	2066.25
	3244.78	3252.07	3259.33	3266.55	3273.73	3280.88
	3394.92	3402.43	3409.91	3417.34	3424.73	3432.08
trans-CHClCHCl	348.42	349.35	350.25	351.14	352.00	352.86
	803.78	807.97	812.06	816.07	820.02	823.90
	796.23	798.24	800.21	802.13	804.01	805.87
	840.32	842.78	845.19	847.58	849.93	852.25
	931.67	936.14	940.51	944.81	949.05	953.25
	1217.94	1222.31	1226.65	1230.96	1235.24	1239.50
	1295.33	1299.92	1304.46	1308.96	1313.44	1317.89
	1620.35	1626.43	1632.35	1638.14	1643.82	1649.39
	3057.40	3065.31	3073.17	3081.01	3088.81	3096.58
	3111.12	3119.04	3126.93	3134.78	3142.59	3150.38
cis-CHClCHCl	417.01	418.61	420.13	421.59	423.01	424.38
	566.49	567.87	569.23	570.57	571.88	573.18
	701.16	702.75	704.29	705.81	707.29	708.75
	737.44	740.67	743.83	746.94	750.01	753.04
	837.59	839.95	842.26	844.54	846.80	849.02
	923.39	928.45	933.42	938.31	943.14	947.91
	1212.61	1216.91	1221.17	1225.39	1229.59	1233.76
	1306.33	1311.12	1315.86	1320.57	1325.26	1329.93
	1621.71	1627.69	1633.54	1639.27	1644.90	1650.43
	3031.49	3039.26	3046.99	3054.69	3062.35	3069.98
	3104.44	3112.24	3120.00	3127.72	3135.41	3143.06
CH_2CCl_2	300.75	301.46	302.15	302.83	303.50	304.15
	391.65	392.67	393.68	394.68	395.66	396.62
	477.84	479.37	480.82	482.21	483.55	484.84
	593.73	595.29	596.81	598.29	599.75	601.18
	717.68	720.60	723.45	726.25	728.99	731.68
	764.11	767.36	770.55	773.69	776.77	779.81
	939.03	943.84	948.59	953.30	957.98	962.63
	1093.98	1098.03	1102.05	1106.04	1110.00	1113.94
	1392.61	1397.50	1402.36	1407.20	1412.01	1416.79
	1636.89	1642.71	1648.42	1654.03	1659.56	1665.01
	3052.19	3059.42	3066.62	3073.78	3080.92	3088.02
	3081.60	3088.91	3096.19	3103.44	3110.65	3117.83
cis-CHFCHF	508.03	509.94	511.79	513.60	515.36	517.08
	766.70	768.85	770.97	773.07	775.15	777.22
	801.11	804.69	808.22	811.71	815.16	818.58
	893.64	898.60	903.49	908.31	913.08	917.78
	1009.41	1012.26	1015.07	1017.85	1020.59	1023.30
	1111.42	1114.47	1117.48	1120.46	1123.41	1126.33
	1274.38	1278.57	1282.74	1286.88	1291.00	1295.10
	1371.59	1376.46	1381.30	1386.11	1390.88	1395.63
	1732.17	1738.38	1744.49	1750.51	1756.45	1762.32
	3052.33	3060.69	3068.99	3077.24	3085.44	3093.59
	3126.69	3135.05	3143.36	3151.62	3159.82	3167.97
trans-CHFCHF	322.48	323.64	324.78	325.92	327.04	328.15
	331.24	332.58	333.89	335.17	336.42	337.65
	550.13	551.70	553.25	554.79	556.31	557.81
	833.27	837.81	842.28	846.68	851.01	855.29
	908.73	913.17	917.55	921.87	926.14	930.37

	1123.51	1126.78	1130.01	1133.20	1136.36	1139.49
	1133.18	1136.16	1139.10	1142.01	1144.89	1147.73
	1280.13	1284.71	1289.26	1293.78	1298.28	1302.76
	1297.03	1301.50	1305.93	1310.34	1314.71	1319.06
	1719.76	1726.04	1732.22	1738.29	1744.28	1750.19
	3062.06	3070.33	3078.57	3086.75	3094.90	3103.00
	3119.19	3127.46	3135.69	3143.88	3152.02	3160.11
OCHCHO	347.23	348.35	349.52	350.74	352.00	353.30
	547.78	549.70	551.61	553.50	555.37	557.22
	802.98	806.96	810.85	814.66	818.41	822.09
	1034.95	1039.67	1044.19	1048.56	1052.80	1056.91
	1063.39	1069.32	1074.91	1080.20	1085.26	1090.12
	1319.99	1325.56	1330.97	1336.22	1341.36	1346.39
	1360.88	1366.41	1371.77	1377.00	1382.09	1387.08
	1776.42	1783.67	1790.58	1797.21	1803.61	1809.81
	1784.19	1792.95	1801.27	1809.22	1816.87	1824.27
	2773.52	2783.76	2793.96	2804.10	2814.18	2824.18
	2831.81	2841.73	2851.61	2861.45	2871.23	2880.95
CH_2CH_2	848.46	850.77	853.08	855.38	857.67	859.95
	976.40	980.21	983.99	987.75	991.49	995.21
	977.33	981.53	985.70	989.83	993.94	998.02
	1051.03	1054.20	1057.31	1060.39	1063.42	1066.41
	1233.70	1237.56	1241.42	1245.26	1249.10	1252.93
	1369.96	1373.75	1377.52	1381.26	1384.98	1388.68
	1458.21	1462.90	1467.57	1472.24	1476.90	1481.56
	1665.72	1670.67	1675.56	1680.41	1685.22	1689.99
	3022.18	3029.31	3036.41	3043.49	3050.53	3057.55
	3061.03	3068.32	3075.57	3082.79	3089.98	3097.14
	3079.58	3086.84	3094.06	3101.25	3108.40	3115.52
	3109.46	3116.70	3123.90	3131.06	3138.20	3145.30
cyclo-C_2H_4O	830.79	833.30	835.79	838.25	840.69	843.11
	824.35	827.66	830.91	834.10	837.23	840.32
	876.25	878.76	881.25	883.70	886.13	888.54
	1042.26	1046.00	1049.73	1053.44	1057.13	1060.80
	1148.68	1153.42	1158.13	1162.83	1167.50	1172.15
	1160.51	1165.18	1169.83	1174.47	1179.10	1183.71
	1163.93	1167.90	1171.85	1175.77	1179.67	1183.56
	1171.99	1175.82	1179.64	1183.44	1187.24	1191.02
	1291.28	1295.04	1298.77	1302.48	1306.18	1309.86
	1487.53	1492.51	1497.47	1502.42	1507.36	1512.29
	1522.32	1527.61	1532.88	1538.13	1543.37	1548.59
	2978.98	2986.76	2994.49	3002.17	3009.81	3017.39
	3009.35	3017.22	3025.03	3032.80	3040.52	3048.20
	3045.83	3053.84	3061.78	3069.67	3077.49	3085.26
	3063.29	3071.32	3079.29	3087.20	3095.05	3102.85
cyclo-C_2H_4NH	799.68	802.26	804.81	807.34	809.84	812.33
	839.36	842.25	845.09	847.90	850.68	853.41
	866.74	869.17	871.58	873.96	876.31	878.64
	935.86	938.84	941.81	944.77	947.70	950.63
	1016.83	1019.69	1022.52	1025.34	1028.13	1030.91
	1114.79	1118.94	1123.09	1127.22	1131.35	1135.48
	1120.33	1124.80	1129.25	1133.69	1138.12	1142.54
	1145.32	1149.20	1153.07	1156.93	1160.79	1164.63
	1232.95	1236.94	1240.90	1244.82	1248.71	1252.56

	1259.00	1263.08	1267.14	1271.18	1275.22	1279.25
	1281.58	1285.03	1288.46	1291.88	1295.29	1298.70
	1481.99	1486.90	1491.79	1496.67	1501.55	1506.41
	1510.54	1515.72	1520.88	1526.03	1531.16	1536.27
	2988.55	2995.85	3003.09	3010.29	3017.45	3024.57
	3019.03	3026.42	3033.77	3041.06	3048.32	3055.54
	3053.30	3060.75	3068.14	3075.48	3082.77	3090.01
	3071.35	3078.85	3086.29	3093.68	3101.02	3108.32
	3293.52	3304.29	3314.94	3325.48	3335.92	3346.27
CH_2CCHCl	358.84	360.19	361.53	362.86	364.18	365.48
	502.45	504.31	506.11	507.84	509.52	511.16
	606.38	608.53	610.58	612.56	614.46	616.32
	744.01	746.84	749.58	752.24	754.84	757.37
	866.21	870.13	873.92	877.62	881.22	884.75
	940.38	944.74	949.05	953.31	957.52	961.71
	1020.82	1024.43	1028.01	1031.56	1035.09	1038.60
	1126.05	1129.19	1132.28	1135.33	1138.33	1141.30
	1263.50	1267.91	1272.27	1276.59	1280.86	1285.10
	1454.26	1459.20	1464.11	1468.99	1473.85	1478.69
	2017.36	2023.04	2028.60	2034.05	2039.40	2044.67
	2999.28	3007.02	3014.70	3022.33	3029.92	3037.46
	3003.92	3012.13	3020.26	3028.34	3036.36	3044.32
	3028.31	3036.27	3044.20	3052.08	3059.93	3067.75
CH_2CHCHO	344.62	345.50	346.37	347.23	348.07	348.94
	574.65	576.00	577.32	578.62	579.90	581.17
	632.34	634.28	636.18	638.05	639.89	641.73
	931.56	933.93	936.30	938.66	941.01	943.36
	1008.43	1013.04	1017.53	1021.92	1026.22	1030.48
	1026.51	1031.13	1035.55	1039.80	1043.86	1047.78
	1035.19	1038.88	1042.38	1045.69	1048.84	1051.84
	1161.54	1165.00	1168.43	1171.82	1175.18	1178.53
	1291.99	1296.18	1300.34	1304.47	1308.57	1312.66
	1373.80	1378.90	1383.89	1388.80	1393.63	1398.41
	1445.74	1450.77	1455.73	1460.63	1465.49	1470.32
	1652.88	1658.77	1664.48	1670.05	1675.49	1680.82
	1745.74	1753.56	1761.05	1768.26	1775.24	1782.02
	2709.98	2720.34	2730.63	2740.86	2751.00	2761.07
	3013.50	3021.06	3028.60	3036.11	3043.57	3051.01
	3008.42	3016.22	3023.98	3031.70	3039.38	3047.03
	3055.48	3062.81	3070.12	3077.40	3084.65	3091.88
cyclo-C_3H_6	778.99	781.13	783.26	785.37	787.48	789.57
	774.44	776.66	778.87	781.06	783.23	785.40
	884.72	886.95	889.18	891.39	893.59	895.78
	864.54	866.41	868.26	870.09	871.90	873.69
	864.37	866.27	868.13	869.98	871.81	873.61
	1065.85	1069.85	1073.84	1077.81	1081.78	1085.74
	1060.59	1064.67	1068.74	1072.79	1076.84	1080.88
	1098.39	1102.63	1106.87	1111.09	1115.30	1119.51
	1147.16	1150.89	1154.62	1158.33	1162.04	1165.73
	1207.91	1211.72	1209.49	1211.87	1214.24	1216.59
	1207.69	1211.52	1215.53	1219.32	1223.10	1226.87
	1204.68	1207.09	1215.33	1219.14	1222.93	1226.71
	1462.11	1466.87	1471.62	1476.36	1481.10	1485.83
	1460.70	1465.50	1470.29	1475.08	1479.85	1484.62

	1508.48	1513.58	1518.66	1523.73	1528.78	1533.82
	3012.76	3019.56	3026.32	3033.05	3039.74	3046.40
	3021.26	3028.00	3034.71	3041.38	3048.02	3054.62
	3052.33	3059.24	3066.11	3072.94	3079.74	3086.51
	3073.63	3080.52	3087.36	3094.16	3100.91	3107.63
	3074.09	3080.95	3087.78	3094.56	3101.30	3108.00
	3115.89	3122.79	3129.65	3136.46	3143.24	3149.98
$CH_2CHCHCH_2$	526.78	532.24	533.37	534.48	535.58	536.68
	595.01	597.17	598.60	600.00	601.39	602.75
	801.81	804.35	806.99	809.55	812.05	814.50
	906.42	914.27	915.99	917.72	919.45	921.17
	967.11	971.87	976.11	980.30	984.42	988.50
	969.81	974.63	978.95	983.20	987.41	991.56
	1008.64	1012.85	1017.02	1021.13	1025.18	1029.18
	1015.16	1026.90	1030.02	1033.12	1036.20	1039.26
	1060.43	1064.26	1067.95	1071.59	1075.18	1078.74
	1217.53	1222.71	1225.86	1229.00	1232.14	1235.28
	1306.80	1313.27	1317.47	1321.63	1325.76	1329.87
	1311.60	1318.57	1322.86	1327.13	1331.38	1335.60
	1404.55	1413.50	1417.96	1422.37	1426.76	1431.12
	1461.14	1471.05	1475.79	1480.51	1485.21	1489.91
	1627.90	1634.10	1639.40	1644.60	1649.72	1654.77
	1676.22	1682.55	1688.89	1695.09	1701.16	1707.12
	3016.42	3023.13	3031.36	3039.58	3047.78	3055.96
	3033.72	3036.49	3042.67	3049.00	3055.44	3061.97
	3053.84	3058.88	3066.82	3074.64	3082.29	3089.78
	3001.22	3004.50	3009.70	3015.36	3021.36	3027.61
	3100.75	3104.40	3111.50	3118.57	3125.61	3132.61
	3096.96	3100.82	3108.06	3115.26	3122.44	3129.58
$^{1}CH_2$	1287.56	1307.74	1324.09	1337.51	1348.74	1358.32
	2844.24	2850.96	2858.27	2866.01	2874.05	2882.29
	2862.53	2865.42	2869.68	2875.02	2881.13	2887.82
$^{3}CH_2$	952.36	958.79	965.15	971.46	977.71	983.91
	3056.55	3063.57	3070.54	3077.46	3084.33	3091.17
	3242.09	3248.75	3255.37	3261.94	3268.48	3274.97
HCO	1066.69	1072.11	1077.10	1081.76	1086.16	1090.37
	1919.45	1925.89	1932.21	1938.42	1944.53	1950.54
	2426.03	2434.30	2442.82	2451.49	2460.30	2469.19

Table S11. TOSH 2MR vibrational frequencies in cm^{-1} for the modified F1 anharmonic test set calculated using the 6-311++G(d,p) basis set and modified TAO-B3LYP functional with different amounts of exact Hartree-Fock exchange parameterized between 34-40%.

Molecule	34% HF	36% HF	38% HF	40% HF
$NClF_2$	368.03	370.21	372.34	374.41
	381.79	385.03	388.16	391.20
	577.55	580.57	583.49	586.32
	708.09	712.95	717.70	722.35
	880.90	889.97	898.92	907.75
	975.60	981.81	987.98	994.12
ClF_3	394.90	398.56	402.06	405.43
	498.56	501.58	504.56	507.52
	668.46	669.88	671.17	672.37
	708.92	715.40	721.54	727.40
HOCl	719.17	722.91	726.51	730.00
	1234.69	1239.40	1244.03	1248.58
	3654.94	3667.73	3680.43	3693.04
ClNO	319.09	320.52	321.65	322.52
	601.95	605.21	608.14	610.82
	1948.03	1954.06	1959.94	1965.68
$ClNO_2$	380.32	384.28	388.17	392.02
	424.47	427.09	429.64	432.12
	681.38	685.17	688.79	692.28
	824.75	828.34	831.86	835.35
	1364.04	1368.10	1372.11	1376.08
	1745.34	1747.49	1749.51	1751.41
ClSN	389.95	392.79	395.42	397.88
	1375.69	1379.44	1382.82	1385.87
NCl_2F	351.47	354.28	356.97	359.57
	437.31	440.12	442.79	445.34
	624.65	627.63	630.51	633.32
	692.90	700.65	708.21	715.61
	869.66	877.50	885.26	892.94
Cl_2O	647.44	650.55	653.54	653.80
	662.06	671.89	681.13	686.08
$SOCl_2$	329.23	331.63	333.95	336.20
	426.82	430.01	433.09	436.09
	470.48	473.33	476.07	478.72
	1242.59	1246.52	1250.30	1253.93
SCl_2	476.11	480.18	483.95	487.48
	496.25	498.64	500.91	503.07
HOF	968.21	973.71	979.10	984.40
	1406.65	1413.00	1419.27	1425.46
	3615.63	3628.68	3641.65	3654.54
ONF	514.24	516.98	519.72	522.48
	782.01	785.54	789.05	792.56
	1968.02	1973.20	1978.23	1983.13
NSF	358.12	360.79	363.45	366.09
	592.43	595.45	598.46	601.47
	1414.15	1417.74	1421.04	1424.08
F_2NH	513.24	516.02	518.75	521.43
	907.16	914.89	922.48	929.95

	1017.27	1022.63	1027.92	1033.16
	1323.64	1327.74	1331.80	1335.85
	1468.37	1475.21	1481.99	1488.69
	3232.37	3245.92	3259.34	3272.63
N_2F_2	373.80	375.74	377.65	379.53
	433.76	435.77	437.74	439.68
	621.68	624.78	627.82	630.81
	1011.19	1016.94	1022.62	1028.23
	1072.97	1079.54	1086.02	1092.41
	1657.92	1667.94	1677.84	1687.61
F_2O	493.93	497.26	500.52	503.71
	905.42	915.22	924.79	934.16
	1020.56	1025.90	1031.17	1036.38
F_2SO	341.22	344.08	346.90	349.67
	368.44	370.70	372.93	375.15
	498.00	501.41	504.77	508.09
	675.90	680.02	684.12	688.20
	749.67	753.80	757.89	761.96
	1325.95	1330.51	1334.96	1339.32
S_2F_2	558.98	557.87	556.82	555.85
	640.25	645.83	651.29	656.65
	677.32	682.60	687.79	692.87
HNO_3	378.53	380.29	381.91	383.40
	605.18	608.31	611.39	614.41
	675.22	679.72	684.10	688.35
	789.34	792.94	796.49	799.97
	929.45	935.68	941.86	947.99
	1331.93	1337.48	1342.93	1348.29
	1374.35	1380.32	1386.25	1392.15
	1761.09	1766.21	1771.22	1776.11
	3539.27	3551.67	3563.99	3576.22
HN_3	533.78	534.73	535.62	536.47
	596.81	598.49	600.15	601.76
	1180.57	1183.61	1186.37	1188.85
	1309.17	1313.18	1317.23	1321.31
	2266.17	2272.53	2278.81	2285.00
	3355.25	3364.17	3373.04	3381.86
H_2O	1580.25	1584.94	1589.63	1594.33
	3793.20	3804.59	3815.90	3827.14
	3810.61	3821.77	3832.85	3843.86
H_2O_2	210.02	210.40	210.70	210.92
	955.67	961.45	967.13	972.75
	1299.96	1305.16	1310.28	1315.34
	1468.94	1474.54	1480.09	1485.59
	3617.88	3630.66	3643.34	3655.93
	3693.04	3705.76	3718.39	3730.93
H_2S	1211.37	1215.69	1220.01	1224.33
	2675.77	2683.29	2690.76	2698.17
	2645.25	2652.64	2659.96	2667.22
H_2S_2	392.72	392.96	393.15	393.29
	482.64	484.92	487.10	489.20
	898.59	902.22	905.76	909.22
	903.16	906.57	909.88	913.13
	2606.21	2614.88	2623.48	2632.01

	2567.00	2575.70	2584.33	2592.89
NO_2	771.32	773.95	776.46	778.88
	1423.02	1429.09	1435.02	1440.80
	1684.88	1689.11	1693.07	1696.76
N_2O	599.72	601.75	603.74	605.70
	599.72	601.75	603.74	605.70
	1324.70	1326.24	1327.60	1328.76
	2345.87	2352.99	2360.06	2367.09
SO_2	521.33	524.22	527.05	529.84
	1171.17	1177.81	1184.32	1190.70
	1346.39	1353.58	1360.63	1367.55
O_3	759.31	763.34	767.11	770.68
	1239.50	1250.21	1260.13	1269.32
	1289.27	1298.01	1306.40	1314.49
COClF	414.88	416.02	417.15	418.27
	503.57	505.32	507.05	508.77
	678.53	681.07	683.59	686.07
	773.37	776.09	778.78	781.44
	1082.20	1087.22	1092.18	1097.09
	1933.99	1939.49	1944.92	1950.30
ClCN	397.40	398.86	400.32	401.77
	397.40	398.86	400.32	401.77
	745.30	746.81	748.29	749.76
	2336.16	2344.36	2352.49	2360.55
$COCl_2$	451.05	452.78	454.50	456.19
	567.72	569.44	571.14	572.82
	592.64	594.71	596.73	598.72
	838.46	842.28	846.07	849.82
	1886.74	1892.07	1897.33	1902.53
FCN	494.88	496.98	499.07	501.16
	494.88	496.98	499.07	501.16
	1096.80	1099.90	1102.96	1105.99
	2434.58	2442.63	2450.61	2458.54
COF_2	584.72	586.58	588.42	590.25
	627.96	630.22	632.44	634.65
	788.09	791.31	794.52	797.69
	977.16	980.73	984.26	987.76
	1221.75	1227.40	1232.98	1238.50
	1988.91	1994.47	1999.98	2005.42
CSF_2	425.79	427.10	428.38	429.64
	533.05	534.65	536.22	537.77
	628.07	630.85	633.55	636.16
	804.35	806.95	809.49	811.98
	1182.94	1188.52	1194.04	1199.49
	1368.87	1373.01	1377.04	1380.96
COS	522.32	524.23	526.11	527.96
	522.32	524.23	526.11	527.96
	879.16	880.22	881.20	882.12
	2120.76	2125.83	2130.83	2135.77
CO_2	685.86	688.72	691.56	694.39
	685.86	688.72	691.56	694.39
	1396.35	1400.72	1405.03	1409.30
	2412.37	2416.94	2421.43	2425.85
CS_2	403.98	405.48	406.93	408.34

	403.98	405.48	406.93	408.34
	683.73	685.46	687.14	688.79
	1547.49	1549.24	1550.85	1552.37
HCN	760.74	764.51	768.29	772.06
	760.74	764.51	768.29	772.06
	2221.81	2228.66	2235.45	2242.20
	3333.61	3341.41	3349.16	3356.87
HNCO	600.82	601.44	602.06	602.68
	644.09	646.63	649.16	651.67
	791.82	794.64	797.49	800.38
	1354.86	1358.43	1361.94	1365.40
	2329.08	2332.94	2336.73	2340.45
	3548.29	3556.56	3564.76	3572.89
H_2CO	1202.18	1207.01	1211.78	1216.51
	1270.83	1274.77	1278.66	1282.53
	1539.96	1544.67	1549.35	1554.00
	1831.80	1837.25	1842.64	1847.96
	2849.94	2859.05	2868.11	2877.13
	2837.61	2847.52	2857.35	2867.09
HCOOH	439.54	437.14	434.79	432.49
	678.45	680.54	682.61	684.65
	1044.50	1049.12	1053.71	1058.27
	1107.62	1111.06	1114.44	1117.78
	1267.63	1271.76	1275.86	1279.94
	1431.95	1437.11	1442.24	1447.33
	1872.53	1877.89	1883.19	1888.44
	2854.12	2863.40	2872.63	2881.82
	3619.29	3632.22	3644.99	3657.63
C_2Cl_2	409.24	409.23	409.57	410.19
	409.24	409.23	409.57	410.19
	478.37	479.17	479.95	480.72
	989.84	991.48	993.10	994.69
	2333.34	2340.52	2347.62	2354.64
C_2N_2	565.92	567.58	569.24	570.91
	565.92	567.58	569.24	570.91
	888.52	889.64	890.77	891.89
	2297.12	2305.28	2313.35	2321.35
	2478.52	2487.62	2496.61	2505.51
HCCCl	375.44	376.23	377.08	377.95
	375.44	376.23	377.08	377.95
	691.08	694.07	697.57	701.10
	691.09	694.07	697.57	701.10
	751.47	752.66	753.84	755.01
	2203.29	2209.42	2215.50	2221.52
	3336.65	3343.93	3351.19	3358.40
HCCF	423.22	426.17	429.05	431.92
	423.22	426.17	429.05	431.92
	611.78	615.87	620.67	625.42
	611.78	615.87	620.67	625.42
	1079.55	1081.92	1084.27	1086.61
	2324.74	2330.83	2336.87	2342.88
	3349.48	3356.71	3363.93	3371.12
HCCH	761.69	763.51	765.52	767.52
	761.69	763.51	765.52	767.52

	821.80	824.59	827.42	830.22
	821.80	824.59	827.42	830.22
	2071.53	2076.77	2081.96	2087.11
	3287.99	3295.06	3302.12	3309.14
	3439.38	3446.65	3453.89	3461.09
trans-CHClCHCl	353.70	354.52	355.34	356.14
	827.73	831.51	811.28	813.04
	807.69	809.50	835.25	838.94
	854.54	856.81	859.05	861.27
	957.39	961.50	965.56	969.59
	1243.74	1247.95	1252.15	1256.32
	1322.31	1326.71	1331.08	1335.43
	1654.87	1660.28	1665.60	1670.86
	3104.32	3112.02	3119.70	3127.34
	3158.13	3165.84	3173.53	3181.19
cis-CHClCHCl	425.71	427.01	428.28	429.52
	574.47	575.74	577.00	578.25
	710.19	711.60	713.00	714.38
	756.04	759.01	761.95	764.87
	851.22	853.40	855.56	857.69
	952.63	957.29	961.90	966.47
	1237.92	1242.05	1246.16	1250.24
	1334.57	1339.18	1343.78	1348.36
	1655.88	1661.25	1666.56	1671.80
	3077.57	3085.13	3092.66	3100.16
	3150.68	3158.27	3165.82	3173.35
CH_2CCl_2	304.80	305.44	306.07	306.70
	397.56	398.51	399.45	400.37
	486.10	487.33	488.54	489.72
	602.58	603.97	605.34	606.69
	734.32	736.93	739.51	742.06
	782.80	785.77	788.70	791.60
	967.24	971.83	976.39	980.92
	1117.85	1121.74	1125.61	1129.47
	1421.55	1426.29	1431.01	1435.71
	1670.39	1675.72	1680.99	1686.21
	3095.09	3102.14	3109.16	3116.15
	3124.97	3132.09	3139.17	3146.22
cis-CHFCHF	518.77	520.43	522.07	523.68
	779.28	781.31	783.34	785.35
	821.96	825.31	828.64	831.94
	922.44	927.04	931.60	936.11
	1025.98	1028.62	1031.24	1033.83
	1129.23	1132.09	1134.94	1137.76
	1299.17	1303.22	1307.25	1311.26
	1400.35	1405.03	1409.69	1414.33
	1768.12	1773.85	1779.53	1785.16
	3101.69	3109.74	3117.74	3125.70
	3176.08	3184.14	3192.15	3200.11
trans-CHFCHF	329.25	330.34	331.42	332.49
	338.87	340.06	341.24	342.40
	559.31	560.79	562.25	563.71
	859.52	863.70	867.84	871.93
	934.55	938.70	942.82	946.91

	1142.58	1145.64	1148.67	1151.67
	1150.55	1153.33	1156.09	1158.82
	1307.21	1311.64	1316.04	1320.43
	1323.38	1327.67	1331.95	1336.20
	1756.02	1761.79	1767.49	1773.13
	3111.05	3119.07	3127.05	3134.99
	3168.17	3176.18	3184.16	3192.09
OCHCHO	354.59	355.91	357.24	358.56
	559.05	560.86	562.65	564.41
	825.71	829.28	832.78	836.26
	1060.92	1064.85	1068.71	1072.51
	1094.83	1099.41	1103.88	1108.26
	1351.33	1356.20	1360.99	1365.74
	1391.98	1396.82	1401.61	1406.33
	1815.85	1821.75	1827.53	1833.20
	1831.48	1838.51	1845.40	1852.16
	2834.11	2843.95	2853.72	2863.41
	2890.59	2900.17	2909.68	2919.11
CH_2CH_2	862.23	864.50	866.77	869.02
	998.92	1002.61	1006.29	1009.96
	1002.07	1006.10	1010.10	1014.09
	1069.38	1072.32	1075.24	1078.14
	1256.75	1260.56	1264.36	1268.16
	1392.36	1396.02	1399.67	1403.29
	1486.21	1490.85	1495.49	1500.13
	1694.73	1699.44	1704.12	1708.78
	3064.53	3071.49	3078.42	3085.32
	3104.27	3111.37	3118.45	3125.49
	3122.61	3129.66	3136.67	3143.66
	3152.37	3159.40	3166.40	3173.37
cyclo-C_2H_4O	845.50	847.88	850.24	852.58
	843.35	846.34	849.29	852.19
	890.93	893.30	895.65	897.97
	1064.45	1068.09	1071.71	1075.32
	1176.78	1181.39	1185.98	1190.56
	1188.31	1192.90	1197.47	1202.03
	1187.42	1191.26	1195.08	1198.89
	1194.79	1198.55	1202.31	1206.05
	1313.53	1317.18	1320.81	1324.43
	1517.21	1522.12	1527.02	1531.91
	1553.79	1558.98	1564.16	1569.33
	3024.93	3032.43	3039.89	3047.30
	3055.83	3063.42	3070.97	3078.48
	3092.97	3100.63	3108.23	3115.78
	3110.58	3118.27	3125.90	3133.48
cyclo-C_2H_4NH	814.78	817.22	819.64	822.03
	856.12	858.79	861.43	864.03
	880.95	883.23	885.50	887.74
	953.54	956.45	959.33	962.21
	1033.67	1036.42	1039.16	1041.88
	1139.60	1143.71	1147.82	1151.92
	1146.94	1151.33	1155.70	1160.06
	1168.47	1172.30	1176.12	1179.94
	1256.39	1260.18	1263.93	1267.66

	1283.26	1287.27	1291.27	1295.26
	1302.10	1305.49	1308.88	1312.27
	1511.26	1516.11	1520.94	1525.77
	1541.37	1546.46	1551.53	1556.59
	3031.65	3038.68	3045.68	3052.64
	3062.71	3069.85	3076.95	3084.02
	3097.20	3104.34	3111.44	3118.50
	3115.57	3122.77	3129.94	3137.05
	3356.52	3366.69	3376.78	3386.78
CH_2CCHCl	366.78	368.07	369.35	370.62
	512.76	514.33	515.86	517.37
	618.12	619.88	621.61	623.31
	759.85	762.28	764.68	767.03
	888.21	891.61	894.96	898.27
	965.86	969.98	974.08	978.16
	1042.08	1045.55	1049.00	1052.44
	1144.24	1147.15	1150.02	1152.88
	1289.32	1293.50	1297.66	1301.80
	1483.52	1488.33	1493.12	1497.90
	2049.87	2054.99	2060.06	2065.06
	3044.96	3052.42	3059.84	3067.22
	3052.22	3060.07	3067.87	3075.62
	3075.52	3083.27	3090.97	3098.65
CH_2CHCHO	349.79	350.64	351.48	352.33
	582.42	583.65	584.88	586.09
	643.51	645.27	647.01	648.74
	945.69	948.01	950.32	952.62
	1034.65	1038.77	1042.85	1046.89
	1051.52	1055.11	1058.56	1061.91
	1054.67	1057.39	1060.03	1062.62
	1181.84	1185.13	1188.41	1191.66
	1316.72	1320.76	1324.79	1328.79
	1403.11	1407.77	1412.39	1416.96
	1475.10	1479.84	1484.56	1489.25
	1686.05	1691.21	1696.29	1701.30
	1788.64	1795.11	1801.46	1807.71
	2771.05	2780.95	2790.76	2800.50
	3058.41	3065.77	3073.09	3080.37
	3054.63	3062.19	3069.72	3077.21
	3099.08	3106.24	3113.38	3120.49
cyclo-C_3H_6	791.64	793.71	795.76	797.80
	787.55	789.68	791.81	793.92
	897.96	900.13	902.30	904.45
	875.47	877.23	878.98	880.72
	875.40	877.17	878.92	880.65
	1089.70	1093.65	1097.60	1101.53
	1084.92	1088.95	1092.97	1096.98
	1123.70	1127.88	1132.05	1136.21
	1169.43	1173.11	1176.79	1180.47
	1218.93	1221.25	1223.57	1225.86
	1230.63	1234.38	1238.12	1241.86
	1230.48	1234.24	1238.00	1241.74
	1490.55	1495.27	1499.98	1504.69
	1489.38	1494.13	1498.87	1503.61

	1538.85	1543.86	1548.86	1553.85
	3053.04	3059.64	3066.22	3072.76
	3061.19	3067.73	3074.24	3080.71
	3093.25	3099.96	3106.63	3113.28
	3114.31	3120.96	3127.56	3134.13
	3114.67	3121.29	3127.88	3134.43
	3156.68	3163.35	3169.99	3176.58
$CH_2CHCHCH_2$	537.76	538.84	539.91	540.97
	604.08	605.40	606.72	608.00
	816.90	819.26	821.58	823.86
	922.89	924.61	926.33	928.04
	992.52	996.52	1000.48	1004.40
	995.67	999.74	1003.77	1007.77
	1042.31	1045.34	1048.35	1051.35
	1033.13	1037.03	1040.90	1044.73
	1082.26	1085.74	1089.19	1092.61
	1238.42	1241.55	1244.68	1247.81
	1333.94	1337.98	1341.99	1345.99
	1339.81	1343.99	1348.16	1352.31
	1435.45	1439.76	1444.04	1448.29
	1494.59	1499.26	1503.92	1508.57
	1659.76	1664.69	1669.56	1674.38
	1712.96	1718.71	1724.37	1729.96
	3064.11	3072.22	3080.29	3088.30
	3068.57	3075.21	3081.88	3088.58
	3097.10	3104.24	3111.22	3118.06
	3034.05	3040.63	3047.32	3054.09
	3139.58	3146.52	3153.42	3160.29
	3136.68	3143.75	3150.78	3157.78
$^1CH_2$	1366.65	1374.03	1380.70	1386.83
	2890.66	2899.13	2907.65	2916.19
	2894.93	2902.35	2909.99	2917.79
$^3CH_2$	990.07	996.18	1002.25	1008.28
	3097.97	3104.74	3111.47	3118.18
	3281.43	3287.86	3294.26	3300.63
HCO	1094.41	1098.34	1102.18	1105.94
	1956.48	1962.33	1968.10	1973.79
	2478.12	2487.07	2496.02	2504.95

Table S12. VPT2 nMR (n = 2-3) vibrational frequencies in cm^{-1} for the modified F1 anharmonic test set calculated at the KS-DFT and TAO-DFT B97-1/aug-cc-pVTZ levels of electronic structure.

	KS-DFT		TAO-DFT	
Molecule	2MR	3MR	2MR	3MR
$NClF_2$	356.84	356.63	356.92	356.72
	368.53	368.49	367.92	367.88
	560.82	560.68	561.36	561.23
	692.98	692.88	690.38	690.28
	847.36	846.40	851.14	850.14
	949.40	948.93	951.75	951.29
ClF_3	409.04	408.98	407.09	407.03
	524.68	524.65	526.23	526.21
	693.39	693.06	694.09	693.74
	743.98	743.87	730.62	730.53
HOCl	746.16	744.34	741.22	739.37
	1233.22	1231.07	1231.63	1229.45
	3603.53	3605.29	3615.55	3617.32
ClNO	342.58	342.02	323.70	323.21
	615.14	614.48	592.06	591.47
	1865.17	1865.67	1866.57	1866.99
$ClNO_2$	383.38	383.27	375.93	375.82
	409.73	409.33	407.53	407.13
	666.06	666.06	655.76	655.76
	803.34	802.67	799.05	798.37
	1224.50	1225.17	1389.26	1389.93
	1709.74	1717.62	1711.22	1712.31
ClSN	417.38	417.38	403.41	403.41
	1348.00	1348.00	1338.50	1338.50
NCl_2F	340.10	339.41	338.48	337.71
	427.58	427.36	424.48	424.24
	623.62	622.66	619.23	618.15
	678.89	676.49	673.05	670.41
	848.84	848.82	849.72	849.70
Cl_2O	666.71	666.71	638.43	638.43
	673.96	673.96	659.94	659.94
$SOCl_2$	178.59	178.59	177.38	177.38
	276.37	276.37	275.42	275.42
	334.43	334.16	332.16	331.90
	447.51	447.51	442.12	442.12
SCl_2	486.62	486.31	482.56	482.25
	1245.28	1245.69	1246.44	1246.84
HOF	507.18	507.18	484.88	484.88
	518.37	518.37	511.35	511.35
	987.70	986.08	986.69	985.05
ONF	1381.60	1379.74	1383.52	1381.64
	3558.41	3560.80	3573.29	3575.69
	531.42	531.01	531.70	531.29
NSF	785.59	785.11	784.59	784.11
	1894.43	1895.16	1894.18	1894.91
	364.37	364.19	364.58	364.41
F_2NH	625.46	625.23	628.24	628.02
	1383.02	1383.39	1377.70	1378.07

	498.86	498.82	500.22	500.18
	881.29	879.12	884.64	882.46
	987.29	984.65	989.01	986.35
	1301.42	1297.02	1303.97	1299.53
N_2F_2	1423.58	1420.74	1427.17	1424.32
	3175.55	3182.70	3190.77	3197.97
	364.61	364.61	364.34	364.34
	416.84	416.41	417.63	417.20
	607.52	607.52	608.90	608.90
	999.13	998.11	1003.65	1002.62
F_2O	1036.38	1035.51	1039.69	1038.81
	1567.93	1568.98	1569.12	1570.22
	485.42	485.42	484.18	484.18
F_2SO	901.74	901.74	894.59	894.59
	1024.17	1024.17	1023.85	1023.85
	348.68	348.65	350.02	349.99
	375.03	374.67	375.84	375.48
	502.35	502.18	504.07	503.91
	704.71	703.69	708.25	707.21
S_2F_2	770.64	770.16	774.41	773.93
	1316.63	1317.95	1320.73	1322.07
	588.97	588.90	591.42	591.35
HNO_3	663.08	662.95	661.54	661.41
	698.32	698.30	698.97	698.94
	431.81	430.13	429.42	427.72
	584.24	577.52	584.88	578.11
	646.66	645.27	649.57	648.21
	773.55	771.23	771.92	769.58
	892.31	890.48	895.74	893.88
	1297.14	1305.50	1297.85	1305.51
	1328.50	1322.62	1329.54	1323.63
HN_3	1713.72	1709.49	1716.71	1712.54
	3545.14	3564.00	3557.08	3575.99
	540.62	532.20	538.35	529.89
	599.66	599.66	599.64	599.64
	1160.51	1148.57	1163.22	1151.19
	1283.49	1275.06	1285.82	1277.42
H_2O	2190.41	2185.05	2195.31	2189.97
	3298.70	3313.21	3310.53	3324.98
	1568.49	1568.49	1573.01	1573.01
H_2O_2	3654.95	3654.95	3670.20	3670.20
	3730.35	3730.35	3745.45	3745.45
	274.13	273.64	273.19	272.68
	951.48	947.45	953.53	949.43
	1289.65	1275.45	1291.93	1277.62
	1411.06	1397.51	1414.43	1400.77
H_2S	3528.54	3555.24	3542.49	3569.25
	3601.79	3603.95	3616.18	3618.37
	1175.06	1175.06	1178.92	1178.92
H_2S_2	2572.33	2572.33	2585.00	2585.00
	2577.30	2577.30	2588.65	2588.65
	389.97	389.78	378.21	378.00
	502.39	501.44	498.03	497.02
	889.68	880.15	886.96	877.26

	899.97	890.35	894.95	885.10
NO_2	2511.21	2511.20	2522.69	2522.70
	2484.11	2497.88	2495.32	2509.06
	755.96	755.96	747.06	747.06
N_2O	1362.51	1362.51	1359.75	1359.75
	1638.00	1638.00	1638.08	1638.08
	605.23	605.23	605.52	605.52
	605.23	605.23	605.52	605.52
SO_2	1314.45	1314.45	1317.87	1317.87
	2270.20	2270.20	2277.34	2277.34
	513.38	513.38	512.34	512.34
O_3	1160.85	1160.85	1158.55	1158.55
	1342.40	1342.40	1342.48	1342.48
	740.94	740.94	724.08	724.08
COClF	1209.60	1209.60	1192.78	1192.78
	1255.23	1255.23	1231.02	1231.02
	402.24	401.81	403.27	402.84
	488.54	488.05	490.73	490.23
	664.11	664.11	663.89	663.89
	752.88	751.76	755.65	754.52
ClCN	1072.11	1069.32	1076.90	1074.08
	1880.23	1884.74	1883.76	1888.46
	391.66	391.66	391.83	391.83
	391.66	391.66	391.83	391.83
$COCl_2$	714.18	714.18	716.31	716.31
	2269.73	2269.73	2275.47	2275.47
	434.47	433.36	435.74	434.62
	559.36	558.35	561.75	560.75
	577.36	577.36	575.17	575.17
FCN	816.32	812.72	820.41	816.80
	1860.83	1861.34	1861.93	1862.45
	475.68	475.68	476.37	476.37
	475.68	475.68	476.37	476.37
COF_2	1103.18	1103.18	1106.29	1106.29
	2365.71	2365.71	2373.07	2373.07
	574.81	574.74	576.05	575.98
	613.14	612.33	614.49	613.68
	770.84	770.84	771.91	771.91
	959.06	957.82	962.57	961.34
CSF_2	1211.65	1209.09	1217.08	1214.54
	1940.77	1941.65	1947.14	1948.04
	415.55	415.33	415.01	414.80
	522.84	522.64	522.85	522.65
	626.03	626.03	618.29	618.29
	789.43	788.83	789.25	788.66
COS	1178.38	1152.22	1183.58	1100.78
	1339.97	1345.59	1341.97	1347.28
	520.95	520.95	520.00	520.00
	520.95	520.95	520.00	520.00
CO_2	853.00	853.00	856.04	856.04
	2086.86	2086.86	2092.20	2092.20
	667.71	667.71	668.83	668.83
	667.71	667.71	668.83	668.83
CS_2	1472.79	1472.79	1471.78	1471.78

	2362.37	2362.37	2370.25	2370.25
	399.82	399.82	395.43	395.43
	399.82	399.82	395.43	395.43
HCN	655.34	655.34	655.16	655.16
	1522.36	1522.36	1522.59	1522.59
	732.64	732.64	735.02	735.02
	732.64	732.64	735.03	735.02
HNCO	2163.90	2163.90	2170.16	2170.16
	3301.54	3301.54	3316.37	3316.37
	592.75	552.23	593.35	552.40
	624.83	624.83	626.03	626.03
	809.30	764.80	809.64	764.70
	1335.69	1325.89	1338.96	1328.69
H_2CO	2275.50	2281.80	2283.29	2289.42
	3489.19	3513.72	3502.16	3526.63
	1166.11	1166.11	1164.90	1164.90
	1239.89	1236.60	1242.15	1238.78
	1496.24	1493.47	1499.87	1497.02
	1796.05	1794.53	1797.44	1795.91
HCOOH	2727.85	2726.96	2745.38	2744.44
	2745.75	2761.58	2765.32	2777.51
	433.19	432.15	433.58	432.54
	660.62	650.38	661.97	651.64
	1008.58	1006.94	1010.70	1009.05
	1101.22	1092.54	1105.29	1096.31
	1237.95	1221.39	1240.98	1224.25
	1379.94	1377.60	1384.58	1382.24
	1830.14	1829.53	1833.96	1833.35
C_2Cl_2	2822.15	2818.84	2838.59	2834.98
	3611.37	3626.75	3624.03	3639.66
	345.28	345.28	344.14	344.14
	345.28	345.28	344.14	344.14
	466.79	466.79	467.75	467.75
C_2N_2	980.58	980.58	982.81	982.81
	2277.25	2277.25	2281.30	2281.30
	539.16	539.16	539.90	539.91
	539.16	539.16	539.90	539.91
	859.44	859.44	862.78	862.78
HCCCl	2228.19	2228.19	2232.35	2232.35
	2389.14	2389.14	2391.29	2391.29
	347.45	338.80	347.06	338.22
	347.45	338.80	347.06	338.22
	619.84	610.33	621.63	611.91
	619.84	610.33	621.64	611.92
	757.06	756.98	758.89	758.80
HCCF	2151.11	2151.07	2156.48	2156.44
	3338.73	3345.04	3352.65	3359.08
	393.64	391.66	393.97	392.22
	393.64	391.66	393.97	392.22
	580.58	578.46	582.42	580.55
	580.58	578.46	582.42	580.55
	1081.80	1081.76	1084.77	1084.72
HCCH	2272.95	2272.84	2280.18	2280.07
	3354.38	3358.92	3368.68	3372.92

	692.51	639.78	694.54	641.63
	692.51	639.79	694.54	641.63
	803.54	748.72	805.58	750.58
	803.54	748.72	805.58	750.59
	2025.72	2025.72	2031.77	2031.77
trans-CHClCHCl	3214.44	3277.61	3228.39	3291.73
	3373.90	3373.90	3388.35	3388.35
	346.87	346.20	347.65	346.98
	801.31	776.45	800.92	775.78
	802.26	801.68	807.03	806.46
	834.47	833.04	837.74	836.29
	929.99	903.81	930.39	903.91
	1201.09	1192.45	1203.84	1195.22
	1280.01	1271.52	1282.45	1273.93
	1641.52	1636.60	1552.30	1547.86
cis-CHClCHCl	3033.60	3082.18	3047.15	3095.76
	3057.68	3064.46	3065.65	3072.32
	416.79	410.80	414.69	408.56
	564.60	563.24	566.06	564.71
	735.26	696.69	735.67	696.61
	703.03	700.57	707.08	704.60
	837.49	833.51	841.00	836.98
	916.26	875.65	917.04	875.94
	1192.71	1183.74	1193.99	1184.92
	1292.45	1284.62	1295.63	1287.97
	1593.11	1590.14	1595.16	1592.16
CH_2CCl_2	3006.51	3066.56	3021.08	3081.04
	3060.97	3068.98	3072.12	3080.11
	293.61	293.61	294.02	294.02
	383.88	370.88	383.90	370.72
	469.48	463.35	467.73	461.54
	593.21	592.42	595.53	594.74
	718.85	678.07	718.69	677.40
	771.72	758.27	775.19	761.50
	919.14	875.46	920.51	876.28
	1089.67	1069.48	1092.93	1072.40
	1419.30	1412.03	1416.08	1407.92
	1627.62	1623.03	1629.46	1624.79
cis-CHFCHF	3013.64	3034.63	3026.84	3047.36
	3059.69	3117.33	3074.52	3132.39
	511.54	505.91	511.11	505.46
	798.73	763.83	800.52	765.36
	769.93	768.78	771.49	770.34
	881.94	845.76	884.41	847.96
	989.66	984.40	955.87	950.56
	1124.15	1118.81	1128.23	1122.84
	1266.63	1260.78	1269.84	1263.93
	1370.70	1360.93	1374.55	1364.68
	1724.76	1720.01	1730.31	1725.52
trans-CHFCHF	3023.11	3111.20	3038.06	3122.95
	3082.87	3094.90	3097.55	3109.53
	321.88	317.30	321.72	317.08
	333.88	333.41	333.92	333.45
	551.61	550.83	552.77	551.99

	824.22	800.33	826.08	802.01
	907.65	883.87	910.17	886.11
	1136.08	1134.78	1139.91	1138.59
	1153.93	1151.69	1158.68	1156.40
	1276.23	1266.09	1279.73	1269.48
	1289.96	1284.24	1293.07	1287.30
	1701.70	1698.64	1706.28	1703.19
OCHCHO	3027.91	3082.27	3043.08	3097.17
	3066.18	3076.34	3081.37	3091.38
	337.65	332.91	338.96	334.16
	538.38	538.06	540.28	539.97
	795.15	783.10	795.83	783.36
	1023.91	1021.67	1024.31	1022.06
	1065.79	1051.79	1058.66	1044.29
	1306.22	1295.77	1305.38	1294.83
	1347.81	1340.82	1346.93	1339.81
	1780.66	1781.23	1777.63	1778.26
	1780.64	1779.66	1775.77	1774.78
CH_2CH_2	2740.28	2775.16	2757.23	2790.61
	2786.44	2783.77	2802.58	2799.03
	843.68	825.52	844.96	826.59
	972.85	952.38	975.10	954.44
	972.54	952.45	974.80	954.53
	1053.43	1035.06	1054.84	1036.31
	1232.00	1219.01	1235.43	1222.29
	1355.47	1350.13	1359.53	1354.12
	1449.18	1433.43	1453.81	1437.89
	1655.45	1648.30	1660.19	1652.97
	3002.19	2922.64	3017.20	2928.32
	2996.72	2999.10	3011.63	3013.90
cyclo-C_2H_4O	3059.20	3045.88	3075.56	3062.30
	3089.55	3070.24	3105.36	3086.23
	828.41	805.93	830.85	808.18
	816.11	811.36	820.70	815.95
	856.89	855.11	861.65	859.84
	1041.02	1021.93	1044.70	1025.46
	1140.54	1120.75	1144.07	1124.09
	1158.46	1137.28	1161.91	1140.54
	1158.69	1144.83	1162.46	1148.47
	1164.38	1149.61	1167.83	1152.90
	1270.02	1265.72	1275.10	1270.80
	1475.98	1462.55	1480.91	1467.36
	1497.20	1485.80	1502.68	1491.17
	2957.96	2987.58	2973.57	2998.96
	3029.45	3031.93	3030.91	3033.39
cyclo-C_2H_4NH	3023.58	3013.23	3040.52	3030.39
	3041.26	3028.27	3057.91	3045.17
	789.35	758.11	792.92	761.44
	818.53	812.78	823.98	818.19
	843.91	830.50	848.03	834.71
	929.87	902.39	932.51	904.86
	1012.70	984.27	1015.50	986.89
	1100.85	1078.36	1104.56	1081.86
	1112.07	1087.52	1115.57	1090.81

	1139.47	1117.29	1143.46	1121.10
	1212.68	1198.38	1217.79	1203.29
	1252.90	1233.03	1256.19	1236.20
	1273.14	1255.44	1276.89	1259.35
	1467.15	1451.55	1471.92	1456.21
	1483.72	1464.69	1488.73	1469.82
	2972.59	2983.32	2987.73	2997.64
	2982.84	2992.61	2996.85	3006.55
	3035.62	3028.06	3052.04	3044.66
CH_2CCHCl	3055.48	3041.79	3071.59	3058.13
	3300.40	3339.99	3313.90	3353.64
	362.99	318.94	363.90	319.20
	501.77	497.87	501.72	497.77
	609.91	556.37	607.73	553.51
	748.86	748.36	750.79	750.29
	864.54	822.35	862.77	820.71
	934.90	889.51	935.61	889.25
	1016.88	993.70	1018.17	994.63
	1121.46	1116.34	1120.43	1115.19
	1252.34	1246.79	1255.02	1249.35
	1441.66	1429.94	1445.48	1433.09
	2018.91	2024.82	2018.94	2026.25
	2971.68	2985.32	2985.73	2999.32
CH_2CHCHO	2983.70	3044.76	2998.97	3060.04
	3037.63	3055.94	3051.55	3069.39
	339.91	323.85	340.33	324.08
	568.08	564.36	569.13	565.31
	631.14	592.06	632.07	592.34
	918.27	898.23	921.59	901.31
	1004.34	974.80	1003.66	973.51
	1020.96	1000.25	1020.56	1000.05
	1033.68	1008.65	1033.91	1007.81
	1148.20	1138.91	1150.73	1141.43
	1278.55	1266.56	1281.57	1269.42
	1363.85	1356.51	1365.60	1358.36
	1432.66	1411.67	1435.62	1414.44
	1638.45	1630.45	1639.26	1631.08
	1746.14	1744.74	1743.73	1742.48
	2705.20	2750.52	2717.81	2753.87
	2971.87	3313.62	2985.91	3078.36
cyclo-C_3H_6	2993.24	3035.71	3008.17	3047.17
	3031.01	3077.30	3046.55	3094.75
	766.09	729.40	768.43	731.47
	760.93	724.25	763.51	726.55
	873.32	844.21	875.22	845.85
	833.96	830.57	839.41	836.01
	833.37	830.57	838.83	836.01
	1050.66	1017.64	1053.97	1020.74
	1044.15	1010.87	1047.67	1014.18
	1082.60	1050.08	1086.20	1053.44
	1138.06	1110.49	1142.31	1114.54
	1176.55	1175.25	1182.07	1180.73
	1195.97	1176.98	1199.69	1180.55
	1196.36	1177.41	1200.11	1181.01

	1448.40	1421.33	1453.21	1424.98
	1434.98	1414.57	1437.29	1416.57
	1500.21	1483.99	1505.21	1488.86
	2989.52	2999.40	3005.19	3014.46
	2995.42	2999.95	3010.21	3015.18
	3009.24	3009.30	3024.06	3024.12
	3055.88	3049.47	3072.32	3066.06
$CH_2CHCHCH_2$	3056.62	3049.97	3073.06	3066.69
	3099.35	3072.01	3115.13	3088.09
	522.71	513.97	523.33	514.43
	600.04	534.54	601.23	534.71
	806.82	770.04	806.02	768.58
	898.36	883.26	902.23	886.95
	967.89	925.18	968.62	925.22
	968.12	923.57	968.83	923.55
	1008.98	978.77	1009.21	978.82
	1006.86	987.85	1008.73	989.50
	1058.45	1028.87	1059.88	1029.87
	1206.91	1198.99	1210.67	1202.73
	1296.66	1287.44	1299.04	1289.70
	1300.47	1316.24	1303.34	1302.92
	1394.09	1380.34	1397.41	1383.49
	1447.32	1433.92	1451.41	1439.94
	1615.87	1604.71	1618.37	1606.88
	1656.92	1646.84	1655.19	1644.92
	2975.56	2974.47	2991.79	2981.62
	3014.42	3100.48	3029.61	3076.43
	2988.69	2993.04	3002.76	3005.21
	2983.81	3002.24	3001.27	3014.22
$^1CH_2$	3077.12	3076.09	3092.83	3092.03
	3075.88	3076.29	3091.40	3092.21
	1354.91	1354.91	1285.31	1285.31
$^3CH_2$	2781.66	2781.66	2799.45	2799.45
	2811.12	2811.12	2856.27	2856.27
	956.17	956.17	960.77	960.77
HCO	2995.22	2995.22	3010.87	3010.87
	3205.66	3205.66	3221.85	3221.85
	1064.58	1064.06	1057.89	1057.35

Table S13. Q-Chem print out of the harmonic force field for the KS-DFT B97-1/aug-cc-pVTZ calculation of HCCH.

```
****************************************************************************
**                                                                        **
**                  VIBRATIONAL ANALYSIS                                  **
**                  --------------------                                  **
**                                                                        **
**        VIBRATIONAL FREQUENCIES (CM**-1) AND NORMAL MODES               **
**    FORCE CONSTANTS (mDYN/ANGSTROM) AND REDUCED MASSES (AMU)            **
**              INFRARED INTENSITIES (KM/MOL)                             **
**                                                                        **
****************************************************************************
Mode:              1                  2                  3
 Frequency:      649.88             649.89             763.22
 Force Cnst:     0.3916             0.3916             0.3723
 Red. Mass:      1.5735             1.5735             1.0848
 IR Active:       YES                YES                YES
 IR Intens:      0.000              0.000             100.669
 Raman Active:     YES                YES                YES
              X      Y      Z       X      Y      Z       X      Y      Z
 C          0.020  0.159  0.000   -0.159  0.020 -0.000   -0.000 -0.059  0.000
 C         -0.020 -0.159 -0.000    0.159 -0.020  0.000   -0.000 -0.059  0.000
 H          0.088  0.683 -0.000   -0.683  0.088  0.000    0.002  0.705  0.000
 H         -0.088 -0.683  0.000    0.683 -0.088 -0.000    0.002  0.705 -0.000
 TransDip  -0.000  0.000 -0.000    0.000 -0.000  0.000    0.001  0.321 -0.000

 Mode:              4                  5                  6
 Frequency:      763.22             2058.58            3404.01
 Force Cnst:     0.3723             9.4774             7.4061
 Red. Mass:      1.0848             3.7958             1.0848
 IR Active:       YES                YES                YES
 IR Intens:     100.669             0.000              94.143
 Raman Active:     YES                YES                YES
              X      Y      Z       X      Y      Z       X      Y      Z
 C         -0.059  0.000  0.000   -0.000 -0.000  0.356    0.000 -0.000 -0.059
 C         -0.059  0.000 -0.000    0.000  0.000 -0.356   -0.000 -0.000 -0.059
 H          0.705 -0.002 -0.000   -0.000  0.000 -0.611   -0.000  0.000  0.705
 H          0.705 -0.002  0.000   -0.000  0.000  0.611   -0.000  0.000  0.705
 TransDip   0.321 -0.001  0.000   -0.000  0.000  0.000   -0.000  0.000  0.311

 Mode:              7
 Frequency:     3509.74
 Force Cnst:     8.9368
 Red. Mass:      1.2314
 IR Active:       YES
 IR Intens:      0.000
 Raman Active:     YES
              X      Y      Z
 C          0.000  0.000  0.101
 C          0.000  0.000 -0.101
 H         -0.000 -0.000  0.700
 H         -0.000 -0.000 -0.700
 TransDip  -0.000 -0.000  0.000
```

Table S14. Q-Chem print out of the anharmonic force field for the KS-DFT B97-1/aug-cc-pVTZ calculation of HCCH.

```
===========================================================================
     ANHARMONIC CUBIC & QUARTIC FORCE FIELD (CM**-1)
___________________________________________________________________
Eta[1,1,1]=-0.0000346652,
 Eta[1,1,1,1]=856.3214686977,
 Eta[1,1,2]=0.0000248329,
 Eta[1,1,1,2]=0.8610824031,
 Eta[1,1,2,2]=287.7601576191,
 Eta[1,1,3]=0.0001831709,
 Eta[1,1,1,3]=-0.0001022829,
 Eta[1,1,3,3]=778.2634449489,
 Eta[1,1,4]=0.0001321071,
 Eta[1,1,1,4]=-0.0000204325,
 Eta[1,1,4,4]=260.5255096159,
 Eta[1,1,5]=-121.0508591008,
 Eta[1,1,1,5]=-0.0000559080,
 Eta[1,1,5,5]=-52.0787064335,
 Eta[1,1,6]=-0.0001291545,
 Eta[1,1,1,6]=0.0000257811,
 Eta[1,1,6,6]=-541.6396452300,
 Eta[1,1,7]=934.3941921540,
 Eta[1,1,1,7]=0.0000257777,
 Eta[1,1,7,7]=-533.8870339761,
 Eta[2,2,2]=0.0000726078,
 Eta[2,2,2,2]=856.3194454640,
 Eta[2,2,1]=-0.0000078154,
 Eta[2,2,2,1]=-0.8610759758,
 Eta[2,2,3]=0.0002322030,
 Eta[2,2,2,3]=-0.0007417003,
 Eta[2,2,3,3]=260.5257887075,
 Eta[2,2,4]=-0.0000012608,
 Eta[2,2,2,4]=0.0059696541,
 Eta[2,2,4,4]=778.2609459968,
 Eta[2,2,5]=-121.0507175314,
 Eta[2,2,2,5]=0.0002236707,
 Eta[2,2,5,5]=-52.0786445825,
 Eta[2,2,6]=-0.0073147681,
 Eta[2,2,2,6]=-0.0001304986,
 Eta[2,2,6,6]=-541.6390097108,
 Eta[2,2,7]=934.3930877724,
 Eta[2,2,2,7]=-0.0000721775,
 Eta[2,2,7,7]=-533.8863968381,
 Eta[3,3,3]=0.0006393452,
 Eta[3,3,3,3]=784.1243054404,
 Eta[3,3,1]=-0.0002526112,
 Eta[3,3,3,1]=-0.0000089070,
 Eta[3,3,2]=0.0000144794,
 Eta[3,3,3,2]=-0.0000250880,
 Eta[3,3,4]=0.0001002707,
 Eta[3,3,3,4]=-0.0119264401,
 Eta[3,3,4,4]=265.5121111458,
```

Eta[3,3,5]=-149.6436557959,
Eta[3,3,3,5]=0.0001167510,
Eta[3,3,5,5]=-22.7542887858,
Eta[3,3,6]=-0.0000006555,
Eta[3,3,3,6]=0.0002408438,
Eta[3,3,6,6]=-548.3615853840,
Eta[3,3,7]=914.4291581884,
Eta[3,3,3,7]=-0.0004113903,
Eta[3,3,7,7]=-516.7861992419,
Eta[4,4,4]=0.0003086843,
Eta[4,4,4,4]=784.1211165600,
Eta[4,4,1]=0.0000023354,
Eta[4,4,4,1]=0.0005638111,
Eta[4,4,2]=-0.0000824390,
Eta[4,4,4,2]=-0.0046638345,
Eta[4,4,3]=0.0002089331,
Eta[4,4,4,3]=0.0119231181,
Eta[4,4,5]=-149.6433715484,
Eta[4,4,4,5]=-0.0002193774,
Eta[4,4,5,5]=-22.7542586330,
Eta[4,4,6]=0.0063346716,
Eta[4,4,4,6]=-0.0001122581,
Eta[4,4,6,6]=-548.3605751112,
Eta[4,4,7]=914.4274356447,
Eta[4,4,4,7]=-0.0001607429,
Eta[4,4,7,7]=-516.7852629380,
Eta[5,5,5]=528.3401372825,
Eta[5,5,5,5]=116.4061294224,
Eta[5,5,1]=0.0000848686,
Eta[5,5,5,1]=0.0000230356,
Eta[5,5,2]=-0.0003459904,
Eta[5,5,5,2]=-0.0000972728,
Eta[5,5,3]=-0.0000361638,
Eta[5,5,5,3]=-0.0000050302,
Eta[5,5,4]=0.0005693981,
Eta[5,5,5,4]=0.0001306042,
Eta[5,5,6]=-0.0000009549,
Eta[5,5,5,6]=-0.0000002479,
Eta[5,5,6,6]=26.0425836310,
Eta[5,5,7]=123.8527847749,
Eta[5,5,5,7]=39.9253036012,
Eta[5,5,7,7]=41.5263083409,
Eta[6,6,6]=0.0000242483,
Eta[6,6,6,6]=554.5919780014,
Eta[6,6,1]=0.0000902373,
Eta[6,6,6,1]=-0.0000150196,
Eta[6,6,2]=-0.0000768152,
Eta[6,6,6,2]=0.0000643654,
Eta[6,6,3]=-0.0003341049,
Eta[6,6,6,3]=-0.0001241666,
Eta[6,6,4]=-0.0000577855,
Eta[6,6,6,4]=0.0000652623,
Eta[6,6,5]=312.8584538571,
Eta[6,6,6,5]=0.0000013341,
Eta[6,6,7]=-1379.2974475418,

Eta[6,6,6,7]=-0.0000067005,
Eta[6,6,7,7]=509.3852670998,
Eta[7,7,7]=-1270.1961374237,
Eta[7,7,7,7]=474.9914736224,
Eta[7,7,1]=0.0000451440,
Eta[7,7,7,1]=-0.0000101577,
Eta[7,7,2]=-0.0001359688,
Eta[7,7,7,2]=0.0000218589,
Eta[7,7,3]=-0.0004090423,
Eta[7,7,7,3]=0.0001963227,
Eta[7,7,4]=-0.0001071044,
Eta[7,7,7,4]=0.0001203584,
Eta[7,7,5]=371.9466292118,
Eta[7,7,7,5]=-105.4604590003,
Eta[7,7,6]=-0.0000066867,
Eta[7,7,7,6]=0.0000045134,

Table S15. Q-Chem print out of the harmonic force field for the TAO-DFT B97-1/aug-cc-pVTZ calculation of HCCH.

```
**********************************************************************
**                                                                  **
**                   VIBRATIONAL ANALYSIS                           **
**                   --------------------                           **
**                                                                  **
**       VIBRATIONAL FREQUENCIES (CM**-1) AND NORMAL MODES          **
**    FORCE CONSTANTS (mDYN/ANGSTROM) AND REDUCED MASSES (AMU)      **
**                INFRARED INTENSITIES (KM/MOL)                     **
**                                                                  **
**********************************************************************
Mode:               1                2                3
Frequency:      652.70           652.70           765.67
Force Cnst:     0.3949           0.3949           0.3747
Red. Mass:      1.5733           1.5733           1.0848
IR Active:         YES              YES              YES
IR Intens:       0.000            0.000           98.095
Raman Active:      YES              YES              YES
              X      Y      Z      X      Y      Z      X      Y      Z
C         0.047  0.153  0.000  0.153 -0.047  0.000  0.002 -0.059  0.000
C        -0.047 -0.153 -0.000 -0.153  0.047  0.000  0.002 -0.059 -0.000
H         0.201  0.659 -0.000  0.659 -0.201 -0.000 -0.022  0.704  0.000
H        -0.201 -0.659  0.000 -0.659  0.201  0.000 -0.022  0.704 -0.000
TransDip  0.000  0.000  0.000  0.000 -0.000  0.000 -0.010  0.317  0.000

Mode:               4                5                6
Frequency:      765.67          2065.03          3418.26
Force Cnst:     0.3747           9.5511           7.4682
Red. Mass:      1.0848           3.8015           1.0848
IR Active:         YES              YES              YES
IR Intens:      98.095            0.000           94.453
Raman Active:      YES              YES              YES
              X      Y      Z      X      Y      Z      X      Y      Z
C        -0.059 -0.002  0.000  0.000  0.000  0.356  0.000 -0.000 -0.059
C        -0.059 -0.002 -0.000  0.000  0.000 -0.356 -0.000 -0.000 -0.059
H         0.704  0.022  0.000 -0.000 -0.000 -0.611 -0.000  0.000  0.705
H         0.704  0.022  0.000 -0.000 -0.000  0.611 -0.000  0.000  0.705
TransDip  0.317  0.010 -0.000 -0.000 -0.000  0.000 -0.000  0.000  0.311

Mode:               7
Frequency:     3524.30
Force Cnst:     9.0067
Red. Mass:      1.2308
IR Active:         YES
IR Intens:       0.000
Raman Active:      YES
              X      Y      Z
C         0.000  0.000  0.101
C        -0.000  0.000 -0.101
H        -0.000 -0.000  0.700
H        -0.000 -0.000 -0.700
TransDip -0.000 -0.000 -0.000
```

Table S16. Q-Chem print out of the anharmonic force field for the TAO-DFT B97-1/aug-cc-pVTZ calculation of HCCH.

===
ANHARMONIC CUBIC & QUARTIC FORCE FIELD (CM**-1)

Eta[1,1,1]=-0.0002242,
Eta[1,1,1,1]=854.5050404,
Eta[1,1,2]=-0.0006353,
Eta[1,1,1,2]=-1.5187548,
Eta[1,1,2,2]=287.1969611,
Eta[1,1,3]=0.0006748,
Eta[1,1,1,3]=0.0003114,
Eta[1,1,3,3]=733.0207129,
Eta[1,1,4]=0.0005509,
Eta[1,1,1,4]=0.0001316,
Eta[1,1,4,4]=308.1297644,
Eta[1,1,5]=-120.7458776,
Eta[1,1,1,5]=-0.0003121,
Eta[1,1,5,5]=-52.1882626,
Eta[1,1,6]=0.0001799,
Eta[1,1,1,6]=0.0005098,
Eta[1,1,6,6]=-542.5078758,
Eta[1,1,7]=937.8390970,
Eta[1,1,1,7]=0.0006288,
Eta[1,1,7,7]=-534.8176626,
Eta[2,2,2]=-0.0014617,
Eta[2,2,2,2]=854.5001375,
Eta[2,2,1]=-0.0002445,
Eta[2,2,2,1]=1.5178549,
Eta[2,2,3]=0.0011803,
Eta[2,2,2,3]=-0.0011217,
Eta[2,2,3,3]=308.1304671,
Eta[2,2,4]=-0.0001839,
Eta[2,2,2,4]=0.0034139,
Eta[2,2,4,4]=733.0202005,
Eta[2,2,5]=-120.7459012,
Eta[2,2,2,5]=-0.0001882,
Eta[2,2,5,5]=-52.1883384,
Eta[2,2,6]=0.0042608,
Eta[2,2,2,6]=0.0008275,
Eta[2,2,6,6]=-542.5085957,
Eta[2,2,7]=937.8384197,
Eta[2,2,2,7]=0.0008147,
Eta[2,2,7,7]=-534.8177803,
Eta[3,3,3]=0.0026223,
Eta[3,3,3,3]=786.3706561,
Eta[3,3,1]=-0.0012342,
Eta[3,3,3,1]=-0.0000361,
Eta[3,3,2]=-0.0005650,
Eta[3,3,3,2]=0.0000099,
Eta[3,3,4]=0.0005916,
Eta[3,3,3,4]=0.1276907,
Eta[3,3,4,4]=266.4474086,

Eta[3,3,5]=-149.2165985,
Eta[3,3,3,5]=0.0003697,
Eta[3,3,5,5]=-22.9419584,
Eta[3,3,6]=-0.0001323,
Eta[3,3,3,6]=0.0002795,
Eta[3,3,6,6]=-549.8851129,
Eta[3,3,7]=918.9734455,
Eta[3,3,3,7]=-0.0016751,
Eta[3,3,7,7]=-518.3045412,
Eta[4,4,4]=0.0017616,
Eta[4,4,4,4]=786.3704188,
Eta[4,4,1]=-0.0006486,
Eta[4,4,4,1]=-0.0009707,
Eta[4,4,2]=0.0003107,
Eta[4,4,4,2]=-0.0028589,
Eta[4,4,3]=0.0008821,
Eta[4,4,4,3]=-0.1277727,
Eta[4,4,5]=-149.2164109,
Eta[4,4,4,5]=0.0001548,
Eta[4,4,5,5]=-22.9422238,
Eta[4,4,6]=-0.0041817,
Eta[4,4,4,6]=-0.0010028,
Eta[4,4,6,6]=-549.8848877,
Eta[4,4,7]=918.9730853,
Eta[4,4,4,7]=-0.0005747,
Eta[4,4,7,7]=-518.3041609,
Eta[5,5,5]=530.0147297,
Eta[5,5,5,5]=116.0794891,
Eta[5,5,1]=0.0000883,
Eta[5,5,5,1]=-0.0000410,
Eta[5,5,2]=0.0000771,
Eta[5,5,5,2]=-0.0000239,
Eta[5,5,3]=0.0003674,
Eta[5,5,5,3]=0.0001162,
Eta[5,5,4]=0.0005553,
Eta[5,5,5,4]=0.0001134,
Eta[5,5,6]=-0.0000235,
Eta[5,5,5,6]=-0.0000065,
Eta[5,5,6,6]=25.8007193,
Eta[5,5,7]=124.4661417,
Eta[5,5,5,7]=39.9068989,
Eta[5,5,7,7]=41.1075574,
Eta[6,6,6]=0.0006744,
Eta[6,6,6,6]=553.7524306,
Eta[6,6,1]=0.0002870,
Eta[6,6,6,1]=-0.0000374,
Eta[6,6,2]=0.0003884,
Eta[6,6,6,2]=-0.0003995,
Eta[6,6,3]=-0.0013097,
Eta[6,6,6,3]=-0.0001365,
Eta[6,6,4]=-0.0006630,
Eta[6,6,6,4]=0.0005103,
Eta[6,6,5]=312.6452483,
Eta[6,6,6,5]=0.0000397,
Eta[6,6,7]=-1381.8215359,

Eta[6,6,6,7]=-0.0001652,
Eta[6,6,7,7]=508.7384241,
Eta[7,7,7]=-1272.5841055,
Eta[7,7,7,7]=474.5256434,
Eta[7,7,1]=0.0002432,
Eta[7,7,7,1]=-0.0000149,
Eta[7,7,2]=0.0007570,
Eta[7,7,7,2]=-0.0003581,
Eta[7,7,3]=-0.0015785,
Eta[7,7,7,3]=0.0008112,
Eta[7,7,4]=-0.0010863,
Eta[7,7,7,4]=0.0006761,
Eta[7,7,5]=371.8417661,
Eta[7,7,7,5]=-105.0243738,
Eta[7,7,6]=-0.0002386,
Eta[7,7,7,6]=0.0001543,

Table S17. Q-Chem print out of the harmonic force field for the KS-DFT B97-1/aug-cc-pVTZ calculation of ClF_3.

```
****************************************************************************
**                                                                        **
**                  VIBRATIONAL ANALYSIS                                  **
**                  --------------------                                  **
**                                                                        **
**       VIBRATIONAL FREQUENCIES (CM**-1) AND NORMAL MODES                **
**    FORCE CONSTANTS (mDYN/ANGSTROM) AND REDUCED MASSES (AMU)             **
**              INFRARED INTENSITIES (KM/MOL)                             **
**                                                                        **
****************************************************************************
Mode:                 1                    2                    3
 Frequency:      309.20               322.17               415.76
 Force Cnst:     1.2078               1.5034               1.9708
 Red. Mass:     21.4426              24.5828              19.3515
 IR Active:         YES                  YES                  YES
 IR Intens:      12.422               16.147                0.148
 Raman Active:      YES                  YES                  YES
               X      Y      Z      X      Y      Z      X      Y      Z
 Cl        0.000  0.000 -0.391   0.000  0.591  0.000  -0.149  0.000 -0.000
 F        -0.000  0.000 -0.423  -0.000  0.050  0.000   0.775  0.000  0.000
 F        -0.086 -0.000  0.571  -0.000 -0.569 -0.000  -0.251 -0.000  0.354
 F         0.086 -0.000  0.571  -0.000 -0.569  0.000  -0.251 -0.000 -0.354
 TransDip  0.000  0.000 -0.113   0.000  0.129  0.000  -0.012  0.000 -0.000

 Mode:                4                    5                    6
 Frequency:      532.56               704.12               755.53
 Force Cnst:     3.1842               7.4069               7.6619
 Red. Mass:     19.0555              25.3569              22.7815
 IR Active:         YES                  YES                  YES
 IR Intens:       4.389              503.057               47.941
 Raman Active:      YES                  YES                  YES
               X      Y      Z      X      Y      Z      X      Y      Z
 Cl       -0.000 -0.000 -0.060   0.631 -0.000  0.000   0.000 -0.000 -0.487
 F        -0.000 -0.000  0.226  -0.076 -0.000 -0.000  -0.000 -0.000  0.837
 F        -0.685  0.000 -0.058  -0.543  0.000 -0.058   0.174  0.000  0.029
 F         0.685  0.000 -0.058  -0.543  0.000  0.058  -0.174  0.000  0.029
 TransDip -0.000 -0.000 -0.067   0.718 -0.000  0.000   0.000 -0.000 -0.222
```

Table S18. Q-Chem print out of the anharmonic force field for the KS-DFT B97-1/aug-cc-pVTZ calculation of ClF_3.

```
=================================================================
  ANHARMONIC CUBIC & QUARTIC FORCE FIELD (CM**-1)
_________________________________________________________________
Eta[1,1,1]=-67.5372914783,
 Eta[1,1,1,1]=0.2704059897,
 Eta[1,1,2]=0.0000783874,
 Eta[1,1,1,2]=0.0000021280,
 Eta[1,1,2,2]=0.7819722450,
 Eta[1,1,3]=0.0004005954,
 Eta[1,1,1,3]=-0.0000897612,
 Eta[1,1,3,3]=9.0938593105,
 Eta[1,1,4]=4.8229086752,
 Eta[1,1,1,4]=9.2530740445,
 Eta[1,1,4,4]=-7.0235791664,
 Eta[1,1,5]=-0.0000689221,
 Eta[1,1,1,5]=-0.0000263147,
 Eta[1,1,5,5]=-10.6871170496,
 Eta[1,1,6]=5.4992920466,
 Eta[1,1,1,6]=-2.1954431734,
 Eta[1,1,6,6]=-0.0556777028,
 Eta[2,2,2]=-0.0000276050,
 Eta[2,2,2,2]=-16.6156414046,
 Eta[2,2,1]=10.8278907776,
 Eta[2,2,2,1]=-0.0000163010,
 Eta[2,2,3]=0.0000244642,
 Eta[2,2,2,3]=-0.0000233129,
 Eta[2,2,3,3]=-0.6212729782,
 Eta[2,2,4]=4.6892993467,
 Eta[2,2,2,4]=0.0000089835,
 Eta[2,2,4,4]=-7.9048391131,
 Eta[2,2,5]=0.0005269830,
 Eta[2,2,2,5]=-0.0000765090,
 Eta[2,2,5,5]=-17.8962447143,
 Eta[2,2,6]=-11.0424164486,
 Eta[2,2,2,6]=0.0000011546,
 Eta[2,2,6,6]=-5.3806691523,
 Eta[3,3,3]=-0.0007545988,
 Eta[3,3,3,3]=11.2851831320,
 Eta[3,3,1]=-68.2822337195,
 Eta[3,3,3,1]=0.0000364149,
 Eta[3,3,2]=0.0000593458,
 Eta[3,3,3,2]=-0.0000175675,
 Eta[3,3,4]=-19.9718943883,
 Eta[3,3,3,4]=-0.0000294212,
 Eta[3,3,4,4]=-1.9829068009,
 Eta[3,3,5]=-0.0007530310,
 Eta[3,3,3,5]=3.9626747481,
 Eta[3,3,5,5]=-0.8038058451,
 Eta[3,3,6]=15.3011464962,
 Eta[3,3,3,6]=-0.0000076450,
 Eta[3,3,6,6]=-6.1315648164,
```

Eta[4,4,4]=-97.7681594097,
Eta[4,4,4,4]=14.7562837922,
Eta[4,4,1]=1.3083865033,
Eta[4,4,4,1]=-1.8891227338,
Eta[4,4,2]=-0.0000006755,
Eta[4,4,4,2]=0.0000016608,
Eta[4,4,3]=0.0000599954,
Eta[4,4,4,3]=-0.0000081007,
Eta[4,4,5]=-0.0012019815,
Eta[4,4,4,5]=-0.0001611427,
Eta[4,4,5,5]=20.3407240431,
Eta[4,4,6]=46.7177237243,
Eta[4,4,4,6]=-1.5677617090,
Eta[4,4,6,6]=6.8946333193,
Eta[5,5,5]=-0.0060210866,
Eta[5,5,5,5]=31.8368090906,
Eta[5,5,1]=-0.3619954060,
Eta[5,5,5,1]=0.0007384620,
Eta[5,5,2]=0.0000004774,
Eta[5,5,5,2]=0.0000959437,
Eta[5,5,3]=-0.0014207505,
Eta[5,5,5,3]=3.3221450521,
Eta[5,5,4]=-132.6482123005,
Eta[5,5,5,4]=0.0008963862,
Eta[5,5,6]=32.1472895366,
Eta[5,5,5,6]=0.0018675544,
Eta[5,5,6,6]=-3.5441968432,
Eta[6,6,6]=240.0773322141,
Eta[6,6,6,6]=71.4938702414,
Eta[6,6,1]=-12.7140048006,
Eta[6,6,6,1]=-3.7089914265,
Eta[6,6,2]=0.0000151350,
Eta[6,6,6,2]=0.0000047948,
Eta[6,6,3]=0.0016934457,
Eta[6,6,6,3]=0.0009012607,
Eta[6,6,4]=52.5035941852,
Eta[6,6,6,4]=20.9831981823,
Eta[6,6,5]=-0.0077521316,
Eta[6,6,6,5]=-0.0037944890,

Table S19. Q-Chem print out of the harmonic force field for the TAO-DFT B97-1/aug-cc-pVTZ calculation of ClF_3.

```
**************************************************************************
**                                                                      **
**                 VIBRATIONAL ANALYSIS                                 **
**                 --------------------                                 **
**                                                                      **
**     VIBRATIONAL FREQUENCIES (CM**-1) AND NORMAL MODES                **
**   FORCE CONSTANTS (mDYN/ANGSTROM) AND REDUCED MASSES (AMU)           **
**            INFRARED INTENSITIES (KM/MOL)                             **
**                                                                      **
**************************************************************************
Mode:             1                  2                  3
 Frequency:      309.76             321.69             414.43
 Force Cnst:     1.2100             1.4987             1.9584
 Red. Mass:     21.4031            24.5797            19.3534
 IR Active:        YES                YES                YES
 IR Intens:     11.873             15.952              0.154
 Raman Active:      YES                YES                YES
              X      Y      Z        X      Y      Z        X      Y      Z
 Cl      -0.000  0.000 -0.388    0.000  0.591  0.000   -0.149  0.000  0.000
 F        0.000  0.000 -0.428   -0.000  0.050  0.000    0.775  0.000  0.000
 F       -0.086 -0.000  0.571    0.000 -0.569 -0.000   -0.250 -0.000  0.355
 F        0.086 -0.000  0.571   -0.000 -0.569 -0.000   -0.250 -0.000 -0.355
 TransDip  -0.000  0.000 -0.110    0.000  0.128  0.000   -0.013 -0.000  0.000

 Mode:            4                  5                  6
 Frequency:      534.57             705.87             744.15
 Force Cnst:     3.2110             7.4420             7.4402
 Red. Mass:     19.0710            25.3510            22.8040
 IR Active:        YES                YES                YES
 IR Intens:      4.969             494.563            48.998
 Raman Active:      YES                YES                YES
              X      Y      Z        X      Y      Z        X      Y      Z
 Cl      -0.000  0.000  0.067    0.631 -0.000 -0.000   -0.000 -0.000 -0.488
 F       -0.000  0.000 -0.236   -0.075 -0.000  0.000    0.000 -0.000  0.831
 F        0.683 -0.000  0.056   -0.543  0.000 -0.058    0.185  0.000  0.034
 F       -0.683 -0.000  0.056   -0.543  0.000  0.058   -0.185  0.000  0.034
 TransDip  -0.000 -0.000  0.071    0.712 -0.000 -0.000   -0.000  0.000 -0.224
```

Table S20. Q-Chem print out of the anharmonic force field for the TAO-DFT B97-1/aug-cc-pVTZ calculation of ClF_3.

===
ANHARMONIC CUBIC & QUARTIC FORCE FIELD (CM**-1)

Eta[1,1,1]=-67.7729015,
Eta[1,1,1,1]=0.3035355,
Eta[1,1,2]=0.0001761,
Eta[1,1,1,2]=-0.0000048,
Eta[1,1,2,2]=0.5724240,
Eta[1,1,3]=-0.0001547,
Eta[1,1,1,3]=0.0000275,
Eta[1,1,3,3]=9.1985727,
Eta[1,1,4]=-4.9110640,
Eta[1,1,1,4]=-9.2093468,
Eta[1,1,4,4]=-7.0751337,
Eta[1,1,5]=-0.0000992,
Eta[1,1,1,5]=0.0001374,
Eta[1,1,5,5]=-11.0519458,
Eta[1,1,6]=5.8319463,
Eta[1,1,1,6]=-2.6247124,
Eta[1,1,6,6]=-0.1217126,
Eta[2,2,2]=-0.0001183,
Eta[2,2,2,2]=-16.7457863,
Eta[2,2,1]=10.1484626,
Eta[2,2,2,1]=-0.0000732,
Eta[2,2,3]=-0.0000111,
Eta[2,2,2,3]=-0.0000017,
Eta[2,2,3,3]=-0.9035756,
Eta[2,2,4]=-5.7320209,
Eta[2,2,2,4]=-0.0000215,
Eta[2,2,4,4]=-8.4139473,
Eta[2,2,5]=-0.0002065,
Eta[2,2,2,5]=-0.0000142,
Eta[2,2,5,5]=-18.3167300,
Eta[2,2,6]=-9.3735529,
Eta[2,2,2,6]=0.0000344,
Eta[2,2,6,6]=-5.8550802,
Eta[3,3,3]=0.0003092,
Eta[3,3,3,3]=11.0337141,
Eta[3,3,1]=-69.3679357,
Eta[3,3,3,1]=-0.0000210,
Eta[3,3,2]=0.0000893,
Eta[3,3,3,2]=0.0000004,
Eta[3,3,4]=19.7085494,
Eta[3,3,3,4]=-0.0000104,
Eta[3,3,4,4]=-2.3077394,
Eta[3,3,5]=0.0002608,
Eta[3,3,3,5]=4.2329886,
Eta[3,3,5,5]=-1.2229388,
Eta[3,3,6]=17.2512293,
Eta[3,3,3,6]=-0.0000149,
Eta[3,3,6,6]=-6.2722855,

Eta[4,4,4]=96.4018197,
Eta[4,4,4,4]=13.7774577,
Eta[4,4,1]=1.3509449,
Eta[4,4,4,1]=2.0344807,
Eta[4,4,2]=-0.0000636,
Eta[4,4,4,2]=-0.0000084,
Eta[4,4,3]=-0.0000234,
Eta[4,4,4,3]=-0.0000027,
Eta[4,4,5]=0.0005755,
Eta[4,4,4,5]=-0.0000189,
Eta[4,4,5,5]=20.1691504,
Eta[4,4,6]=50.2098942,
Eta[4,4,4,6]=1.6274259,
Eta[4,4,6,6]=7.6100055,
Eta[5,5,5]=0.0025990,
Eta[5,5,5,5]=29.8496991,
Eta[5,5,1]=-0.9034588,
Eta[5,5,5,1]=-0.0002658,
Eta[5,5,2]=0.0000107,
Eta[5,5,5,2]=0.0000167,
Eta[5,5,3]=0.0005196,
Eta[5,5,5,3]=3.7386052,
Eta[5,5,4]=133.5318034,
Eta[5,5,5,4]=0.0003630,
Eta[5,5,6]=37.8960001,
Eta[5,5,5,6]=-0.0007466,
Eta[5,5,6,6]=-4.5780145,
Eta[6,6,6]=244.1972704,
Eta[6,6,6,6]=72.2911742,
Eta[6,6,1]=-16.8461463,
Eta[6,6,6,1]=-4.2939852,
Eta[6,6,2]=0.0000496,
Eta[6,6,6,2]=0.0000327,
Eta[6,6,3]=-0.0006341,
Eta[6,6,6,3]=-0.0003608,
Eta[6,6,4]=-55.2386565,
Eta[6,6,6,4]=-22.7275804,
Eta[6,6,5]=0.0028483,
Eta[6,6,6,5]=0.0015552,
